# Supplementary material for: Mitochondrial Genomes from Fungal the Entomopathogenic Moelleriella Genus Reveals Evolutionary History, Intron Dynamics and Phylogeny
Source: J Fungi (Basel). 2025 Jan 24;11(2):94. doi: 10.3390/jof11020094 (PMC11856489; doi:10.3390/jof11020094)
Supplement: Supplementary file 1 [file jof-11-00094-s001.zip › jof-3365993-supplementary.pdf]

Table S1 Comparison on mitogenomes among 27 Hypocreales species

	Moelleriella zhongdongii	*Moelleriella libera*	*Moelleriella raciborskii*	*Moelleriella gracilispora*	*Moelleriella oxystoma*	*Moelleriella* sp.C9	*Moelleriella* sp.C3	*Gibberella moniliformis*	*Memnoniella echinata*
GC content (%)	26.50%	27.90%	28.20%	26.60%	27.80%	26.60%	26.10%	32.60%	25.50%
AT content (%)	73.50%	72.10%	71.80%	73.40%	72.20%	73.40%	73.90%	67.40%	74.50%
GC skew (%)	9.43%	10.39%	9.54%	10.53%	10.47%	11.28%	10.69%	9.82%	14.50%
AT skew (%)	3.27%	2.91%	3.34%	5.72%	4.71%	3.95%	4.34%	1.18%	-2.28%
Number of tRNAs	25	27	25	26	27	26	26	25	23
Number of introns	16	9	14	34	34	16	16	5	2
Number of un_ORFs	5	5	1	9	12	5	6	2	1
Number of core genes	15	15	15	15	15	15	15	15	15
Mitogenome length (bp)	57023	40828	45471	79556	95666	53829	54896	53753	28743
Length of core genes (bp)	16251	14409	14415	15480	15288	16251	14784	14622	14442
Length of HEGs (bp)	12978	4461	4032	23601	25296	12978	10945	0	2193
Length of un_ORFs (bp)	3990	3330	1290	9174	9585	3990	4650	8175	738
Length of RNA regions (bp)	6335	6490	6337	6433	6483	6388	6394	7090	6698
Length of intronic regions (bp)	21260	12078	14373	40367	48421	19819	19288	8233	3905
Length of intergenic regions (bp)	9187	4521	9056	8102	15889	7381	9780	15633	2960
Length of core genes (%)	28.50%	35.29%	31.70%	19.46%	15.98%	30.19%	26.93%	27.20%	50.25%
Length of un_ORFs (%)	7.00%	8.16%	2.84%	11.53%	10.02%	7.41%	8.47%	15.21%	2.57%
Length of HEGs (%)	22.76%	10.93%	8.87%	29.67%	26.44%	24.11%	19.94%	0.00%	7.63%
Length of RNA regions (%)	11.11%	15.90%	13.94%	8.09%	6.78%	11.87%	11.65%	13.19%	23.30%
Length of intronic regions (%)	37.28%	29.58%	31.61%	50.74%	50.61%	36.82%	35.14%	15.32%	13.59%
Length of intergenic regions (%)	16.11%	11.07%	19.92%	10.18%	16.61%	13.71%	17.82%	29.08%	10.30%

	Purpureocillium takamizusanense	*Fusarium ussurianum*	*Clonostachys byssicola*	*Cordyceps militaris*	*Trichoderma afroharzianum*	*Metarhizium album*	*Orbiocrella petchii*	*Akanthomyces lecanii*	*Beauveria pseudobassiana*
GC content (%)	26.50%	31.40%	28.50%	26.80%	27.70%	26.00%	28.10%	27.10%	27.50%
AT content (%)	73.50%	68.60%	71.50%	73.20%	72.30%	74.00%	71.90%	72.90%	72.50%
GC skew (%)	-0.41%	3.79%	1.82%	1.09%	-0.14%	3.24%	9.61%	-1.92%	-1.38%
AT skew (%)	12.78%	8.28%	11.58%	13.43%	9.75%	13.08%	-0.42%	12.18%	10.55%
Number of tRNAs	23	25	24	24	20	24	22	24	23
Number of introns	1	26	13	8	4	23	2	1	2
Number of un_ORFs	7	4	4	0	1	7	0	0	1
Number of core genes	15	15	15	15	15	15	15	15	15
Mitogenome length (bp)	33113	99850	51551	33227	29517	68425	23794	24577	28258
Length of core genes (bp)	14475	14565	14679	14343	14439	14436	14427	14367	14481
Length of HEGs (bp)	0	36996	8343	0	0	21219	1662	0	1263
Length of un_ORFs (bp)	5406	7953	2310	0	148	4941	0	0	1089
Length of RNA regions (bp)	7081	7007	6420	6590	6028	6486	6294	6497	6620
Length of intronic regions (bp)	1552	52748	17825	9868	3781	31516	2306	1711	1611
Length of intergenic regions (bp)	4599	17577	10317	2426	5121	11046	767	2002	4457
Length of core genes (%)	43.71%	14.59%	28.47%	43.17%	48.92%	21.10%	60.63%	58.46%	51.25%
Length of un_ORFs (%)	16.33%	7.96%	4.48%	0.00%	0.50%	7.22%	0.00%	0.00%	3.85%
Length of HEGs (%)	0.00%	37.05%	16.18%	0.00%	0.00%	31.01%	6.98%	0.00%	4.47%
Length of RNA regions (%)	21.38%	7.02%	12.45%	19.83%	20.42%	9.48%	26.45%	26.44%	23.43%
Length of intronic regions (%)	4.69%	52.83%	34.58%	29.70%	12.81%	46.06%	9.69%	6.96%	5.70%
Length of intergenic regions (%)	13.89%	17.60%	20.01%	7.30%	17.35%	16.14%	3.22%	8.15%	15.77%

	Nectria cinnabarina	*Stachybotrys chartarum*	*Stachybotrys chlorohalonata*	*Metacordyceps chlamydosporia*	*Zelopaecilomyces penicillatus*	*Samsoniella hepiali*	*Pleurocordyceps sinensis*	*Parengyodontium album*	*Tolypocladium cylindrosporum*
GC content (%)	28.70%	26.30%	26.20%	28.30%	27.50%	26.60%	25.50%	25.90%	27.00%
AT content (%)	71.30%	73.70%	73.80%	71.70%	72.50%	73.40%	74.50%	74.10%	73.00%
GC skew (%)	9.41%	-1.63%	-0.95%	10.25%	1.24%	12.78%	10.59%	13.51%	11.52%
AT skew (%)	4.35%	11.79%	11.79%	-0.84%	11.27%	-2.45%	0.40%	-0.94%	1.92%
Number of tRNAs	23	25	25	23	22	23	23	21	24
Number of introns	18	2	3	1	1	1	2	4	5
Number of un_ORFs	0	2	2	0	2	0	5	2	4
Number of core genes	15	15	15	15	15	15	15	15	15
Mitogenome length (bp)	69895	30745	31375	25615	27480	24246	31841	28081	34698
Length of core genes (bp)	15642	14349	14352	14385	15702	14367	14421	14307	14394
Length of HEGs (bp)	0	2139	3102	0	0	0	951	0	5499
Length of un_ORFs (bp)	0	1668	1644	0	870	0	4062	678	2571
Length of RNA regions (bp)	7443	6691	6659	6205	6332	6457	8112	6036	6502
Length of intronic regions (bp)	27231	3884	5123	1652	1736	1692	2679	4139	6419
Length of intergenic regions (bp)	19579	4153	3597	3373	2840	1730	2567	2921	4812
Length of core genes (%)	22.38%	46.67%	45.74%	56.16%	57.14%	59.26%	45.29%	50.95%	41.48%
Length of un_ORFs (%)	0.00%	5.43%	5.24%	0.00%	3.17%	0.00%	12.76%	2.41%	7.41%
Length of HEGs (%)	0.00%	6.96%	9.89%	0.00%	0.00%	0.00%	2.99%	0.00%	15.85%
Length of RNA regions (%)	10.65%	21.76%	21.22%	24.22%	23.04%	26.63%	25.48%	21.49%	18.74%
Length of intronic regions (%)	38.96%	12.63%	16.33%	6.45%	6.32%	6.98%	8.41%	14.74%	18.50%
Length of intergenic regions (%)	28.01%	13.51%	11.46%	13.17%	10.33%	7.14%	8.06%	10.40%	13.87%

Table S2 Characterization of the mitogenomes of 7 *Moelleriella* species

Moelleriella zhongdongii

Gene	Start	Stop	Length	Notes
rnl	1	3483	3483	
rps3	1006	2331	1326	
trnT	3491	3561	71	
trnE	4014	4087	74	
trnM	4091	4161	71	
trnM	4200	4273	74	
trnL	4306	4388	83	
trnA	4459	4531	73	
trnF	7250	7322	73	
trnK	7332	7404	73	
trnL	7411	7472	62	
trnQ	9791	9863	73	
trnH	9916	9988	73	
trnM	10332	10403	72	
nad2	10775	12523	1749	
orf447	13782	15125	1344	LAGLIDADG endonuclease
nad3	15147	15509	363	
atp9	15630	15853	224	
cox2	15990	19765	3776	
exon1	15990	16217	228	
orf294	16206	17090	885	GIY-YIG endonuclease
exon2	17326	17748	423	
orf314	18621	19565	945	hypothetical protein
exon3	19667	19765	99	
trnR	19808	19878	71	
nad4L	19988	20257	270	
nad5	20257	23280	3024	
exon1	20257	20682	426	
orf115	21077	21424	348	hypothetical protein
exon2	21718	23280	1563	
cob	24168	27585	3418	
exon1	24186	24560	375	
orf284	24570	25424	855	GIY-YIG endonuclease
exon2	25747	25843	97	
exon3	26906	27585	680	
trnC	27685	27755	71	
cox1	28083	35597	7515	
exon1	28083	28294	212	
orf328	28290	29276	987	LAGLIDADG endonuclease
exon2	29555	30209	655	
orf353	30198	31259	1062	LAGLIDADG endonuclease
exon3	31344	31533	190	
orf420	31536	32798	1263	LAGLIDADG endonuclease
exon4	32812	32879	68	
orf365	32847	33944	1098	hypothetical protein

Moelleriella libera

Gene	Start	Stop	Length	Notes
rns	427	1937	1511	
trnY	1978	2060	83	
trnD	2061	2133	73	
trnS	2144	2225	82	
trnN	2361	2424	64	
cox3	2471	6372	3902	
exon1	2471	2689	219	
orf163	2678	3196	519	LAGLIDADG endonuclease
orf6	3488	3508	21	
orf238	4085	4801	717	LAGLIDADG endonuclease
exon2	4962	5373	412	
exon3	6194	6372	179	
orf408	6599	7825	1227	hypothetical protein
orf174	8224	8748	525	hypothetical protein
orf278	8805	9641	837	hypothetical protein
trnG	9672	9742	71	
trnG	9745	9815	71	
trnL	9841	9915	75	
orf159	10096	10575	480	hypothetical protein
nad6	10846	11523	678	
trnV	11577	11649	73	
trnL	11651	11722	72	
trnS	11726	11812	87	
trnW	11864	11935	72	
trnP	12022	12094	73	
rnl	12181	17015	4835	
exon1	12181	14595	2415	
rps3	14869	16221	1353	
exon2	16459	17015	557	
trnT	17022	17092	71	
trnE	17107	17179	73	
trnM	17180	17250	71	
trnM	17279	17351	73	
trnL	17508	17589	82	
trnA	17920	17991	72	
orf86	18395	18655	261	hypothetical protein
trnF	18864	18936	73	
trnK	18943	19015	73	
trnL	19018	19082	65	
trnQ	19339	19411	73	
trnH	19420	19493	74	
trnM	19743	19814	72	
nad2	19904	21571	1668	
nad3	21572	21985	414	
atp9	22223	22447	225	

Moelleriella raciborskii

Gene	Start	Stop	Length	Notes
trnF	493	565	73	
trnK	572	644	73	
trnL	647	711	65	
trnQ	968	1040	73	
trnH	1049	1122	74	
trnM	1418	1489	72	
nad2	1580	4561	2982	
exon1	1580	3214	1635	
exon2	4529	4561	33	
nad3	4562	4975	414	
atp9	5229	5450	222	
cox2	5604	8719	3116	
exon1	5604	5831	228	
orf328	5820	6806	987	GIY-YIG endonuclease
exon2	7041	7169	129	
orf274	7158	7982	825	LAGLIDADG endonuclease
exon3	8327	8719	393	
trnR	8912	8982	71	
nad4L	9043	9312	270	
nad5	9312	14167	4856	
exon1	9312	9737	426	
exon2	10791	11074	284	
exon3	12889	14167	1279	
cob	14401	15570	1170	
trnC	15629	15698	70	
cox1	15629	15698	70	
exon1	16030	16311	282	
exon2	17566	17898	333	
exon3	19102	19218	117	
orf312	19225	20163	939	LAGLIDADG endonuclease
exon4	20278	20602	325	
orf419	20605	21864	1260	LAGLIDADG endonuclease
exon5	21878	22407	530	
trnS	22526	22596	71	
nad1	22765	24411	1647	
exon1	22765	23400	636	
exon2	23938	24411	474	
nad4	25396	26853	1458	
atp8	26999	27145	147	
atp6	27242	28021	780	
rns	28574	31372	2799	
exon1	28574	29734	1161	
orf429	29679	30968	1290	hypothetical protein
exon2	31023	31372	350	
trnY	31413	31495	83	

Moelleriella gracilispora

Gene	Start	Stop	Length	Notes
cox1	78638	12964	13833	
exon1	78638	78688	51	
orf532	109	1707	1599	hypothetical protein
exon2	1838	1998	161	
orf341	1994	3025	1032	hypothetical protein
exon3	3308	3710	403	
orf349	3687	4736	1050	hypothetical protein
exon4	4855	4948	94	
orf330	5106	6098	993	hypothetical protein
exon5	6600	6610	11	
orf314	6614	7555	942	LAGLIDADG endonuclease
exon6	7598	7608	11	
orf314	7616	8554	939	LAGLIDADG endonuclease
exon7	8637	8772	136	
orf343	8761	9804	1044	LAGLIDADG endonuclease
exon8	9889	10078	190	
orf419	10081	11340	1260	GIY-YIG endonuclease
exon9	11354	11421	68	
orf342	11389	12450	1062	LAGLIDADG endonuclease
exon10	12503	12964	462	
trsS	13057	13127	71	
nad1	13289	17412	4124	
exon1	13289	13432	144	
orf476	13412	14863	1452	GIY-YIG endonuclease
exon2	15360	15851	492	
orf106	16051	16371	321	GIY-YIG endonuclease
exon3	16939	17412	474	
orf256	17515	18279	765	hypothetical protein
nad4	18552	20009	1458	
atp8	20178	20342	165	
atp6	20506	24190	3685	
exon1	20506	20855	350	
orf347	20851	21900	1050	LAGLIDADG endonuclease
exon2	2293	22420	20128	
orf302	23041	23949	909	GIY-YIG endonuclease
exon3	23983	24190	208	
orf281	25411	26256	846	hypothetical protein
rns	26731	29516	2786	
exon1	26731	27879	1149	
exon2	29168	29516	349	
trnY	29559	29641	83	
trnD	29642	29714	73	
trnS	29737	29819	83	
trnN	30007	30070	64	
cox3	30128	34661	4534	

Moelleriella oxystoma

Gene	Start	Stop	Length	Notes
trnW	39	110	72	
rnl	2149	10000	7852	
exon1	2149	3513	1365	
exon2	5148	6193	1046	
rps3	6468	7805	1338	
orf152	8667	9125	459	LAGLIDADG endonuclease
exon3	9430	10000	571	
trnT	10007	10077	71	
trnE	10093	10166	74	
trnM	10167	10237	71	
trnM	12498	12825	328	
trnL	12743	12825	83	
trnA	13500	13572	73	
orf112	13961	14299	339	hypothetical protein
trnA	14355	14425	71	
orf288	14475	15341	867	hypothetical protein
orf418	15732	16988	1257	hypothetical protein
orf163	17050	17541	492	hypothetical protein
orf227	18264	18947	684	hypothetical protein
orf315	19397	20344	948	hypothetical protein
trnA	20470	20541	72	
orf83	20895	21146	252	hypothetical protein
trnF	21494	21566	73	
trnK	21573	21645	73	
trnL	21649	21732	84	
trnQ	22401	22473	73	
trnH	22477	22548	72	
trnM	23395	23466	72	
nad2	23575	30220	6646	
exon1	23575	23832	258	
orf331	24128	25123	996	LAGLIDADG endonuclease
exon2	25161	25280	120	
orf430	25269	26561	1293	LAGLIDADG endonuclease
exon3	26613	26990	378	
orf196	26967	27557	591	LAGLIDADG endonuclease
exon4	28352	30220	1869	
orf446	30522	31862	1341	LAGLIDADG endonuclease
nad3	31908	32243	336	
atp9	32367	32591	225	
cox2	32722	40084	7363	
exon1	32722	32949	228	
orf293	32938	33819	882	GIY-YIG endonuclease
exon2	34059	34091	33	
orf222	34687	35355	669	LAGLIDADG endonuclease
exon3	35398	35493	96	

Moelleriella sp.C9

Gene	Start	Stop	Length	Notes
rnl	53093	7481	7636	
exon1	53093	53675	583	
orf244	261	995	735	GIY-YIG endonuclease
exon2	1082	1273	192	
exon3	2611	4245	1635	
rps3	4526	6808	2283	
exon4	6929	7481	553	
trnT	7488	7558	71	
orf115	7710	8057	348	hypothetical protein
trnE	9255	9327	73	
trnM	9328	9398	71	
trnM	9451	9523	73	
trnL	9568	9650	83	
trnA	10020	10092	73	
trnF	10388	10460	73	
trnK	10467	10539	73	
trnL	10716	10798	83	
trnQ	11214	11286	73	
trnH	11295	11368	74	
orf294	11651	12535	885	hypothetical protein
orf235	12750	13457	708	hypothetical protein
trnM	13778	13849	72	
trnL	13989	14060	72	
nad2	14107	15786	1680	
orf447	15787	17130	1344	hypothetical protein
nad3	17152	17511	360	
atp9	17765	17989	225	
cox2	18131	21154	3024	
exon1	18131	18358	228	
orf297	18347	19240	894	GIY-YIG endonuclease
exon2	19475	19603	129	
orf274	19592	20416	825	LAGLIDADG endonuclease
exon3	20762	21154	393	
trnR	21257	21325	69	
nad4l	21405	23124	1720	
exon1	21405	21643	239	
orf338	21624	22640	1017	LAGLIDADG endonuclease
exon2	23094	23124	31	
nad5	23124	26242	3119	
exon1	23124	23549	426	
orf113	23922	24263	342	LAGLIDADG endonuclease
exon2	24680	26242	1563	
cob	26438	29933	3496	
exon1	26438	26830	393	
orf282	26825	27673	849	GIY-YIG endonuclease

Moelleriella sp.C3

Gene	Start	Stop	Length	Notes
rnl	46625	156		
exon1	46625	47399		
orf163	48073	48561		GIY-YIG endonuclease
exon2	48718	49307		
exon3	50794	51841		
rps3	52120	53769		
exon4	54498	54896		
exon5	1	156		
trnT	163	233		
orf222	1167	1835		hypothetical protein
trnE	1977	2049		
trnM	2050	2120		
trnM	2176	2248		
trnL	2291	2373		
trnA	3047	3118		
trnF	3122	3194		
trnK	3201	3273		
trnL	3450	3532		
trnQ	3961	4033		
trnH	4042	4115		
trnM	6562	6633		
trnL	6773	6633		
nad2	6891	8585		
nad3	8586	8999		
atp9	9252	10780		
exon1	9252	9432		
orf258	9706	10482		GIY-YIG endonuclease
exon2	10737	10780		
cox2	10877	11149		
exon1	10877	11149		
orf290	11138	12022		GIY-YIG endonuclease
exon2	12257	12778		
trnR	12880	12950		
nad4L	13029	13298		
nad5	13298	16373		
exon1	13298	13723		
exon2	14811	16373		
cob	16568	19822		
exon1	16568	16960		
orf282	16955	17809		GIY-YIG endonuclease
exon2	18132	18561		
orf116	19096	19446		LAGLIDADG endonuclease
exon3	19476	19822		
trnC	19897	19966		
cox1	20296	28770		

exon5	35046	35507	462	
trnS	35546	35616	71	
nad1	35758	38178	2421	
exon1	35758	36048	291	
exon2	37360	38178	819	
nad4	38271	39728	1458	
atp8	39830	39976	147	
atp6	40048	42204	2157	
exon1	40048	40400	353	
orf349	40420	41469	1050	LAGLIDADG endonuclease
exon2	41769	42204	436	
rns	42625	44129	1505	
trnY	44172	44256	85	
trnD	44642	44714	73	
trnN	45581	45644	64	
cox3	45695	47608	1914	
exon1	45695	45913	219	
orf306	45902	46822	921	hypothetical protein
exon2	47018	47608	591	
trnG	47653	47723	71	
nad6	47838	48506	669	
trnV	49669	49740	72	
trnL	50510	50581	72	
trnS	50585	50671	87	
trnW	50691	50762	72	
trnP	51467	51539	73	
rnl	51602	54905	3304	
exon1	51602	52177	576	
orf243	52558	53289	732	hypothetical protein
exon2	53377	53559	183	
orf166	53983	54483	501	hypothetical protein
exon3	54775	54905	131	
orf314	55200	56144	945	hypothetical protein

cox2	22601	24562	1962	
exon1	22601	22828	228	
orf329	22817	23806	990	GIY-YIG endonuclease
exon2	24041	24562	522	
trnR	24719	24789	71	
nad4L	24850	25119	270	
nad5	25119	28866	3748	
exon1	25119	25828	710	
exon2	27588	28866	1279	
cob	29103	32391	3289	
exon1	29103	29495	393	
orf295	29490	30377	888	GIY-YIG endonuclease
exon2	30700	31129	430	
orf116	31669	32019	351	LAGLIDADG endonuclease
exon3	32045	32391	347	
trnC	32452	32521	70	
cox1	32859	34445	1587	
trnS	34541	34611	71	
nad1	34779	37947	3169	
exon1	34779	35166	388	
orf331	35193	36188	996	LAGLIDADG endonuclease
exon2	36253	36500	248	
exon3	37474	37947	474	
nad4	38094	39551	1458	
atp8	39679	39825	147	
atp6	39922	40701	780	

trnD	31496	31568	73	
trnS	31578	31660	83	
trnN	31808	31879	72	
cox3	32967	34911	1945	
exon1	32967	33185	219	
orf6	33955	33975	21	hypothetical protein
exon2	34321	34911	591	
trnG	34944	35014	71	
nad6	35118	35795	678	
trnV	35849	35921	73	
trnI	35923	35994	72	
trnS	35998	36084	87	
trnW	36136	36207	72	
trnP	38157	38229	73	
rnl	38316	44026	5711	
exon1	38316	39942	1627	
exon2	40795	41581	787	
rps3	41852	43213	1362	
exon3	43458	44026	569	
trnT	44033	44103	71	
trnE	44118	44190	73	
trnM	44191	44261	71	
trnL	44513	44594	82	
trnA	44927	44998	72	

exon1	30128	30346	219	
orf172	30335	30865	531	LAGLIDADG endonuclease
exon2	31490	31603	114	
orf437	31583	32917	1335	LAGLIDADG endonuclease
exon3	32963	33260	298	
orf144	33263	33697	435	LAGLIDADG endonuclease
orf155	33756	34223	468	LAGLIDADG endonuclease
exon4	34483	34661	179	
trnG	34682	34752	71	
nad6	34856	35533	678	
trnV	35576	35648	73	
trnL	35650	35721	72	
trns	35747	35833	87	
trnW	35889	35960	72	
trnP	36413	36485	73	
rnl	36567	47985	11419	
exon1	36567	37149	583	
orf244	37568	38302	735	GIY-YIG endonuclease
exon2	38389	38589	201	
orf120	39100	39465	366	GIY-YIG endonuclease
exon3	39955	40544	590	
orf314	41017	41952	936	LAGLIDADG endonuclease
exon4	42022	42418	397	
orf358	42530	43609	1080	GIY-YIG endonuclease
exon5	44087	44736	650	
rps3	45017	46450	1434	
exon6	47431	47985	555	
trnT	47992	48062	71	
orf515	48202	49752	1551	hypothetical protein
orf249	50104	50853	750	hypothetical protein
trnE	50973	51045	73	
trnM	51046	51116	71	
trnM	51132	51204	73	
trnL	51205	51287	83	
trnA	51639	51711	73	
trnF	52011	52083	73	
trnK	52090	52162	73	
trnL	52404	52486	83	
trnQ	52701	52733	33	
trnH	52782	52855	74	
trnM	53824	53895	72	
trnM	54035	54107	73	
nad2	54153	56804	2652	
nad3	57104	57517	414	
atp9	57766	59069	1304	
exon1	57766	57946	181	
orf117	58658	59011	354	GIY-YIG endonuclease
exon2	59026	59069	44	

orf647	35482	37425	1944	LAGLIDADG endonuclease
exon4	37767	38060	294	
orf312	38780	39718	939	GIY-YIG endonuclease
exon5	39986	40084	99	
trnR	40158	40228	71	
nad4L	40313	41967	1655	
exon1	40313	40551	239	
exon2	41937	41967	31	
nad5	41967	46543	4577	
exon1	41967	42392	426	
exon2	43493	43776	284	
exon3	45265	46543	1279	
cob	46742	54039	7298	
exon1	46742	46896	155	
orf281	47358	48203	846	LAGLIDADG endonuclease
exon2	48226	48271	46	
orf446	48448	49788	1341	LAGLIDADG endonuclease
exon3	49820	50011	192	
orf286	50006	50866	861	LAGLIDADG endonuclease
exon4	51189	51285	97	
orf296	51276	52166	891	GIY-YIG endonuclease
exon5	52443	52885	443	
orf115	53317	53664	348	LAGLIDADG endonuclease
exon6	53693	54039	347	
trnC	54186	54255	70	
cox1	54591	63116	8526	
exon1	54591	54802	212	
orf330	54798	55790	993	hypothetical protein
exon2	56071	56473	403	
orf340	56477	57499	1023	LAGLIDADG endonuclease
exon3	57618	57733	116	
orf325	57705	58682	978	LAGLIDADG endonuclease
exon4	58765	58900	136	
orf356	58892	59962	1071	LAGLIDADG endonuclease
exon5	60068	60257	190	
orf416	60260	61510	1251	GIY-YIG endonuclease
exon6	61523	61590	68	
orf338	61594	62610	1017	hypothetical protein
exon7	62655	63116	462	
trnS	63203	63273	71	
nad1	63431	71568	8138	
exon1	63431	63574	144	
orf269	63790	64599	810	LAGLIDADG endonuclease
exon2	66496	66642	147	
exon3	67903	68032	130	
orf565	68026	69723	1698	hypothetical protein
exon4	69826	70040	215	
orf277	70240	71073	834	GIY-YIG endonuclease

exon2	27996	28092	97	
orf297	28083	28976	894	GIY-YIG endonuclease
exon3	29254	29933	680	
trnC	30008	30077	70	
cox1	30406	40549	10144	
exon1	30406	30617	212	
orf338	30613	31629	1017	GIY-YIG endonuclease
exon2	31910	32312	403	
orf344	32304	33338	1035	LAGLIDADG endonuclease
exon3	33457	33550	94	
orf310	33550	34482	933	LAGLIDADG endonuclease
exon4	34952	34973	22	
orf327	34939	35922	984	LAGLIDADG endonuclease
exon5	36005	36330	326	
orf419	36333	37592	1260	LAGLIDADG endonuclease
exon6	37606	37829	224	
orf309	38347	39276	930	LAGLIDADG endonuclease
exon7	39296	40549	1254	
trnS	40653	40723	71	
nad1	40877	43907	3031	
exon1	40877	41020	144	
orf139	41227	41646	420	GIY-YIG endonuclease
orf234	41739	42443	705	hypothetical protein
exon2	42942	43907	966	
nad4	44033	45490	1458	
atp8	45660	45806	147	
atp6	45991	46776	786	
orf280	46943	47785	843	GIY-YIG endonuclease
rns	48277	49776	1500	
trnY	49821	49903	83	
trnD	49904	49976	73	
trnS	49999	50081	83	
trnN	50258	50321	64	
cox3	50379	51188	810	
trnG	51209	51279	71	
nad6	51383	52060	678	
trnV	52103	52175	73	
trnI	52177	52248	72	
trnS	52273	52359	87	
trnW	52148	52489	342	
trnP	52938	53010	73	

exon1	20296	20507	
orf331	20503	21504	GIY-YIG endonuclease
exon2	21785	21853	
orf356	21849	22925	hypothetical protein
exon3	24087	24536	
orf315	24508	25485	LAGLIDADG endonuclease
exon4	25568	25893	
orf416	25896	27146	GIY-YIG endonuclease
exon5	27160	27227	
orf342	27231	28256	LAGLIDADG endonuclease
exon6	28309	28770	
trnS	28861	28931	
nad1	29090	33179	
exon1	29090	29233	
orf476	29231	30664	GIY-YIG endonuclease
exon2	31159	31650	
orf278	31850	32686	GIY-YIG endonuclease
exon3	32706	33179	
orf256	33282	34046	hypothetical protein
nad4	34319	35776	
atp8	35946	36092	
atp6	36275	37060	
orf165	37583	38080	hypothetical protein
rns	38572	40071	
orf195	40265	40855	hypothetical protein
orf349	41155	42204	hypothetical protein
trnY	42252	42334	
trnD	42335	42407	
trnS	42430	42512	
trnN	42689	42752	
cox3	42808	44716	
exon1	42808	43026	
orf302	43015	43935	LAGLIDADG endonuclease
orf424	43796	43816	LAGLIDADG endonuclease
exon2	44126	44716	
trnG	44737	44807	
nad6	44911	45588	
trnV	45630	45702	
trnI	45704	45775	
trnS	45800	45886	
trmW	45945	46016	
trnP	46468	46540	

cox2	59211	65476	6266	
exon1	59211	59438	228	
orf300	59427	60341	915	GIY-YIG endonuclease
exon2	60576	60608	33	
orf191	61298	61873	576	LAGLIDADG endonuclease
exon3	61916	62011	96	
orf272	62000	62830	831	LAGLIDADG endonuclease
exon4	63173	63466	294	
orf312	64341	65279	939	GIY-YIG endonuclease
exon5	65378	65476	99	
trnR	65578	65648	71	
nad4L	65727	65966	240	
exon1	65727	65965	239	
orf333	65946	66968	1023	LAGLIDADG endonuclease
exon2	67424	67454	31	
nad5	67454	70480	3027	
exon1	67454	67879	426	
orf247	67859	68623	765	hypothetical protein
exon2	68918	70480	1563	
cob	70675	78164	7490	
exon1	70675	70875	201	
orf203	71060	71671	612	LAGLIDADG endonuclease
orf238	71731	72447	717	LAGLIDADG endonuclease
exon2	72475	72666	192	
orf282	72661	73515	855	GIY-YIG endonuclease
exon3	73838	73934	97	
orf293	73925	74848	924	LAGLIDADG endonuclease
exon4	75096	75111	16	
orf280	75628	76479	852	hypothetical protein
exon5	76519	76835	317	
exon6	77818	78164	347	
trnC	78239	78308	70	
exon5	71092	71568	477	
nad4	72525	75354	2830	
exon1	72525	73029	505	
exon2	74402	75354	953	
atp8	75468	75614	147	
atp6	75707	79439	3733	
exon1	75707	76056	350	
orf355	76052	77119	1068	LAGLIDADG endonuclease
exon2	77426	77653	228	
orf303	78276	79187	912	GIY-YIG endonuclease
exon3	79232	79439	208	
orf194	79793	80377	585	hypothetical protein
rns	80936	82441	1506	
trnY	83421	83503	83	
trnD	83504	83576	73	
trnN	85024	85095	72	
cox3	85147	89859	4713	
exon1	85147	85363	217	
orf306	85354	86274	921	LAGLIDADG endonuclease
exon2	86463	86795	333	
orf371	86801	87916	1116	LAGLIDADG endonuclease
exon3	88375	88455	81	
exon4	89681	89859	179	
trnG	89884	89955	72	
nad6	90053	92600	2548	
exon1	90053	90285	233	
orf504	90275	91789	1515	LAGLIDADG endonuclease
exon2	92159	92600	442	
trnT	92672	92744	73	
orf131	92772	93167	396	LAGLIDADG endonuclease
trnV	93606	93677	72	
orf150	94574	95026	453	
trnI	95342	95413	72	
trnS	95430	95516	87	
trnW	95578	95649	72	

The ORFs with blue color indicate that these ORFs were located in introns, while ORFs with red color represent free-standing ORFs.

Table S3 The composition of 7 *Moelleriella* mitogenomes

Features	*Moelleriella zhongdongii*	*Moelleriella libera*	*Moelleriella raciborskii*	*Moelleriella gracilispora*	*Moelleriella oxystoma*	*Moelleriella* sp.C9	*Moelleriella* sp.C3
Length of PCGs (bp)	20241	17739	15705	24654	24873	20241	19434
Length of RNA regions (bp)	5931	6490	6337	6433	6483	6388	6394
Length of intergenic regions (bp)	9591	4521	9056	8102	15889	7381	9780
Length of intronic regions (bp)	21260	12078	14373	40367	48421	19819	19288
Length of mitogenomes (bp)	57023	40828	45471	79556	95666	53829	54896
Length of PCGs (%)	35.50%	43.45%	34.54%	30.99%	26.00%	37.60%	35.40%
Length of RNA regions (%)	11.11%	15.90%	13.94%	8.09%	6.78%	11.87%	11.65%
Length of intergenic regions (%)	37.28%	11.07%	31.61%	10.18%	50.61%	36.82%	35.14%
Length of intronic regions (%)	16.11%	29.58%	19.92%	50.74%	16.61%	13.71%	17.82%

Table S4 Start and stop codon analyses of 15 core protein-coding genes in 27 Hypocreales species

Species	*atp6*		*atp8*		*atp9*		*cob*		*cox1*		*cox2*		*cox3*		*nad1*		*nad2*		*nad3*		*nad4*		*nad4L*		*nad5*		*nad6*		*rps3*	
	start codon	stop codon	start codon	stop codon	start codon	stop codon	start codon	stop codon	start codon	stop codon	start codon	stop codon	start codon	stop codon	start codon	stop codon	start codon	stop codon	start codon	stop codon	start codon	stop codon	start codon	stop codon	start codon	stop codon	start codon	stop codon	start codon	stop codon
Moelleriella.zhongdongii	ATG	TAA	ATG	TAA	ATG	TAG	ATG	TAA	ATG	TAA	ATG	TAA	ATG	TAA	ATG	TAA	ATG	TAA	TTG	TAA	ATG	TAA	ATG	TAA	ATG	TAA	ATG	TAA	ATG	TAA
Moelleriella libera	ATG	TAA	ATG	TAA	ATG	TAA	ATG	TAA	ATG	TAA	ATG	TAA	ATG	TAA	ATG	TAA	ATG	TAA	ATG	TAA	ATG	TAA	ATG	TAA	ATG	TAA	ATG	TAA	ATG	TAG
Moelleriella raciborskii	ATG	TAA	ATG	TAA	ATG	TAA	ATG	TAA	ATG	TAA	ATG	TAA	ATG	TAA	ATG	TAA	ATG	TAA	ATG	TAA	ATG	TAA	ATG	TAA	ATG	TAA	ATG	TAA	ATG	TAA
Moelleriella gracilispora	ATG	TAA	ATG	TAA	ATG	TAA	ATG	TAA	ATG	TAA	ATG	TAA	ATG	TAA	ATG	TAA	ATG	TAA	ATG	TAA	ATG	TAA	ATG	TAA	ATG	TAA	ATG	TAA	ATG	TAA
Moelleriella oxystoma	ATG	TAA	ATG	TAA	ATG	TAA	ATG	TAA	ATG	TAA	ATG	TAA	ATG	TAA	ATG	TAA	ATG	TAA	TTG	TAA	ATG	TAA	ATG	TAA	ATG	TAA	ATG	TAA	ATG	TAA
Moelleriella sp.C9	ATG	TAA	ATG	TAA	ATG	TAA	ATG	TAA	ATG	TAA	ATG	TAA	ATG	TAA	ATG	TAG	ATG	TAA	TTG	TAA	ATG	TAA	ATG	TAA	ATG	TAA	ATG	TAA	ATG	TAA
Moelleriella sp.C3	ATG	TAA	ATG	TAA	ATG	TAA	ATG	TAA	ATG	TAA	TTG	TAA	ATG	TAA	ATG	TAG	ATG	TAA	ATG	TAA	ATG	TAA	ATG	TAA	ATG	TAA	ATG	TAA	ATG	TAA
Metarhizium album	ATG	TAA	ATG	TAA	ATG	TAA	ATG	TAA	ATG	TAA	ATG	TAA	ATG	TAA	ATG	TAA	ATG	TAA	ATG	TAA	ATG	TAA	ATG	TAA	ATG	TAA	ATG	TAA	ATG	TAA
Beauveria.pseudobassiana	ATG	TAA	ATG	TAA	ATG	TAA	ATG	TAA	ATG	TAA	ATG	TAA	ATG	TAA	ATG	TAA	ATG	TAA	ATG	TAA	ATG	TAA	ATG	TAA	ATG	TAA	ATG	TAA	ATG	TAA
Cordyceps militaris	ATG	TAA	ATG	TAA	ATG	TAA	ATG	TAA	ATG	TAA	ATG	TAA	ATG	TAA	ATG	TAA	ATG	TAA	ATG	TAA	ATG	TAA	ATG	TAA	ATG	TAA	ATG	TAA	ATG	TAA
Akanthomyces lecanii	ATG	TAA	ATG	TAA	ATG	TAA	ATG	TAA	TTG	TAA	ATG	TAA	ATG	TAA	ATG	TAA	ATG	TAA	ATG	TAA	ATG	TAA	ATG	TAA	ATG	TAA	ATG	TAA	ATG	TAA
Clonostachys byssicola	ATG	TAA	ATG	TAA	ATG	TAG	ATG	TAA	ATG	TAA	ATG	TAA	ATG	TAA	ATG	TAA	ATG	TAA	ATG	TAA	ATG	TAA	ATG	TAA	ATG	TAA	ATG	TAA	ATG	TAA
Purpureocillium takamizusanense	ATG	TAA	ATG	TAA	ATG	TAA	ATG	TAA	TTG	TAA	ATG	TAA	ATG	TAA	ATG	TAA	ATG	TAA	ATG	TAA	ATG	TAA	ATG	TAA	ATG	TAA	ATG	TAA	ATG	TAA
Fusarium ussurianum	ATG	TAA	ATG	TAA	ATG	TAA	ATG	TAG	ATG	TAA	ATG	TAA	ATG	TAA	ATG	TAG	ATG	TAA	ATG	TAG	ATG	TAA	ATG	TAA	ATG	TAG	ATG	TAG	ATG	TAA
Trichoderma afroharzianum	ATG	TAA	ATG	TAA	ATG	TAA	ATG	TAA	ATG	TAA	ATG	TAA	ATG	TAA	ATG	TAA	ATG	TAA	ATG	TAA	ATG	TAA	ATG	TAA	ATG	TAA	ATG	TAA	ATG	TAA
Stachybotrys chlorohalonata	ATG	TAA	ATG	TAA	ATG	TAG	GTG	TAA	ATG	TAA	ATG	TAA	TTG	TAA	ATG	TAA	ATG	TAA	ATG	TAA	ATG	TAA	ATG	TAA	ATG	TAA	ATG	TAA	ATG	TAA
Stachybotrys chartarum	ATG	TAA	ATG	TAA	ATG	TAG	GTG	TAA	ATG	TAA	ATG	TAA	TTG	TAA	ATG	TAA	ATG	TAA	ATG	TAA	ATG	TAA	ATG	TAA	ATG	TAG	ATG	TAA	ATG	TAA
Zelopaecilomyces penicillatus	ATG	TAA	ATG	TAA	ATG	TAA	ATG	TAA	ATG	TAA	ATG	TAA	ATG	TAA	ATG	TAA	ATG	TAA	ATG	TAA	ATG	TAA	ATG	TAA	ATG	TAA	ATG	TAA	ATG	TAA
Tolypocladium cylindrosporum	ATG	TAA	ATG	TAA	ATG	TAG	ATG	TAA	ATG	TAA	ATG	TAA	ATG	TAA	ATG	TAA	ATG	TAA	ATG	TAA	ATG	TAA	ATG	TAA	ATG	TAA	ATG	TAA	ATG	TAA
Samsoniella hepiali	ATG	TAA	ATG	TAA	ATG	TAA	ATG	TAA	TTG	TAA	ATG	TAA	ATG	TAA	ATG	TAA	ATG	TAA	ATG	TAA	ATG	TAA	ATG	TAA	ATG	TAA	ATG	TAA	ATG	TAA
Pleurocordyceps sinensis	ATG	TAA	ATG	TAA	ATG	TAA	ATG	TAA	ATA	TAA	ATG	TAA	ATG	TAA	ATG	TAA	ATG	TAA	ATG	TAA	ATG	TAA	ATG	TAA	ATG	TAA	ATG	TAA	ATG	TAA
Parengyodontium album	ATG	TAA	ATG	TAA	ATG	TAA	ATG	TAA	ATG	TAA	ATG	TAA	ATG	TAA	ATG	TAA	ATG	TAA	ATG	TAA	ATG	TAA	ATG	TAA	ATG	TAA	ATG	TAA	ATG	TAA
Orbiocrella petchii	ATG	TAA	ATG	TAA	ATG	TAA	ATG	TAA	ATG	TAA	ATG	TAA	ATG	TAA	ATG	TAA	ATG	TAA	ATG	TAA	ATG	TAA	ATG	TAA	ATG	TAA	ATG	TAA	ATG	TAA
Nectria cinnabarina	ATG	TAA	ATG	TAA	ATG	TAA	ATG	TAA	ATG	TAA	ATG	TAA	ATG	TAA	ATG	TAA	ATG	TAA	ATG	TAA	ATG	TAA	ATG	TAA	ATG	TAA	ATG	TAA	ATG	TAA
Metacordyceps chlamydosporia	ATG	TAA	ATG	TAA	ATG	TAA	ATG	TAA	ATG	TAA	ATG	TAA	ATG	TAA	ATG	TAA	ATG	TAA	ATG	TAA	ATG	TAA	ATG	TAA	ATG	TAG	ATG	TAA	ATG	TAA
Memnoniella echinata	ATG	TAA	ATG	TAA	ATG	TAA	ATG	TAG	ATG	TAA	ATG	TAA	TTG	TAA	ATG	TAA	ATG	TAA	ATG	TAA	ATG	TAA	ATG	TAA	ATG	TAG	ATG	TAA	ATG	TAA
Gibberella moniliformis	ATG	TAA	ATG	TAA	ATG	TAA	ATG	TAA	ATG	TAA	ATG	TAA	ATG	TAA	ATG	TAG	ATG	TAA	ATG	TAG	ATG	TAA	ATG	TAA	ATG	TAG	ATG	TAA	ATG	TAA

Table S5 Codon usage analysis of 27 Hypocreales species

		M.zhongdongii	*M.libera*	*M.raciborskii*	*.gracilispor*	*M.oxystoma*	*M.sp.C9*	*M.sp.C3*	*M.album*	*homyces*	*leria.psedobo*	*stachys.bys*	*dyceps militum*	*m.ussu*	*erella.monilifa*	*noniella.echi*	*ceps.chlan*	*ria.cinnaba*	*iocrella.pe*	*yodontium*	*ordyceps.s*	*lium.takan*	*soniella he*	*botrys.cha*	*trys.chloro*	*dium.cylinc*	*rma.afroh*	*lomyces.penicillatus*
AA	Codon	RSCU	RSCU	RSCU	RSCU	RSCU	RSCU	RSCU	RSCU	RSCU	RSCU	RSCU	RSCU	RSCU	RSCU	RSCU	RSCU	RSCU	RSCU	RSCU	RSCU	RSCU	RSCU	RSCU	RSCU	RSCU	RSCU	RSCU
TTT	Phe	1.48	1.49	1.45	1.55	1.5	1.55	1.54	1.52	1.5	1.51	1.47	1.44	1.43	1.53	1.6	1.44	1.48	1.53	1.58	1.67	1.55	1.44	1.59	1.58	1.55	1.44	1.39
TTC	Phe	0.52	0.51	0.55	0.45	0.5	0.45	0.46	0.48	0.5	0.49	0.53	0.56	0.57	0.47	0.4	0.56	0.52	0.47	0.42	0.33	0.45	0.56	0.41	0.42	0.45	0.56	0.61
TTA	Leu	2.66	2.78	2.9	2.72	2.49	2.59	2.76	2.92	2.93	2.5	2.96	3.06	2.24	2.07	3.37	2.59	2.85	1.97	2.85	2.62	2.84	3.82	2.62	3.12	2.44	2.88	2.37
TTG	Leu	0.81	0.78	0.74	0.76	0.69	0.78	0.75	0.71	0.64	0.77	0.69	0.76	0.71	0.71	0.69	0.77	0.66	0.85	0.78	0.66	0.8	0.58	0.79	0.66	0.77	0.68	0.77
CTT	Leu	0.9	0.86	0.82	0.96	1.05	0.96	0.88	0.93	0.86	0.82	0.87	0.78	1.04	1.21	0.81	0.89	0.88	1.09	0.85	1.07	1.01	0.71	0.95	0.89	1.18	0.92	1.14
CTC	Leu	0.24	0.27	0.24	0.23	0.33	0.24	0.22	0.23	0.27	0.23	0.22	0.29	0.44	0.39	0.2	0.3	0.3	0.33	0.27	0.33	0.18	0.15	0.29	0.16	0.34	0.27	0.34
CTA	Leu	0.97	0.93	0.94	0.96	1.04	1.02	1.02	0.9	0.96	1.22	0.93	0.85	1.1	1.17	0.62	1.01	0.97	1.19	0.95	0.95	0.8	0.59	0.95	0.88	0.95	0.92	1.04
CTG	Leu	0.43	0.38	0.37	0.37	0.4	0.41	0.36	0.31	0.34	0.45	0.33	0.25	0.47	0.45	0.31	0.44	0.34	0.57	0.3	0.38	0.36	0.16	0.39	0.29	0.34	0.33	0.35
TCT	Ser	1.36	1.31	1.3	1.2	1.36	1.24	1.33	1.54	1.18	1.2	1.41	1.07	1.33	1.1	1.3	1.35	1.31	1.77	1.54	1.53	1.38	1.12	1.34	1.53	1.23	1.18	1.2
TCC	Ser	0.39	0.6	0.37	0.48	0.61	0.45	0.4	0.45	0.47	0.55	0.45	0.46	0.61	0.53	0.42	0.59	0.45	0.5	0.41	0.45	0.51	0.39	0.52	0.46	0.53	0.49	0.56
TCA	Ser	1.32	1.04	1.13	1.26	1.11	1.16	1.22	1.16	1.12	0.99	1.08	1.12	0.93	0.81	1.21	1.02	1.06	0.97	1.2	1.13	1.12	1.25	1.26	1.18	1.05	1.19	1.14
TCG	Ser	0.47	0.37	0.36	0.3	0.41	0.33	0.31	0.3	0.36	0.5	0.35	0.42	0.53	0.51	0.31	0.62	0.36	0.81	0.4	0.28	0.34	0.4	0.42	0.28	0.42	0.34	0.32
AGT	Ser	1.76	1.86	1.92	1.85	1.66	1.82	1.84	1.82	1.7	1.51	1.73	1.81	1.54	1.45	2	1.67	1.92	1.17	1.81	1.59	1.89	1.96	1.49	1.92	1.79	1.83	1.67
AGC	Ser	0.69	0.82	0.92	0.9	0.86	1.01	0.9	0.73	1.16	1.25	0.98	1.12	1.06	1.62	0.77	0.75	0.9	0.78	0.65	1.02	0.75	0.88	0.97	0.64	0.99	0.96	1.1
TAT	Tyr	1.52	1.44	1.49	1.55	1.47	1.49	1.51	1.55	1.58	1.59	1.52	1.57	1.47	1.56	1.62	1.57	1.51	1.55	1.62	1.58	1.54	1.63	1.5	1.55	1.52	1.57	1.55
TAC	Tyr	0.48	0.56	0.51	0.45	0.53	0.51	0.49	0.45	0.42	0.41	0.48	0.43	0.53	0.44	0.38	0.43	0.49	0.45	0.38	0.42	0.46	0.37	0.5	0.45	0.48	0.43	0.45
TGT	Cys	1.35	1.4	1.29	1.29	1.37	1.24	1.27	1.4	1.13	1.26	1.28	1.18	1.21	1.02	1.3	1.36	1.27	1.29	1.39	1.28	1.35	1.25	1.33	1.37	1.23	1.59	1.22
TGC	Cys	0.65	0.6	0.71	0.71	0.63	0.76	0.73	0.6	0.87	0.74	0.72	0.82	0.79	0.98	0.7	0.64	0.73	0.71	0.61	0.72	0.65	0.75	0.67	0.63	0.77	1.82	0.78
TGG	Trp	1.91	1.83	1.85	1.87	1.82	1.8	1.53	2.24	1.76	1.86	1.95	1.74	1.68	1.77	2.13	1.86	1.83	1.35	2.41	1.97	2	2.03	1.82	1.73	1.88	2.12	1.95
CCT	Pro	0.48	0.61	0.57	0.58	0.72	0.59	0.47	0.44	0.62	0.72	0.48	0.47	0.82	0.7	0.57	5	0.7	0.6	0.31	0.39	0.44	0.38	0.55	0.59	0.45	0.46	0.4
CCC	Pro	1.12	1.12	1.11	1.13	1.02	1.17	1.03	1	1.29	0.85	1.21	1.31	0.95	1.02	0.92	1.26	0.99	1.35	0.98	1.16	0.97	1.33	1.14	1.24	1.07	1.16	1.24
CCA	Pro	0.49	0.43	0.47	0.41	0.44	0.44	0.5	0.32	0.32	0.57	0.36	0.47	0.55	0.51	0.38	0.39	0.48	0.7	0.31	0.49	0.59	0.25	0.49	0.45	0.6	0.26	0.4
CCG	Pro	1.4	1.44	1.32	1.4	1.44	1.45	1.45	1.55	1.57	1.5	1.35	1.6	1.35	1.29	1.5	1.45	1.42	1.51	1.46	1.51	1.43	1.64	1.41	1.44	1.39	1.29	1.41
CAT	His	0.6	0.56	0.68	0.6	0.56	0.55	0.55	0.45	0.43	0.5	0.65	0.4	0.65	0.71	0.5	0.55	0.58	0.49	0.54	0.49	0.57	0.36	0.59	0.56	0.61	0.71	0.59
CAC	His	1.33	1.29	1.45	1.42	1.28	1.3	1.33	1.43	1.18	1.15	1.3	1.35	1.24	1.14	1.5	1.26	1.32	1.05	1.3	1.35	1.35	1.47	1.45	1.51	1.24	1.33	1.3
CAA	Gln	0.67	0.71	0.55	0.58	0.72	0.7	0.67	0.57	0.82	0.85	0.7	0.65	0.76	0.86	0.5	0.74	0.68	0.95	0.7	0.65	0.65	0.53	0.55	0.49	0.76	0.67	0.7
CAG	Gln	0.62	0.67	0.54	0.48	0.42	0.58	0.62	0.51	0.52	0.37	0.63	0.39	0.6	0.52	0.52	0.74	0.6	0.79	0.52	0.49	0.57	0.44	0.43	0.61	0.52	0.7	0.28
CGT	Arg	0.26	0.31	0.35	0.19	0.26	0.29	0.19	0.19	0.23	0.44	0.35	0.24	0.45	0.57	0.13	0.35	0.33	0.19	0.29	0.2	0.14	0.17	0.16	0.22	0.22	0.3	0.3
CGC	Arg	0.53	0.44	0.47	0.42	0.44	0.45	0.46	0.37	0.68	0.62	0.54	0.49	0.69	0.62	0.53	0.52	0.53	0.64	0.55	0.49	0.46	0.6	0.47	0.63	0.51	0.54	0.57
CGA	Arg	0.48	0.44	0.41	0.35	0.41	0.28	0.3	0.35	0.4	0.42	0.52	0.36	0.5	0.49	0.4	0.47	0.53	0.45	0.42	0.37	0.28	0.27	0.37	0.49	0.32	0.34	0.37
CGG	Arg	2.65	2.68	2.66	3.03	2.97	2.74	2.91	2.95	2.34	2.33	2.79	2.38	2.35	2.03	2.77	2.15	2.62	2.02	2.89	2.43	2.96	2.61	2.7	2.53	2.75	2.42	2.54
AGA	Arg	1.47	1.46	1.56	1.53	1.51	1.68	1.52	1.63	1.84	1.82	1.27	2.14	1.41	1.77	1.65	1.77	1.39	1.91	1.33	2.02	1.6	1.91	1.88	1.52	1.67	1.71	1.94
AGG	Arg	1.18	1.21	1.19	1.3	1.27	1.31	1.31	1.34	1.38	1.25	1.24	1.36	1.16	1.31	1.38	1.21	1.19	1.35	1.15	1.41	1.28	1.41	1.39	1.2	1.37	1.23	1.4
ATT	Ile	0.34	0.33	0.31	0.36	0.41	0.35	0.33	0.36	0.37	0.4	0.34	0.36	0.44	0.34	0.24	0.39	0.36	0.32	0.34	0.3	0.3	0.28	0.37	0.31	0.4	0.39	0.39
ATC	Ile	1.48	1.46	1.5	1.29	1.32	1.34	1.36	1.3	1.25	1.35	1.42	1.28	1.4	1.35	1.38	1.4	1.45	1.33	1.51	1.28	1.42	1.31	1.24	1.49	1.23	1.38	1.21
ATA	Ile	1.56	1.33	1.33	1.39	1.35	1.4	1.3	1.44	1.17	1.15	1.45	1.19	1.45	1.44	1.46	1.38	1.47	1.28	1.44	1.42	1.65	1.36	1.37	1.38	1.23	1.43	1.05
ATG	Met	0.55	0.58	0.57	0.68	0.75	0.73	0.63	0.58	0.7	0.7	0.43	0.69	0.61	0.7	0.64	0.67	0.53	0.73	0.5	0.56	0.61	0.6	0.88	0.51	0.78	0.49	0.71
ACT	Thr	1.44	1.53	1.64	1.56	1.45	1.4	1.64	1.59	1.73	1.73	1.57	1.64	1.39	1.31	1.54	1.44	1.58	1.25	1.58	1.64	1.41	1.69	1.29	1.62	1.52	1.64	1.78
ACC	Thr	0.45	0.56	0.47	0.37	0.45	0.47	0.42	0.4	0.4	0.42	0.55	0.48	0.54	0.55	0.36	0.51	0.42	0.75	0.49	0.39	0.33	0.35	0.46	0.49	0.47	0.44	0.47
ACA	Thr	1.46	1.43	1.45	1.47	1.43	1.5	1.46	1.58	1.52	1.47	1.49	1.49	1.51	1.54	1.58	1.52	1.47	1.54	1.66	1.5	1.51	1.62	1.49	1.49	1.47	1.5	1.51

ACG	Thr	0.54	0.57	0.55	0.53	0.57	0.5	0.54	0.42	0.48	0.53	0.51	0.51	0.49	0.46	0.42	0.48	0.53	0.46	0.34	0.5	0.49	0.38	0.51	0.51	0.53	0.5	0.49
AAT	Asn	1.45	1.48	1.38	1.43	1.46	1.42	1.46	1.45	1.3	1.29	1.41	1.29	1.37	1.31	1.46	1.29	1.42	1.28	1.46	1.43	1.46	1.4	1.38	1.46	1.35	1.43	1.41
AAC	Asn	0.55	0.52	0.62	0.57	0.54	0.58	0.54	0.55	0.7	0.71	0.59	0.71	0.63	0.69	0.54	0.71	0.58	0.72	0.54	0.57	0.54	0.6	0.62	0.54	0.65	0.57	0.59
AAA	Lys	1.41	1.31	1.41	1.46	1.39	1.37	1.39	1.49	1.36	1.31	1.3	1.35	1.31	1.36	1.48	1.26	1.31	1.33	1.38	1.4	1.35	1.51	1.56	1.46	1.5	1.3	1.35
AAG	Lys	0.35	0.37	0.35	0.37	0.44	0.38	0.39	0.29	0.4	0.43	0.3	0.37	0.43	0.37	0.34	0.34	0.37	0.47	0.34	0.34	0.38	0.36	0.35	0.32	0.41	0.25	0.39
GTT	Val	1.6	1.65	1.7	1.65	1.63	1.7	1.65	1.71	1.62	1.53	1.76	1.62	1.6	1.51	1.74	1.67	1.76	1.4	1.61	1.7	1.74	1.68	1.41	1.53	1.45	1.76	1.62
GTC	Val	0.65	0.67	0.54	0.51	0.54	0.54	0.58	0.51	0.62	0.72	0.63	0.67	0.66	0.76	0.45	0.73	0.56	0.79	0.66	0.56	0.53	0.44	0.68	0.68	0.64	0.68	0.63
GTA	Val	1.79	1.73	1.93	1.73	1.63	1.74	1.77	1.9	1.74	1.74	1.89	2	1.9	1.94	2.06	1.65	1.79	1.38	2.08	2.2	1.77	2.09	1.61	2.04	1.91	1.92	1.8
GTG	Val	0.49	0.76	0.6	0.55	0.67	0.66	0.5	0.52	0.48	0.61	0.57	0.46	0.46	0.62	0.44	0.54	0.68	0.61	0.35	0.46	0.48	0.3	0.54	0.4	0.58	0.48	0.53
GCT	Ala	1.31	1.1	1.09	1.36	1.26	1.32	1.31	1.18	1.49	1.29	1.18	1.23	1.03	0.94	1.26	1.42	1.11	1.28	1.23	1	1.44	1.33	1.45	1.22	1.17	1.3	1.29
GCC	Ala	0.42	0.41	0.38	0.36	0.45	0.29	0.42	0.4	0.28	0.36	0.36	0.31	0.61	0.49	0.23	0.38	0.42	0.74	0.33	0.34	0.31	0.28	0.4	0.33	0.34	0.3	0.38
GCA	Ala	1.51	1.55	1.47	1.5	1.53	1.55	1.54	1.58	1.53	1.5	1.47	1.53	1.56	1.47	1.6	1.56	1.54	1.58	1.69	1.6	1.63	1.6	1.45	1.51	1.58	1.51	1.46
GCG	Ala	0.49	0.45	0.53	0.5	0.47	0.45	0.46	0.42	0.47	0.5	0.53	0.47	0.44	0.53	0.4	0.44	0.46	0.42	0.31	0.4	0.37	0.4	0.55	0.49	0.42	0.49	0.54
GAT	Asp	1.42	1.4	1.38	1.48	1.37	1.47	1.43	1.52	1.42	1.43	1.45	1.36	1.3	1.17	1.56	1.33	1.38	1.34	1.44	1.46	1.48	1.57	1.41	1.49	1.37	1.54	1.4
GAC	Asp	0.58	0.6	0.62	0.52	0.63	0.53	0.57	0.48	0.58	0.57	0.55	0.64	0.7	0.83	0.44	0.67	0.62	0.66	0.56	0.54	0.52	0.43	0.59	0.51	0.63	0.46	0.6
GAA	Glu	1.52	1.52	1.64	1.59	1.39	1.67	1.67	1.63	1.8	1.55	1.61	1.64	1.28	1.02	1.81	1.44	1.53	1.65	1.84	1.63	1.6	1.85	1.62	1.85	1.3	1.73	1.45
GAG	Glu	0.37	0.44	0.35	0.39	0.45	0.47	0.36	0.41	0.41	0.65	0.46	0.52	0.54	0.75	0.26	0.6	0.55	0.47	0.37	0.37	0.43	0.32	0.45	0.32	0.63	0.31	0.48
GGT	Gly	1.4	1.34	1.43	1.43	1.2	1.29	1.32	1.39	1.18	1.09	1.38	1.09	1.28	1.24	1.46	1.46	1.25	1.15	1.4	1.31	1.38	1.36	1.3	1.41	1.5	1.5	1.36
GGC	Gly	0.71	0.7	0.59	0.59	0.96	0.57	0.66	0.58	0.62	0.71	0.55	0.75	0.89	0.99	0.47	0.5	0.67	0.74	0.4	0.7	0.6	0.48	0.63	0.42	0.57	0.46	0.71
GGA	Gly	1	1	1	1	1	1	1	1	1	1	1	1	1	1	1	1	1	1	1	1	1	1	1	1	1	1	1
GGG	Gly	1	1	1	1	1	1	1	1	1	1	1	1	1	1	1	1	1	1	1	1	1	1	1	1	1	1	1

Table S6 Repetitive sequence analysis of 7 *Moelleriella* species

Query/Subject ID	Identity(%)	Aligment length	Mismatches	Gap openings	Q.start	Q.end	S.start	S.end	e-value	Bit score
Moelleriella.zhongdongii	83.673	196	28	2	54939	55132	13908	14101	4.97E-46	182
Moelleriella.zhongdongii	83.673	196	28	2	13908	14101	54939	55132	4.97E-46	182
Moelleriella.zhongdongii	100	96	0	0	56928	57023	1	96	6.43E-45	178
Moelleriella.zhongdongii	100	96	0	0	1	96	56928	57023	6.43E-45	178
Moelleriella. gracilispora	87.018	285	27	7	38594	38869	37188	37471	3.29E-85	313
Moelleriella. gracilispora	87.018	285	27	7	37188	37471	38594	38869	3.29E-85	313
Moelleriella. gracilispora	100	127	0	0	79430	79556	1	127	7.33E-62	235
Moelleriella. gracilispora	100	127	0	0	1	127	79430	79556	7.33E-62	235
Moelleriella. gracilispora	80.321	249	37	6	63509	63746	14930	15177	1.25E-44	178
Moelleriella. gracilispora	80.321	249	37	6	14930	15177	63509	63746	1.25E-44	178
Moelleriella. gracilispora	80.097	206	27	9	63509	63701	37211	37415	1.64E-33	122
Moelleriella. gracilispora	80.097	206	27	9	37211	37415	63509	63701	1.64E-33	122
Moelleriella. gracilispora	76.027	292	48	19	60637	60921	27894	28170	9.89E-31	132
Moelleriella. gracilispora	76.027	292	48	19	27894	28170	60637	60921	9.89E-31	132
Moelleriella. gracilispora	90	90	7	2	10159	10247	3138	3226	9.96E-26	115
Moelleriella. gracilispora	90	90	7	2	3138	3226	10159	10247	9.96E-26	115
Moelleriella. gracilispora	87.805	82	5	3	71638	71718	30832	30909	1.68E-18	91.6
Moelleriella. gracilispora	87.805	82	5	3	30832	30909	71638	71718	1.68E-18	91.6
Moelleriella. gracilispora	86.842	76	9	1	46421	46495	29905	29980	2.81E-16	84.2
Moelleriella. gracilispora	86.842	76	9	1	29905	29980	46421	46495	2.81E-16	84.2
Moelleriella.libera	100	65	0	0	39850	39914	6	70	5.64E-28	121
Moelleriella.libera	100	65	0	0	6	70	39850	39914	5.64E-28	121
Moelleriella sp.C9	88.199	161	14	4	1411	1566	5	165	9.52E-48	187
Moelleriella sp.C9	88.199	161	14	4	5	165	1411	1566	9.52E-48	187
Moelleriella sp.C9	88.136	118	13	1	53711	53827	1287	1404	2.70E-33	139
Moelleriella sp.C9	88.136	118	13	1	1287	1404	53711	53827	2.70E-33	139
Moelleriella sp.C9	85.149	101	5	7	36439	36533	19351	19447	5.94E-20	95.3
Moelleriella sp.C9	85.149	101	5	7	19351	19447	36439	36533	5.94E-20	95.3
Moelleriella sp.C9	83.544	79	11	2	36439	36533	19351	19447	2.78E-13	73.1
Moelleriella sp.C9	83.544	79	11	2	19351	19447	36439	36533	2.78E-13	73.1
Moelleriella sp.C9	89.091	55	2	3	46871	46924	45921	45972	4.66E-11	65.8
Moelleriella sp.C9	89.091	55	2	3	45921	45972	46871	46924	4.66E-11	65.8
Moelleriella sp.C3	85.149	101	5	7	26002	26096	12133	12229	6.18E-20	95.3
Moelleriella sp.C3	85.149	101	5	7	12133	12229	26002	26096	6.18E-20	95.3
Moelleriella oxystoma	100	127	0	0	95540	95666	1	127	1.06E-61	235
Moelleriella oxystoma	100	127	0	0	1	127	95540	95666	1.06E-61	235
Moelleriella oxystoma	75.721	416	79	16	27026	27431	23890	24293	8.37E-48	189
Moelleriella oxystoma	75.721	416	79	16	23890	24293	27026	27431	8.37E-48	189
Moelleriella oxystoma	77.489	231	43	7	34126	34351	23900	24126	5.14E-30	130
Moelleriella oxystoma	77.489	231	43	7	23900	24126	34126	34351	5.14E-30	130
Moelleriella oxystoma	92.045	88	7	0	77792	77879	25418	25505	2.39E-28	124
Moelleriella oxystoma	92.045	88	7	0	25418	25505	77792	77879	2.39E-28	124
Moelleriella oxystoma	75.732	239	47	9	77683	77915	3534	3767	6.70E-24	110

Moelleriella oxystoma	75.833	240	45	11	3534	3767	77683	77915	6.70E-24	110
Moelleriella oxystoma	75.463	216	41	10	25381	25594	23963	24168	1.88E-19	95.3
Moelleriella oxystoma	75.576	217	39	12	23963	24168	25381	25594	1.88E-19	95.3
Moelleriella oxystoma	79.365	126	14	9	34238	34353	25431	25554	1.89E-14	78.7
Moelleriella oxystoma	79.528	127	12	10	25431	25554	34238	34353	1.89E-14	78.7
Moelleriella oxystoma	100	36	0	0	83155	83190	83190	83155	4.09E-11	67.6
Moelleriella raciborskii	82.759	87	15	0	11383	11469	11341	11427	4.27E-15	78.7
Moelleriella raciborskii	82.759	87	15	0	11341	11427	11383	11469	4.27E-15	78.7

Table S7 Tandem repeats detected in seven *Moelleriella* mitogenomes using the online program Tandem Repeats Finder

Indices	Period Size	Copy Number	Consensus Size	Percent Matches	Percent Indels	Score	A	C	G	T	Entropy (0-2)
Moelleriella oxystoma											
6823--6861	14	2.8	14	84	0	50	71	2	0	25	0.98
43989--44045	24	2.4	24	78	0	72	43	5	29	21	1.74
44025--44115	33	2.8	33	81	0	112	30	5	39	24	1.78
44056--44112	12	5	12	70	12	65	24	7	40	28	1.81
44056--44114	21	2.7	21	76	7	70	25	6	40	27	1.8
44602--44640	15	2.6	15	75	0	50	2	41	7	48	1.45
46421--46465	22	2.2	20	84	8	58	22	4	8	64	1.4
65916--65978	33	2	33	81	6	84	52	6	9	31	1.59
80309--80356	21	2.2	22	77	3	54	31	6	12	50	1.65
Moelleriella gracilispora											
Indices	Period Size	Copy Number	Consensus Size	Percent Matches	Percent Indels	Score	A	C	G	T	Entropy (0-2)
2077--2126	24	2.1	24	100	0	100	50	8	8	34	1.61
5236--5279	18	2.5	18	85	10	58	43	4	2	50	1.35
12346--12382	18	2	19	84	5	53	51	2	8	37	1.46
20227--20274	15	3.3	15	77	11	52	37	10	6	45	1.64
29076--29115	19	2.1	19	80	0	52	52	12	2	32	1.52
34184--34236	24	2.2	24	75	0	64	64	1	1	32	1.15
36177--36220	21	2.1	21	78	0	53	45	4	13	36	1.64
45373--45425	12	4.3	12	75	11	53	73	1	1	22	1.03
45384--45425	22	2	21	80	4	54	73	2	2	21	1.06
50365--50412	21	2.2	23	81	7	61	60	4	10	25	1.47
52873--52923	26	2	26	80	7	65	29	5	9	54	1.56
69439--69480	20	2	21	81	4	56	38	9	7	45	1.64
76235--76266	15	2.1	15	94	5	55	34	0	0	65	0.93
76386--76425	21	2	20	85	5	57	15	2	10	72	1.21
***Moelleriella* sp.C3**											
Indices	Period Size	Copy Number	Consensus Size	Percent Matches	Percent Indels	Score	A	C	G	T	Entropy (0-2)
1347--1404	21	2.6	22	78	8	72	60	3	8	27	1.42
4120--4192	19	4.1	18	63	13	51	28	4	8	58	1.45
10442--10496	28	2	28	85	3	82	56	1	5	36	1.33
15332--15373	20	2	21	81	4	56	38	9	7	45	1.64
28255--28305	22	2.4	22	76	3	60	39	11	7	41	1.71
35995--36042	15	3.3	15	77	11	52	37	10	6	45	1.64
41001--41041	22	1.9	22	85	5	61	73	0	0	26	0.84
46238--46281	21	2.1	21	78	0	53	45	4	13	36	1.64
52487--52528	22	2	21	80	4	54	73	4	2	19	1.12
***Moelleriella* sp.C9**											
Indices	Period Size	Copy Number	Consensus Size	Percent Matches	Percent Indels	Score	A	C	G	T	Entropy (0-2)
4882--4934	12	4.3	12	75	11	53	73	1	1	22	1.03
4893--4934	22	2	21	80	4	54	73	2	2	21	1.06

9163--9207	20	2.2	21	76	8	55	62	8	0	28	1.25
13548--13589	22	1.9	22	80	0	56	35	4	0	59	1.19
25201--25242	20	2	21	81	4	56	38	9	7	45	1.64
29640--29678	21	1.9	20	84	5	55	20	12	23	43	1.86
45709--45756	15	3.3	15	77	11	52	37	10	6	45	1.64
47704--47754	23	2.3	23	79	3	60	35	7	3	52	1.49
52708--52751	21	2.1	21	78	0	53	45	4	13	36	1.64

Moelleriella raciborskii

Indices	Period Size	Copy Number	Consensus Size	Percent Matches	Percent Indels	Score	A	C	G	T	Entropy (0-2)
5044--5087	20	2.2	20	91	0	74	38	18	20	22	1.93
11335--11471	21	6.5	21	81	0	176	37	28	7	27	1.83
11335--11469	63	2.1	63	86	0	200	37	28	6	27	1.82
11891--11956	33	2	33	81	0	90	45	21	12	21	1.84
11997--12079	36	2.3	36	93	0	145	9	34	14	40	1.79
13126--13167	20	2	21	81	4	56	38	11	7	42	1.69
30931--30970	19	2.1	19	80	0	52	52	12	2	32	1.52
32246--32286	21	2	21	80	0	54	21	17	36	24	1.94
36316--36359	15	2.9	15	80	10	58	29	0	9	61	1.27
37704--37764	25	2.6	22	70	14	58	47	6	4	40	1.51
38502--38537	17	2.1	17	84	0	51	58	11	19	11	1.62
42216--42248	14	2.3	15	89	5	52	75	6	0	18	1
43753--43783	14	2.3	13	94	5	53	51	0	3	45	1.17

Moelleriella libera

Indices	Period Size	Copy Number	Consensus Size	Percent Matches	Percent Indels	Score	A	C	G	T	Entropy (0-2)
8233--8285	21	2.5	20	76	8	60	35	7	1	54	1.4
11214--11242	13	2.2	13	93	0	51	44	6	6	41	1.58
12367--12402	17	2.1	17	84	0	51	58	11	19	11	1.62
15224--15256	14	2.3	15	89	5	52	75	6	0	18	1
17851--17883	17	1.9	17	87	0	52	39	3	15	42	1.62
26069--26181	21	5.4	21	75	0	121	38	26	9	25	1.87
26592--26659	33	2.1	33	82	0	94	47	17	14	20	1.83
26722--26782	18	3.4	18	65	24	53	9	36	11	42	1.74
26701--26783	36	2.3	36	91	0	138	8	33	14	43	1.76
37921--37959	19	2	20	85	9	57	35	5	5	53	1.45

Moelleriella zhongdongii

Indices	Period Size	Copy Number	Consensus Size	Percent Matches	Percent Indels	Score	A	C	G	T	Entropy (0-2)
1707--1747	21	1.9	22	80	5	54	56	4	2	36	1.34
5375--5426	6	8.3	6	77	8	58	21	0	5	73	1.04
19608--19651	21	2.1	21	78	0	53	52	9	4	34	1.54
39768--39799	16	1.9	17	93	6	57	46	9	6	37	1.61

Table S8 Ka,Ks,K2P in 15 conserved coding genes and ribiosomal protein of 27 Hypocreales species

atp6			*atp8*			*atp9*			*cob*			*cox1*		
Ka	Ks	K2P	Ka	Ks	K2P	Ka	Ks	K2P	Ka	Ks	K2P	Ka	Ks	K2P
0.0323	0.0591	0.0183	0.0000	0.0619	0.0141	0.0000	0.0178	0.0046	0.0011	0.1481	0.0317	0.0011	0.0361	0.0904
0.0362	0.7277	0.1672	0.0626	0.2635	0.1046	0.0000	0.2246	0.0521	0.0102	0.4677	0.0945	0.0663	0.6844	0.0696
0.0255	0.6732	0.1635	0.0629	0.3022	0.1133	0.0000	0.2246	0.0521	0.0079	0.4016	0.0812	0.0651	0.6665	0.0436
0.0255	0.6722	0.1520	0.0532	0.1412	0.0732	0.0000	0.1779	0.0424	0.0034	0.3029	0.0632	0.0436	0.5364	0.1090
0.0237	0.6059	0.1440	0.0434	0.1428	0.0656	0.0000	0.1779	0.0424	0.0022	0.2972	0.0613	0.0408	0.3651	0.0948
0.0345	0.5942	0.1407	0.0434	0.1428	0.0656	0.0000	0.2005	0.0473	0.0011	0.3087	0.0622	0.0417	0.7419	0.0768
0.0544	0.4773	0.1540	0.0483	0.4231	0.1218	0.0000	0.2480	0.0573	0.0073	0.4435	0.0894	0.0425	0.5815	0.0698
0.1007	0.7428	0.1785	0.0726	0.3528	0.1290	0.0444	0.4444	0.1293	0.0776	0.8645	0.1985	0.0425	0.6049	0.1402
0.0779	0.7766	0.2312	0.0826	0.7186	0.1913	0.0346	0.4980	0.1293	0.0679	0.6078	0.1625	0.0425	0.5815	0.1658
0.0983	0.9265	0.2208	0.1463	0.7807	0.2547	0.0748	0.8641	0.2162	0.0997	0.6124	0.1930	0.0448	0.6759	0.1626
0.1007	0.6899	0.2212	0.0927	0.6439	0.1906	0.0346	0.5333	0.1347	0.0670	0.5626	0.1599	0.1020	0.6080	0.1399
0.0545	0.6998	0.2225	0.0826	0.7186	0.1913	0.0346	0.6939	0.1569	0.0740	0.6078	0.1678	0.0414	0.5564	0.1614
0.0649	0.7716	0.1862	0.0824	0.5173	0.1641	0.0444	0.8601	0.1858	0.0748	0.6817	0.1768	0.0417	0.5573	0.1265
0.0675	1.0324	0.2213	0.0827	0.4529	0.1551	0.0850	1.1813	0.2529	0.1034	0.8867	0.2312	0.0457	0.6125	0.1365
0.0772	0.6557	0.1842	0.0876	0.4843	0.1622	0.0445	0.3253	0.1080	0.0643	0.5979	0.1577	0.0414	0.5648	0.1442
0.0218	0.9169	0.2111	0.1406	0.6694	0.2352	0.0953	0.9374	0.2401	0.0948	0.5717	0.1892	0.0445	0.7772	0.1344
0.0565	0.5051	0.1260	0.0530	0.2204	0.0895	0.0000	0.2488	0.0569	0.0227	0.5281	0.1117	0.0429	0.5643	0.0868
0.0398	0.7451	0.2013	0.0530	0.2620	0.0968	0.0000	0.2738	0.0618	0.0181	0.4529	0.0967	0.0423	0.5978	0.0922
0.0183	0.5149	0.1424	0.0530	0.2204	0.0899	0.0062	0.2506	0.0618	0.0370	0.5701	0.1301	0.0388	0.5824	0.1348
0.0218	0.5142	0.1269	0.0530	0.1810	0.0814	0.0000	0.2719	0.0619	0.0239	0.5170	0.1128	0.0482	0.5519	0.0936
0.0218	0.5051	0.1259	0.0530	0.2204	0.0895	0.0000	0.2246	0.0520	0.0227	0.4744	0.1046	0.0484	0.6353	0.0885
0.1003	0.5051	0.1259	0.0530	0.2204	0.0895	0.0000	0.2246	0.0520	0.0227	0.5203	0.1107	0.0647	0.5427	0.0893
0.0638	0.7044	0.2263	0.0826	0.7186	0.1913	0.0346	0.5703	0.1402	0.0670	0.6045	0.1653	0.0706	0.4986	0.1410
0.0703	0.8725	0.2073	0.0927	0.5136	0.1733	0.0678	1.3308	0.2465	0.1035	0.8404	0.2265	0.0490	0.5249	0.1607
0.0824	0.6616	0.1874	0.0876	0.4843	0.1622	0.0445	0.3253	0.1080	0.0643	0.5991	0.1588	0.0507	0.5222	0.1366
0.0824	1.0086	0.2216	0.1614	0.8713	0.2761	0.0443	0.9286	0.1914	0.0469	0.7355	0.1615	0.0412	0.6416	0.1828
0.0323	0.9868	0.2198	0.1614	0.8713	0.2761	0.0444	0.9178	0.1914	0.0483	0.7448	0.1636	0.0509	0.7762	0.1844
0.0353	0.6560	0.1470	0.0675	0.2854	0.1129	0.0000	0.2488	0.0571	0.0113	0.4051	0.0866	0.0657	0.6794	0.0788
0.0255	0.6526	0.1547	0.0629	0.3483	0.1218	0.0000	0.2488	0.0571	0.0090	0.3500	0.0743	0.0646	0.6874	0.0842
0.0255	0.6584	0.1407	0.0532	0.1780	0.0811	0.0000	0.2005	0.0473	0.0045	0.2852	0.0612	0.0419	0.5181	0.1115
0.0237	0.5810	0.1312	0.0434	0.1800	0.0735	0.0000	0.2005	0.0473	0.0034	0.2741	0.0584	0.0391	0.3395	0.1149
0.0345	0.5942	0.1311	0.0434	0.1800	0.0735	0.0000	0.2239	0.0523	0.0022	0.2796	0.0583	0.0404	0.6877	0.0820
0.0544	0.5100	0.1361	0.0483	0.4775	0.1304	0.0000	0.2729	0.0624	0.0085	0.4021	0.0843	0.0412	0.5304	0.0791
0.1007	0.7428	0.1752	0.0726	0.4026	0.1376	0.0444	0.4770	0.1347	0.0796	0.8675	0.2008	0.0412	0.5522	0.1439
0.0779	0.7766	0.2228	0.0826	0.6439	0.1819	0.0346	0.4980	0.1293	0.0685	0.6017	0.1624	0.0412	0.5304	0.1770
0.0983	0.8697	0.2055	0.1463	0.8699	0.2649	0.0748	0.9213	0.2225	0.0989	0.6517	0.1973	0.0435	0.6263	0.1704
0.1007	0.6899	0.2099	0.0927	0.7186	0.2001	0.0346	0.5333	0.1347	0.0676	0.5733	0.1620	0.1006	0.5546	0.1439
0.0545	0.7145	0.2158	0.0826	0.6439	0.1819	0.0346	0.6939	0.1569	0.0747	0.5848	0.1656	0.0401	0.5572	0.1660
0.0649	0.7878	0.1794	0.0875	0.5482	0.1733	0.0444	0.8601	0.1858	0.0767	0.7037	0.1812	0.0400	0.5757	0.1326
0.0675	0.9883	0.2066	0.0827	0.5099	0.1641	0.0850	1.1012	0.2465	0.1022	0.8355	0.2253	0.0440	0.6322	0.1404
0.0772	0.5902	0.1660	0.0876	0.5441	0.1710	0.0445	0.3529	0.1132	0.0662	0.6445	0.1651	0.0401	0.5656	0.1432
0.0218	0.8977	0.2040	0.1406	0.7461	0.2452	0.0953	0.9374	0.2401	0.0965	0.6178	0.1970	0.0432	0.7780	0.1443

cox2			*cox3*			*nad1*			*nad2*			*nad3*		
Ka	Ks	K2P	Ka	Ks	K2P	Ka	Ks	K2P	Ka	Ks	K2P	Ka	Ks	K2P
0.0334	0.8063	0.1460	0.0539	0.5881	0.1044	0.0411	0.6583	0.1499	0.0690	0.6282	0.1678	0.0277	0.5997	0.1507
0.0041	0.2939	0.0627	0.0858	0.7951	0.0611	0.0246	0.5761	0.1237	0.0387	0.4391	0.1337	0.0038	0.4268	0.2270
0.0000	0.0366	0.0082	0.0876	0.8293	0.0449	0.0060	0.0434	0.0139	0.0114	0.0480	0.0195	0.0000	0.0291	0.1944
0.0313	0.4190	0.1087	0.0912	1.2106	0.1438	0.0446	0.7614	0.1642	0.0799	0.5970	0.1730	0.0530	0.8168	0.1842
0.0041	0.2939	0.0702	0.0992	1.4421	0.1087	0.0390	0.5513	0.1264	0.0335	0.3618	0.1197	0.0312	0.6837	0.2050
0.0041	0.3254	0.0672	0.0781	0.8320	0.0736	0.0321	0.6417	0.1333	0.0306	0.4320	0.1058	0.0278	0.5349	0.1990
0.0061	0.3591	0.0748	0.0740	0.8402	0.0736	0.0293	0.5258	0.1181	0.0288	0.4230	0.1030	0.0038	0.4817	0.2184
0.0999	0.6395	0.1948	0.0854	0.8454	0.1720	0.1081	0.9206	0.2308	0.0938	0.7516	0.2037	0.0540	0.8715	0.2110
0.0621	0.9104	0.1949	0.0873	0.9072	0.2042	0.1142	0.9964	0.2460	0.1147	0.8233	0.2300	0.0634	0.8240	0.2053
0.0647	0.8349	0.1871	0.0390	0.5407	0.2358	0.1110	0.9693	0.2459	0.0993	0.6923	0.1989	0.0982	0.9428	0.2084
0.0969	0.7892	0.2056	0.0419	0.5409	0.1782	0.0980	0.9479	0.2260	0.1158	0.7718	0.2258	0.0635	0.9065	0.0575
0.0414	0.8098	0.1672	0.0373	0.5518	0.2292	0.0786	1.0406	0.2234	0.1138	0.8055	0.2288	0.0593	0.7428	0.1911
0.0134	0.6989	0.1294	0.0389	0.5773	0.1446	0.0595	0.8893	0.1860	0.1033	0.7772	0.2156	0.0498	0.7496	0.1846
0.0155	0.7103	0.1308	0.0389	0.5991	0.1696	0.0603	0.9228	0.1924	0.0954	0.7816	0.2069	0.0876	1.1228	0.1875
0.1039	0.6921	0.2074	0.0441	0.6110	0.1814	0.1018	1.0040	0.2361	0.0712	0.6795	0.1743	0.0763	0.8902	0.1873
0.0342	0.6069	0.1337	0.0445	0.4922	0.1757	0.0320	0.7294	0.1470	0.1060	0.7630	0.2150	0.0896	1.2459	0.2104
0.0041	0.3719	0.0808	0.0496	0.5716	0.1156	0.0368	0.7354	0.1549	0.0340	0.4909	0.1175	0.0115	0.6863	0.1602
0.0061	0.3713	0.0807	0.0419	0.4683	0.1373	0.0724	0.7333	0.1879	0.0361	0.4785	0.1175	0.0115	0.4770	0.1840
0.0061	0.3602	0.0792	0.0376	0.4361	0.1154	0.0410	0.7493	0.1625	0.0670	0.5358	0.1532	0.0292	0.6056	0.1860
0.0041	0.3933	0.0809	0.0419	0.4585	0.1140	0.0320	0.6947	0.1436	0.0357	0.5902	0.1318	0.0154	0.8099	0.1764
0.7864	0.8767	0.6934	0.0288	0.5192	0.1183	0.0320	0.7177	0.1459	0.0349	0.5012	0.1197	0.0115	0.6193	0.2047
0.0041	0.3602	0.0778	0.0484	0.7071	0.1141	0.1035	0.9068	0.2295	0.0349	0.4960	0.1190	0.0115	0.6520	0.2121
0.0976	0.7857	0.2146	0.0518	0.8108	0.1735	0.0749	0.9849	0.2178	0.1183	0.7731	0.2286	0.0635	0.8604	0.1862
0.0339	0.8644	0.1604	0.0898	0.9912	0.2204	0.0792	0.8697	0.2045	0.0966	0.8333	0.2133	0.0896	1.0498	0.1893
0.0342	0.6230	0.1375	0.0916	1.1023	0.1676	0.0885	1.0549	0.2273	0.0712	0.6730	0.1734	0.0763	1.0433	0.1598
0.1329	0.9570	0.2590	0.0831	0.7664	0.2170	0.0886	0.9148	0.2172	0.0834	0.7163	0.1902	0.0889	0.8983	0.2006
0.1268	0.9984	0.2533	0.0826	0.7761	0.2237	0.0330	0.5834	0.1343	0.0804	0.7267	0.1893	0.0931	0.9065	0.1665
0.0294	0.8080	0.1375	0.0765	0.7393	0.1043	0.0378	0.6828	0.1502	0.0676	0.4784	0.1481	0.0318	0.3757	0.1817
0.0333	0.7686	0.1410	0.0782	0.7416	0.1114	0.0351	0.5856	0.1349	0.0595	0.5815	0.1535	0.0277	0.6323	0.1814
0.0542	0.7737	0.1714	0.0869	0.9514	0.1497	0.0351	0.6474	0.1399	0.1081	0.7512	0.2167	0.0510	0.8423	0.1999
0.0333	0.8115	0.1440	0.0946	1.1880	0.1308	0.0282	0.5327	0.1239	0.0716	0.5192	0.1540	0.0292	0.5726	0.1873
0.0295	0.7838	0.1358	0.0772	0.8416	0.0999	0.0337	0.5238	0.1274	0.0585	0.4551	0.1374	0.0000	0.3370	0.2367
0.0316	0.8258	0.1426	0.0776	0.7343	0.1057	0.0878	0.8008	0.2070	0.0621	0.4434	0.1410	0.0317	0.4267	0.2440
0.1210	0.9552	0.2430	0.0758	1.0591	0.1861	0.1071	0.8664	0.2289	0.1024	0.7502	0.2109	0.0817	0.8908	0.2542
0.0848	0.9660	0.2227	0.0810	1.2221	0.2177	0.1070	0.7773	0.2266	0.1414	0.7302	0.2462	0.0815	0.5635	0.2578
0.0897	0.9335	0.2203	0.0271	0.4298	0.2446	0.0821	0.7023	0.1938	0.1088	0.7763	0.2266	0.1202	0.6587	0.2369
0.1171	1.1078	0.2502	0.0365	0.5544	0.1861	0.0768	0.8830	0.2062	0.1405	0.7630	0.2467	0.0816	0.6221	0.2402
0.0691	1.2101	0.2171	0.0347	0.4345	0.2345	0.0535	0.6441	0.1545	0.1380	0.7697	0.2456	0.0773	0.7330	0.2196
0.0476	0.8646	0.1723	0.0271	0.4039	0.1696	0.0533	0.6542	0.1544	0.1126	0.7347	0.2185	0.0774	0.7657	0.0530
0.0456	0.8550	0.1708	0.0271	0.4403	0.1664	0.0814	0.7546	0.1998	0.1184	0.8441	0.2355	0.1145	0.8134	0.2097
0.1260	1.0545	0.2587	0.0313	0.4075	0.1974	0.0247	0.4994	0.1185	0.0937	0.7608	0.2037	0.1027	0.8865	0.1689
0.0706	1.0415	0.2085	0.0442	0.5152	0.1911	0.0293	0.4794	0.1154	0.1123	0.8524	0.2363	0.1142	0.7058	0.1781

nad4			*nad4L*			*nad5*			*nad6*			*rps3*		
Ka	Ks	K2P	Ka	Ks	K2P	Ka	Ks	K2P	Ka	Ks	K2P	Ka	Ks	K2P
0.0336	0.5202	0.1462	0.0000	0.2602	0.0545	0.0192	0.4364	0.1462	0.0110	0.4369	0.1052	0.0069	0.0187	0.1659
0.0143	0.3290	0.1371	0.0000	0.1964	0.0428	0.0049	0.0635	0.1114	0.0240	0.5466	0.1705	0.0811	0.4547	0.1203
0.0009	0.0252	0.2221	0.0000	0.0000	0.0668	0.0440	0.4965	0.1125	0.0073	0.3286	0.1252	0.1083	0.4795	0.1216
0.0330	0.5815	0.1379	0.0049	0.3031	0.0876	0.0440	0.4965	0.1562	0.0546	0.7367	0.1105	0.0775	0.2918	0.1236
0.0365	0.4424	0.1497	0.0149	0.3792	0.0469	0.0446	0.4806	0.1874	0.0184	0.4111	0.1158	0.0734	0.2943	0.1604
0.0157	0.3076	0.1521	0.0000	0.2171	0.0428	0.0195	0.4373	0.1900	0.0073	0.4395	0.2360	0.0775	0.2915	0.1673
0.0157	0.3031	0.1898	0.0000	0.1964	0.0625	0.0195	0.4338	0.1910	0.0147	0.4369	0.2569	0.0916	0.3680	0.3788
0.0517	0.5944	0.1801	0.0124	0.2469	0.1310	0.0527	0.6918	0.1946	0.0903	0.9590	0.3814	0.1160	0.4727	0.3155
0.0557	0.6682	0.2380	0.0224	0.6598	0.1222	0.0743	0.7052	0.1588	0.1609	0.8477	0.2652	0.3137	0.5354	0.3882
0.1152	0.7164	0.0870	0.0249	0.5693	0.1087	0.0838	0.7770	0.2100	0.2826	0.9647	0.2727	0.2535	0.6128	0.3938
0.0577	0.6229	0.0948	0.0299	0.4473	0.1309	0.0808	0.6672	0.1918	0.1661	0.6303	0.2369	0.3224	0.5831	0.2699
0.0568	0.6730	0.1161	0.0224	0.6598	0.0544	0.0745	0.7327	0.1933	0.1685	0.9050	0.3420	0.3100	0.5713	0.2457
0.0495	0.6810	0.1005	0.0124	0.2073	0.1176	0.0610	0.7139	0.1152	0.0949	0.8341	0.2062	0.2183	0.4786	0.1713
0.0714	0.8806	0.0847	0.0509	0.3805	0.0917	0.1019	0.8552	0.1187	0.2254	1.1298	0.4122	0.1651	0.6273	0.3521
0.0828	0.7644	0.0862	0.0174	0.3964	0.1222	0.0878	0.7940	0.1463	0.0826	0.9767	0.1297	0.1223	0.5018	0.0942
0.1315	0.8387	0.1404	0.0249	0.5693	0.0504	0.0898	0.7771	0.1239	0.2785	0.8794	0.1107	0.2923	0.6554	0.1201
0.0249	0.4910	0.1923	0.0049	0.2164	0.0545	0.0196	0.5118	0.1182	0.0314	0.4302	0.1332	0.0680	0.5345	0.1969
0.0268	0.5249	0.1867	0.0049	0.2376	0.1862	0.0242	0.5053	0.1126	0.0333	0.5165	0.1413	0.0748	0.5370	0.1040
0.0542	0.5123	0.1795	0.1520	0.3101	0.0669	0.0542	0.5563	0.1907	0.0314	0.4543	0.1297	0.1611	0.5378	0.0934
0.0235	0.5542	0.1803	0.0049	0.3051	0.0545	0.0253	0.5542	0.2046	0.0352	0.5299	0.1280	0.0807	0.5249	0.0942
0.0240	0.4796	0.1591	0.0049	0.2376	0.0545	0.0196	0.5253	0.1936	0.0314	0.4302	0.2788	0.0688	0.5631	0.3806
0.0249	0.4851	0.2187	0.0049	0.2376	0.1087	0.0196	0.5065	0.1901	0.0314	0.4302	0.3317	0.0680	0.5435	0.2450
0.0582	0.6291	0.1565	0.0299	0.4473	0.0708	0.0767	0.6749	0.1961	0.1706	0.7297	0.2062	0.3145	0.6015	0.1732
0.0623	0.9547	0.1642	0.0275	0.2283	0.0916	0.0943	0.8111	0.1462	0.2199	0.9215	0.3857	0.1621	0.6130	0.3260
0.0829	0.8156	0.0568	0.0174	0.3964	0.1043	0.0886	0.7690	0.1114	0.0785	0.9338	0.3883	0.1210	0.4932	0.3212
0.0848	0.6995	0.1576	0.0352	0.3818	0.1086	0.0896	0.7133	0.1125	0.2780	0.7575	0.1615	0.2346	0.6302	0.1653
0.0853	0.6424	0.1831	0.0352	0.4085	0.0546	0.0905	0.7456	0.1562	0.2784	0.7885	0.0990	0.2363	0.6187	0.1596
0.0307	0.5172	0.2173	0.0000	0.2602	0.0545	0.0199	0.4306	0.1874	0.0110	0.5052	0.0800	0.0777	0.4614	0.1679
0.0336	0.5323	0.1337	0.0000	0.2602	0.0548	0.0329	0.4522	0.1900	0.0036	0.3700	0.0834	0.1047	0.4709	0.2006
0.0438	0.5336	0.1437	0.0049	0.2362	0.0876	0.0329	0.4522	0.1910	0.0430	0.5215	0.2285	0.0740	0.2913	0.1996
0.0474	0.6005	0.1595	0.0149	0.3792	0.0546	0.0398	0.5192	0.1946	0.0128	0.2380	0.2613	0.0699	0.2938	0.4169
0.0297	0.4869	0.1461	0.0000	0.2602	0.0505	0.0021	0.0841	0.1588	0.0036	0.0695	0.3895	0.0740	0.2910	0.3628
0.0297	0.5053	0.1320	0.0000	0.2383	0.0464	0.0021	0.0712	0.2100	0.0110	0.0692	0.2584	0.0845	0.3807	0.4571
0.0635	0.7698	0.1312	0.0124	0.1649	0.1175	0.0451	0.6410	0.1918	0.0782	0.5860	0.2749	0.1144	0.4516	0.4305
0.0666	0.6743	0.1609	0.0224	0.5525	0.1272	0.0680	0.6311	0.1933	0.1551	0.4489	0.2219	0.3099	0.5386	0.2797
0.1291	0.7578	0.1582	0.0274	0.5866	0.1174	0.0784	0.6701	0.1152	0.2671	0.6235	0.3439	0.2481	0.6178	0.2855
0.0666	0.6581	0.1817	0.0299	0.5079	0.1263	0.0764	0.6198	0.1187	0.1609	0.4920	0.1938	0.3169	0.5863	0.2217
0.0677	0.7516	0.1848	0.0224	0.6223	0.0544	0.0695	0.7100	0.1463	0.1655	0.6956	0.3909	0.3064	0.5731	0.3860
0.0612	0.6884	0.1812	0.0124	0.2073	0.1090	0.0561	0.5924	0.1239	0.0820	0.5098	0.1260	0.2137	0.4802	0.1632
0.0766	0.8666	0.2059	0.0509	0.3301	0.0669	0.0916	0.8533	0.1182	0.2151	1.0202	0.1092	0.1665	0.6260	0.1689
0.0914	0.8877	0.0399	0.0174	0.2508	0.1319	0.0799	0.7236	0.1126	0.0725	0.7825	0.1295	0.1192	0.5048	0.2368
0.1422	0.7418	0.0348	0.0274	0.6223	0.0347	0.0830	0.6869	0.1907	0.2683	0.5933	0.1259	0.2846	0.6638	0.1713

0.0578	0.4616	0.1134	0.0530	0.2620	0.0977	0.0000	0.2246	0.0520	0.0216	0.4740	0.1035	0.0412	0.5983	0.0870	0.0326	0.7001	0.1396	0.0390	0.5858	0.1291	0.0647	0.5584	0.1637	0.0619	0.5721	0.1528	0.0398	0.5848	0.2062	0.0322	0.4863	0.1564	0.0049	0.1371	0.0347	0.0150	0.4684	0.2046	0.0277	0.4289	0.1260	0.0646	0.5250	0.1577
0.0398	0.6761	0.1705	0.0530	0.3060	0.1050	0.0000	0.2997	0.0668	0.0170	0.3909	0.0868	0.0434	0.6209	0.0990	0.0348	0.7360	0.1427	0.0416	0.5113	0.1370	0.0318	0.5078	0.1304	0.0583	0.6091	0.1540	0.0398	0.6207	0.1902	0.0341	0.4955	0.1796	0.0049	0.1371	0.1767	0.0173	0.4451	0.1936	0.0315	0.3602	0.1243	0.0713	0.5359	0.1566
0.0183	0.4710	0.1297	0.0530	0.2620	0.0982	0.0062	0.2262	0.0569	0.0359	0.4981	0.1196	0.0400	0.5744	0.1410	0.0347	0.7001	0.1394	0.0458	0.5080	0.1318	0.0247	0.4738	0.1154	0.0911	0.6084	0.1842	0.0581	0.6347	0.2065	0.0570	0.5059	0.1697	0.1520	0.2631	0.0547	0.0506	0.5075	0.1901	0.0277	0.4529	0.2696	0.1547	0.5367	0.4433
0.0218	0.4811	0.1128	0.0530	0.2204	0.0895	0.0000	0.2471	0.0569	0.0228	0.4568	0.1036	0.0465	0.5551	0.0924	0.0291	0.7839	0.1397	0.0416	0.5009	0.1287	0.0247	0.4738	0.1154	0.0635	0.6217	0.1654	0.0438	0.5911	0.2127	0.0322	0.4964	0.2167	0.0049	0.2376	0.0347	0.0163	0.4993	0.1961	0.0315	0.3820	0.3195	0.0796	0.5238	0.2836
0.0218	0.4616	0.1133	0.0530	0.2620	0.0977	0.0000	0.2012	0.0471	0.0216	0.4241	0.0966	0.0467	0.6306	0.0873	0.8265	1.1345	0.7360	0.0424	0.5093	0.1318	0.0885	0.7576	0.2082	0.0627	0.6132	0.1589	0.0398	0.6162	0.2429	0.0312	0.4634	0.1482	0.0049	0.1371	0.0347	0.0150	0.4812	0.1245	0.0277	0.4289	0.1899	0.0646	0.5504	0.2206
0.1003	0.4616	0.1133	0.0530	0.2620	0.0977	0.0000	0.2012	0.0471	0.0216	0.4525	0.1005	0.0635	0.5350	0.0900	0.0326	0.7001	0.1378	0.0585	0.7243	0.1275	0.0729	0.8958	0.2042	0.0627	0.5836	0.1550	0.0398	0.6162	0.1903	0.0322	0.4804	0.1515	0.0049	0.1371	0.1174	0.0150	0.4723	0.1214	0.0277	0.4289	0.3882	0.0646	0.5339	0.3322
0.0638	0.7044	0.2150	0.0826	0.8017	0.2009	0.0346	0.5703	0.1402	0.0676	0.5815	0.1631	0.0694	0.5120	0.1400	0.1174	1.1086	0.2560	0.0602	0.6844	0.1877	0.0701	0.6418	0.1758	0.1431	0.7569	0.2508	0.0816	0.6905	0.1934	0.0666	0.6533	0.1785	0.0299	0.5079	0.0587	0.0742	0.5878	0.1817	0.1654	0.5182	0.3910	0.3098	0.6095	0.3322
0.0703	0.8369	0.1929	0.0927	0.5759	0.1826	0.0678	1.2339	0.2401	0.1023	0.8145	0.2230	0.0472	0.5139	0.1652	0.0614	1.1249	0.2016	0.0882	0.8621	0.2172	0.0858	0.8574	0.2071	0.1176	0.8679	0.2354	0.1165	0.9904	0.2142	0.0699	0.9444	0.1583	0.0275	0.1669	0.0668	0.0842	0.8449	0.1974	0.2105	0.7171	0.0075	0.1635	0.6117	0.0161
0.0824	0.6213	0.1724	0.0876	0.5441	0.1710	0.0445	0.3529	0.1132	0.0662	0.6458	0.1662	0.0489	0.4974	0.1474	0.0706	1.0706	0.2055	0.0895	0.9402	0.1846	0.0859	0.7856	0.1987	0.0937	0.7682	0.2044	0.1027	0.9340	0.2099	0.0915	0.8860	0.1481	0.0174	0.2508	0.1001	0.0808	0.7178	0.2120	0.0685	0.6807	0.1995	0.1180	0.4963	0.0177
0.0824	0.9868	0.2199	0.1558	0.8240	0.2656	0.0443	0.8697	0.1856	0.0470	0.6658	0.1539	0.0395	0.6368	0.1775	0.1521	1.2882	0.2940	0.0771	0.7726	0.2104	0.0214	0.5412	0.1174	0.1117	0.7640	0.2261	0.1180	0.8275	0.0148	0.0977	0.6130	0.1482	0.0352	0.3561	0.1131	0.0892	0.6922	0.2060	0.2626	0.6224	0.2370	0.2349	0.6090	0.1393
0.0377	0.9657	0.2182	0.1558	0.8240	0.2656	0.0444	0.8601	0.1856	0.0484	0.6742	0.1560	0.0492	0.7321	0.1792	0.1458	1.6732	0.3001	0.0769	0.7216	0.2187	0.0386	0.7386	0.1543	0.1113	0.7387	0.2236	0.1223	0.8347	0.1935	0.0977	0.5786	0.0436	0.0352	0.4361	0.0428	0.0875	0.7127	0.2071	0.2630	0.6487	0.3637	0.2367	0.5978	0.1680
0.0225	0.6939	0.1654	0.0560	0.3412	0.1133	0.0000	0.0000	0.0562	0.0119	0.4918	0.0984	0.0000	0.0694	0.0595	0.0041	0.2947	0.0612	0.0016	0.0331	0.0692	0.0276	0.5619	0.1196	0.0349	0.4375	0.1098	0.0038	0.4786	0.2000	0.0134	0.3288	0.1835	0.0000	0.1964	0.0669	0.0437	0.4969	0.1861	0.0165	0.4461	0.2518	0.0820	0.4518	0.3623
0.0225	0.6444	0.1271	0.0275	0.1693	0.0578	0.0000	0.2497	0.0562	0.0113	0.4586	0.0955	0.0633	0.8049	0.1022	0.0312	0.3158	0.0914	0.0922	0.9274	0.1300	0.0126	0.1653	0.0429	0.0805	0.5019	0.1595	0.0573	0.8028	0.2429	0.0335	0.5105	0.1710	0.0049	0.3031	0.0749	0.0437	0.4969	0.2414	0.0392	0.5707	0.2635	0.0651	0.3296	0.3117
0.0207	0.6311	0.1257	0.0182	0.1712	0.0504	0.0000	0.2497	0.0611	0.0102	0.4516	0.0935	0.0570	0.6844	0.0910	0.0041	0.2639	0.0586	0.0980	1.0012	0.0858	0.0046	0.0575	0.0174	0.0306	0.3574	0.0936	0.0353	0.4926	0.2431	0.0283	0.3333	0.1692	0.0149	0.3051	0.0114	0.0461	0.4877	0.2149	0.0241	0.4894	0.2140	0.0617	0.3421	0.3865
0.0423	0.7011	0.1318	0.0182	0.1712	0.0504	0.0000	0.2748	0.0715	0.0090	0.4378	0.0904	0.0592	0.9126	0.0264	0.0000	0.0367	0.0067	0.0138	0.2891	0.0254	0.1001	0.8917	0.2302	0.0197	0.1000	0.0368	0.0318	0.0562	0.2604	0.0028	0.0222	0.1666	0.0000	0.0487	0.0075	0.0203	0.4357	0.2155	0.0147	0.5907	0.3344	0.0651	0.3424	0.3687
0.0525	0.6846	0.1657	0.0369	0.3412	0.0977	0.0000	0.3277	0.1220	0.0130	0.5584	0.1115	0.0598	0.7657	0.0124	0.0020	0.0596	0.0149	0.0130	0.2180	0.0215	0.1061	0.9175	0.2400	0.0215	0.0991	0.0380	0.0077	0.0897	0.2568	0.0028	0.0254	0.2141	0.0000	0.0321	0.0708	0.0203	0.4322	0.1283	0.0222	0.5868	0.1860	0.0849	0.3685	0.2649
0.1002	0.7958	0.1659	0.0656	0.2984	0.1129	0.0465	0.4062	0.1381	0.0784	0.8259	0.1975	0.0598	0.8161	0.1328	0.0998	0.5629	0.1894	0.0711	0.8552	0.1688	0.1033	0.9515	0.2414	0.0937	0.6539	0.1916	0.0582	0.8563	0.2431	0.0502	0.5948	0.2114	0.0124	0.2915	0.1312	0.0531	0.6976	0.1306	0.0824	0.7395	0.3680	0.0957	0.4512	0.2391
0.0804	0.8182	0.2211	0.0804	0.6777	0.1819	0.0345	0.5754	0.2496	0.0735	0.6885	0.1789	0.0598	0.7854	0.1627	0.0591	0.8537	0.1911	0.0784	0.9366	0.1814	0.0951	0.8409	0.2215	0.1219	0.5538	0.2033	0.0615	0.5787	0.2464	0.0510	0.5437	0.2129	0.0224	0.6598	0.1362	0.0739	0.7060	0.1595	0.1591	0.6717	0.1336	0.3054	0.5841	0.1822
0.0982	0.9269	0.2108	0.1327	0.4686	0.1983	0.0747	1.1402	0.1436	0.1044	0.7728	0.2157	0.0657	0.8534	0.1654	0.0608	0.7126	0.1697	0.0687	0.6513	0.2341	0.0746	1.1051	0.2202	0.0960	0.6045	0.1908	0.0941	0.6805	0.1970	0.1194	0.6975	0.2055	0.0274	0.6598	0.1220	0.0791	0.8071	0.1350	0.2711	0.7234	0.1094	0.2444	0.5549	0.3483
0.0996	0.8040	0.2332	0.0854	0.6406	0.1812	0.0345	0.6149	0.1657	0.0720	0.7288	0.1860	0.1195	0.7910	0.1381	0.0968	0.5960	0.1878	0.0777	0.6097	0.1521	0.0578	0.8466	0.1796	0.1227	0.5986	0.2103	0.0616	0.7829	0.2031	0.0550	0.5365	0.2358	0.0299	0.5402	0.1402	0.0779	0.6807	0.1277	0.1650	0.6961	0.1372	0.3198	0.5731	0.1120
0.0525	0.9357	0.2315	0.0804	0.6777	0.1819	0.0345	0.7976	0.1828	0.0791	0.7641	0.1921	0.0619	0.8908	0.1451	0.0392	0.6792	0.1492	0.0767	0.6449	0.2007	0.0578	0.8162	0.1770	0.1192	0.6231	0.2110	0.0574	0.7124	0.2029	0.0520	0.6303	0.1109	0.0224	0.7410	0.0749	0.0727	0.7313	0.1239	0.1697	0.8011	0.1241	0.3112	0.6321	0.1182
0.0611	0.8804	0.1797	0.0558	0.4533	0.1299	0.0465	0.8511	0.2615	0.0747	0.7156	0.1803	0.0589	0.8152	0.1210	0.0175	0.5845	0.1125	0.0687	0.5815	0.1448	0.0921	0.8820	0.2216	0.1044	0.6360	0.1993	0.0541	0.6658	0.1452	0.0501	0.6576	0.2139	0.0124	0.3190	0.1402	0.0584	0.7322	0.1997	0.0883	0.7328	0.1336	0.2097	0.4743	0.2032
0.0584	1.0409	0.2023	0.0755	0.2524	0.1139	0.0848	1.3962	0.1012	0.1059	1.0281	0.2457	0.0601	0.8645	0.1221	0.0155	0.5949	0.1109	0.0687	0.6035	0.1634	0.0288	0.6737	0.1371	0.1048	0.6577	0.2012	0.0900	1.0134	0.1392	0.0658	0.8341	0.2309	0.0509	0.5238	0.1093	0.0985	0.8106	0.2296	0.2204	1.0057	0.1319	0.1540	0.5733	0.1194
0.0800	0.7451	0.1695	0.0854	0.2984	0.1294	0.0465	0.2920	0.2614	0.0667	0.6466	0.1647	0.0596	0.8590	0.1381	0.1038	0.6439	0.2000	0.0722	0.6531	0.1657	0.0309	0.6467	0.1392	0.0773	0.5605	0.1632	0.0786	1.1091	0.0427	0.0774	0.6746	0.2360	0.0174	0.5120	0.1362	0.0872	0.7758	0.2183	0.0767	1.1445	0.2651	0.1056	0.4703	0.1120
0.0260	0.9748	0.2131	0.1271	0.3908	0.1802	0.0951	1.0852	0.0561	0.0988	0.8136	0.2223	0.0637	1.1475	0.1323	0.0399	0.4691	0.1158	0.0733	0.7457	0.1707	0.0691	0.6889	0.1773	0.1007	0.6756	0.2046	0.0855	0.7536	0.0273	0.1383	0.6575	0.2339	0.0274	0.6598	0.0424	0.0859	0.8140	0.2127	0.2820	0.8441	0.3171	0.2848	0.6186	0.1102
0.0503	0.4712	0.1076	0.0274	0.2119	0.0656	0.0000	0.2506	0.0610	0.0216	0.5393	0.1121	0.0578	0.8143	0.0907	0.0041	0.3278	0.0659	0.0697	0.7977	0.0925	0.0405	0.7148	0.1584	0.0430	0.4296	0.1152	0.0155	0.5796	0.0719	0.0214	0.3505	0.2130	0.0049	0.1757	0.0465	0.0199	0.4899	0.2141	0.0296	0.4610	0.1783	0.0438	0.3274	0.3801
0.0431	0.6662	0.1508	0.0274	0.2119	0.0656	0.0000	0.2758	0.0610	0.0193	0.4706	0.1012	0.0618	0.8059	0.0933	0.0061	0.4217	0.0777	0.0706	0.7850	0.1190	0.0288	0.6628	0.1360	0.0432	0.4527	0.1187	0.0155	0.4450	0.0049	0.0238	0.3963	0.2121	0.0049	0.1958	0.1863	0.0243	0.4799	0.0086	0.0353	0.3571	0.3659	0.0515	0.3967	0.2302
0.0224	0.4753	0.1223	0.0274	0.2539	0.0732	0.0062	0.2524	0.0708	0.0359	0.5734	0.1294	0.0620	0.7551	0.1429	0.0061	0.3168	0.0644	0.0750	0.7811	0.0981	0.0288	0.6628	0.1360	0.0746	0.4410	0.1450	0.0333	0.6285	0.0024	0.0498	0.3773	0.2123	0.1520	0.3101	0.0587	0.0545	0.5331	0.1525	0.0296	0.4859	0.3687	0.1365	0.4084	0.1827
0.0260	0.5969	0.1201	0.0274	0.1722	0.0579	0.0000	0.3265	0.0513	0.0228	0.5358	0.1123	0.0674	0.7330	0.0915	0.0041	0.3831	0.0690	0.0706	0.7700	0.1055	0.0986	0.8641	0.2302	0.0431	0.5262	0.1349	0.0194	0.7194	0.2043	0.0168	0.4486	0.2097	0.0049	0.2594	0.0465	0.0263	0.5651	0.1766	0.0334	0.4356	0.2027	0.0534	0.3688	0.3145
0.0260	0.4605	0.1061	0.0274	0.2119	0.0656	0.0000	0.2262	0.0513	0.0216	0.5227	0.1101	0.0674	0.7504	0.0907	0.7990	0.7848	0.6591	0.0750	0.6079	0.0952	0.0706	1.1042	0.2183	0.0438	0.4391	0.1175	0.0155	0.5796	0.2445	0.0205	0.3458	0.2294	0.0049	0.1958	0.0465	0.0199	0.5123	0.1881	0.0296	0.4610	0.2370	0.0436	0.3284	0.3132
0.1002	0.4605	0.1061	0.0274	0.2119	0.0656	0.0000	0.2262	0.1490	0.0216	0.5315	0.1111	0.0807	0.7796	0.0927	0.0041	0.3168	0.0630	0.0569	0.8213	0.0911	0.0701	0.7768	0.1847	0.0438	0.4391	0.1174	0.0155	0.6104	0.1966	0.0214	0.3505	0.1909	0.0049	0.1958	0.1220	0.0199	0.4849	0.1777	0.0296	0.4610	0.3663	0.0438	0.3277	0.0138
0.0590	0.8040	0.2332	0.0754	0.6406	0.1726	0.0345	0.6566	0.2488	0.0720	0.7598	0.1894	0.0849	0.7715	0.1277	0.0975	0.5322	0.1860	0.0621	0.8067	0.1673	0.0900	0.8862	0.2203	0.1237	0.5861	0.2090	0.0616	0.8240	0.2537	0.0550	0.5581	0.1900	0.0299	0.5402	0.0966	0.0756	0.6924	0.1843	0.1696	0.7305	0.2518	0.3116	0.5838	0.1368
0.0637	0.8340	0.1840	0.0854	0.2984	0.1311	0.0676	1.4683	0.1012	0.1048	0.9594	0.2387	0.0657	0.6914	0.1442	0.0317	0.7637	0.1476	0.0835	0.7124	0.1958	0.0901	0.8811	0.2203	0.1055	0.7109	0.2068	0.0920	1.0569	0.2466	0.0597	0.9353	0.0496	0.0275	0.3688	0.1091	0.0925	0.7957	0.1490	0.2157	0.9054	0.2635	0.1558	0.5713	0.1680
0.0870	0.8283	0.1826	0.0854	0.2984	0.1294	0.0465	0.2920	0.2061	0.0666	0.6479	0.1659	0.0692	0.7017	0.1305	0.0399	0.4825	0.1175	0.0853	0.7574	0.1690	0.0442	0.7524	0.1655	0.0773	0.5775	0.1654	0.0786	1.1730	0.0610	0.0774	0.7201	0.1547	0.0174	0.5120	0.1175	0.0880	0.7643	0.2168	0.0726	1.2136	0.2181	0.1069	0.4777	0.3601
0.0870	0.8312	0.2111	0.1314	1.2555	0.2761	0.0442	1.1553	0.2063	0.0516	0.8489	0.1767	0.0596	0.8007	0.1745	0.1373	0.9944	0.2603	0.0701	0.6806	0.2005	0.0358	0.5089	0.1190	0.0907	0.6210	0.1892	0.0935	0.8381	0.0665	0.0851	0.6260	0.1744	0.0352	0.4648	0.1130	0.0877	0.7317	0.1920	0.2762	0.6622	0.3392	0.2340	0.7294	0.3158
0.0246	0.7971	0.2075	0.1314	1.2555	0.2761	0.0443	1.1392	0.0562	0.0506	0.8363	0.1744	0.0707	0.9093	0.1727	0.1311	0.9836	0.2546	0.0708	0.6933	0.2070	0.0289	0.6036	0.1269	0.0876	0.6302	0.1884	0.0977	0.8454	0.0376	0.0851	0.6405	0.1766	0.0352	0.4361	0.0668	0.0885	0.7725	0.1955	0.2766	0.7232	0.1918	0.2382	0.6585	0.3829
0.0246	0.6750	0.1252	0.0467	0.1301	0.0656	0.0000	0.2497	0.0562	0.0045	0.3999	0.0803	0.0622	0.8355	0.1012	0.0312	0.3837	0.1023	0.0921	0.8267	0.1301	0.0262	0.4932	0.1119	0.0766	0.5839	0.1687	0.0530	0.8168	0.0401	0.0340	0.5545	0.2205	0.0049	0.3031	0.0876	0.0000	0.0000	0.1035	0.0449	0.5116	0.3680	0.0856	0.4073	0.3677
0.0264	0.7326	0.1637	0.0370	0.1315	0.0582	0.0000	0.2497	0.0611	0.0056	0.3999	0.0813	0.0558	0.7014	0.0818	0.0041	0.2738	0.0657	0.0980	0.9654	0.1042	0.1046	0.8797	0.2303	0.0270	0.3575	0.0916	0.0312	0.7570	0.1921	0.0375	0.4533	0.1472	0.0149	0.3792	0.0469	0.0589	0.5490	0.1028	0.0165	0.3158	0.1410	0.0857	0.3996	0.2623
0.0538	0.7043	0.2432	0.0370	0.1315	0.0582	0.0000	0.2748	0.0715	0.0068	0.4130	0.0841	0.0592	1.0129	0.0643	0.0041	0.3263	0.0658	0.0154	0.2740	0.0761	0.1137	0.9673	0.2495	0.0237	0.4065	0.0961	0.0278	0.5954	0.2520	0.0148	0.3074	0.1548	0.0000	0.2171	0.0428	0.0341	0.4531	0.1390	0.0073	0.3295	0.1165	0.0868	0.4357	0.2367
0.0657	0.6468	0.2207	0.0370	0.3831	0.1055	0.0000	0.3277	0.1220	0.0107	0.4776	0.0979	0.0587	0.8471	0.0567	0.0061	0.3602	0.0733	0.0146	0.1900	0.0775	0.1105	0.9732	0.2507	0.0219	0.3978	0.0933	0.0038	0.5093	0.2132	0.0148	0.3029	0.1775	0.0000	0.1964	0.0625	0.0341	0.4624	0.1141	0.0147	0.3277	0.1446	0.1086	0.4193	0.1778
0.1130	0.8015	0.2405	0.0465	0.2493	0.0884	0.0465	0.4062	0.1381	0.0734	0.9313	0.2019	0.0587	0.8805	0.1333	0.0998	0.6252	0.1913	0.0693	0.8095	0.1751	0.0943	0.9080	0.2255	0.0854	0.7130	0.1919	0.0540	0.8715	0.2402	0.0527	0.5738	0.1514	0.0124	0.2469	0.1310	0.0551	0.6431	0.1053	0.0842	0.6674	0.1313	0.1123	0.5399	0.3511
0.0983	0.8183	0.2469	0.0561	0.4975	0.1376	0.0345	0.5754	0.2496	0.0640	0.5654	0.1540	0.0587	0.8471	0.1679	0.0621	0.9147	0.1930	0.0765	0.9399	0.2142	0.0815	1.0390	0.2295	0.1088	0.7655	0.2186	0.0634	0.8240	0.2332	0.0557	0.6452	0.1463	0.0224	0.6598	0.1222	0.0788	0.6805	0.1009	0.1593	0.6783	0.1410	0.3140	0.6888	0.1142
0.1116	0.8369	0.1693	0.1226	0.7196	0.2251	0.0747	1.1402	0.1436	0.0948	0.6006	0.1861	0.0657	0.9330	0.1640	0.0647	0.8612	0.1870	0.0708	0.6258	0.2393	0.0574	0.8386	0.1796	0.0930	0.6790	0.1918	0.0982	0.9428	0.0965	0.1163	0.7158	0.1472	0.0249	0.5693	0.1087	0.0795	0.7451	0.1734	0.2745	0.8174	0.1393	0.2582	0.7257	0.1186
0.1137	0.7348	0.1947	0.0610	0.3632	0.1204	0.0345	0.6149	0.1657	0.0625	0.5423	0.1548	0.1179	0.8687	0.1368	0.0968	0.7722	0.2020	0.0795	0.6114	0.1673	0.0567	0.8699	0.1864	0.1081	0.7390	0.2153	0.0635	0.9557	0.0273	0.0577	0.6224	0.0105	0.0299	0.4473	0.1309	0.0882	0.6860	0.2074	0.1652	0.5489	0.2651	0.3326	0.7574	0.2008
0.0657	0.9217	0.1596	0.0561	0.3882	0.1206	0.0345	0.7976	0.1828	0.0696	0.5295	0.1539	0.0608	0.9014	0.1540	0.0435	0.8133	0.1689	0.0785	0.6726	0.2274	0.0981	0.9610	0.2356	0.1079	0.7491	0.2175	0.0593	0.7428	0.0247	0.0568	0.6499	0.1791	0.0224	0.6598	0.0544	0.0820	0.7519	0.1925	0.1698	0.7722	0.3218	0.3138	0.7631	0.1216
0.0837	0.7988	0.2267	0.0560	0.2946	0.1044	0.0465	0.8511	0.2615	0.0716	0.7561	0.1822	0.0578	0.8572	0.1219	0.0134	0.6487	0.1228	0.0707	0.5811	0.1569	0.0316	0.7091	0.1449	0.0928	0.7527	0.2036	0.0498	0.7496	0.2176	0.0505	0.6577	0.1806	0.0124	0.2073	0.1176	0.0632	0.6540	0.1903	0.0880	0.7470	0.1841	0.2067	0.5758	0.1142

0.0733	0.8665	0.1148
0.0973	0.9679	0.1641
0.0317	0.7650	0.1297
0.0659	0.6556	0.1149
0.0499	0.7715	0.1162
0.0290	0.6812	0.1162
0.0317	0.7615	0.2441
0.0317	0.6556	0.1850
0.1136	0.6556	0.1612
0.0768	0.7821	0.2237
0.0774	0.7370	0.2220
0.0992	0.9423	0.2004
0.0992	0.7836	0.2599
0.0017	0.7675	0.2455
0.0309	0.0896	0.2534
0.0573	0.1630	0.2636
0.1094	0.5760	0.1991
0.0915	0.8254	0.2421
0.1070	0.9844	0.2031
0.1097	0.9576	0.2456
0.0573	0.8914	0.1363
0.0666	1.0244	0.1972
0.0527	0.8397	0.1501
0.0905	1.0483	0.1347
0.0175	0.7218	0.1362
0.0437	1.0600	0.1362
0.0308	0.5773	0.2569
0.0139	0.7703	0.2198
0.0175	0.6068	0.2079
0.0175	0.6933	0.2380
0.1090	0.5773	0.2381
0.0636	0.5773	0.1869
0.0579	0.9300	0.1790
0.0885	0.8499	0.1996
0.0885	0.7231	0.1936
0.0017	0.9150	0.0359
0.0309	0.8959	0.1557
0.0573	0.1423	0.1281
0.1084	0.5405	0.1821
0.0928	0.8254	0.1108
0.1060	0.9330	0.1765
0.1087	0.9644	0.1258
0.0573	0.8283	0.1510
0.0678	0.9703	0.1093
0.0536	0.8397	0.1093
0.0918	0.9892	0.1996

0.0961	0.4378	0.1635
0.0659	0.6254	0.1624
0.1171	0.6157	0.2066
0.0275	0.2082	0.0659
0.0275	0.2493	0.0732
0.0275	0.2493	0.0735
0.0275	0.1693	0.0582
0.0275	0.2082	0.0659
0.0275	0.2082	0.0659
0.0561	0.3882	0.1206
0.0858	0.3882	0.1458
0.0659	0.6254	0.1624
0.1537	0.7577	0.2547
0.1537	0.7577	0.2547
0.0091	0.0000	0.0070
0.0091	0.0000	0.0070
0.0277	0.2047	0.0656
0.0562	0.1683	0.0806
0.0660	0.4378	0.1380
0.1282	0.7532	0.2349
0.0759	0.3856	0.1376
0.0660	0.4378	0.1380
0.0659	0.2928	0.1129
0.0661	0.2464	0.1042
0.0759	0.4378	0.1453
0.1226	0.6455	0.2160
0.0183	0.0629	0.0284
0.0183	0.0964	0.0356
0.0183	0.0964	0.0356
0.0183	0.0308	0.0212
0.0183	0.0629	0.0284
0.0183	0.0629	0.0284
0.0660	0.4378	0.1380
0.0759	0.2910	0.1206
0.0759	0.4378	0.1453
0.1216	1.0552	0.2553
0.1216	1.0552	0.2553
0.0000	0.0000	0.0582
0.0183	0.2070	0.0730
0.0464	0.1702	0.1299
0.0560	0.4439	0.2255
0.1171	0.7662	0.1294
0.0658	0.3908	0.1299
0.0560	0.4439	0.1050
0.0559	0.2965	0.0963
0.0561	0.2493	0.1369

0.0848	1.3962	0.1012
0.0465	0.2920	0.2614
0.0951	1.0852	0.0561
0.0000	0.2506	0.0610
0.0000	0.2758	0.0610
0.0062	0.2524	0.0708
0.0000	0.3265	0.0513
0.0000	0.2262	0.0513
0.0000	0.2262	0.1490
0.0345	0.6566	0.2488
0.0676	1.4683	0.1012
0.0465	0.2920	0.2061
0.0442	1.1553	0.2063
0.0443	1.1392	0.0045
0.0000	0.0000	0.0515
0.0000	0.0179	0.1063
0.0000	0.2246	0.1221
0.0379	0.3556	0.2123
0.0282	0.5001	0.1276
0.0747	0.7653	0.1436
0.0282	0.5356	0.1547
0.0282	0.6535	0.2489
0.0379	0.6686	0.1115
0.0780	1.2808	0.2364
0.0444	0.3543	0.0659
0.0882	0.8834	0.0513
0.0000	0.3008	0.0610
0.0000	0.2254	0.0759
0.0062	0.2515	0.0610
0.0000	0.3529	0.0610
0.0000	0.2748	0.1329
0.0000	0.2748	0.2426
0.0282	0.5729	0.1115
0.0610	1.4550	0.1943
0.0444	0.3543	0.1946
0.0378	1.0692	0.0045
0.0379	1.0554	0.0515
0.0000	0.0179	0.1063
0.0000	0.2246	0.1221
0.0379	0.3556	0.2123
0.0282	0.5001	0.1276
0.0747	0.7653	0.1436
0.0282	0.5356	0.1547
0.0282	0.6535	0.2489
0.0379	0.6686	0.1115
0.0780	1.2808	0.2364

0.0983	0.8126	0.2196
0.0644	0.6675	0.1649
0.0906	0.6236	0.1914
0.0216	0.4887	0.1047
0.0176	0.4140	0.0899
0.0359	0.5286	0.1229
0.0228	0.4359	0.0998
0.0216	0.4517	0.0997
0.0216	0.4813	0.1038
0.0625	0.5344	0.1537
0.0978	0.7238	0.2093
0.0643	0.6504	0.1639
0.0470	0.7232	0.1604
0.0460	0.7323	0.1603
0.0011	0.0154	0.0043
0.0022	0.0473	0.0121
0.0062	0.4768	0.0912
0.0728	0.7268	0.1796
0.0659	0.6281	0.1637
0.0989	0.6780	0.2012
0.0650	0.6608	0.1710
0.0720	0.6930	0.1769
0.0710	0.6990	0.1748
0.0983	0.9325	0.2300
0.0632	0.5700	0.1551
0.0947	0.6658	0.2040
0.0216	0.4040	0.0936
0.0170	0.3710	0.0858
0.0359	0.4538	0.1136
0.0228	0.3822	0.0927
0.0216	0.3774	0.0897
0.0216	0.3974	0.0927
0.0650	0.6796	0.1733
0.0985	0.8592	0.2230
0.0631	0.5711	0.1551
0.0470	0.7216	0.1592
0.0484	0.7826	0.1668
0.0011	0.0473	0.0112
0.0051	0.4555	0.0873
0.0740	0.7068	0.1785
0.0671	0.6371	0.1659
0.1002	0.6780	0.2023
0.0662	0.6608	0.1721
0.0733	0.6930	0.1779
0.0722	0.6990	0.1759
0.0971	0.9458	0.2300

0.0602	0.9212	0.1333
0.0584	0.9152	0.1396
0.0626	1.2310	0.1336
0.0578	0.8674	0.0749
0.0618	0.8257	0.0810
0.0620	0.7639	0.1277
0.0662	0.7323	0.0844
0.0663	0.7592	0.0767
0.0795	0.8092	0.0778
0.0849	0.8007	0.1341
0.0657	0.6907	0.1605
0.0680	0.7283	0.1351
0.0596	0.7999	0.1798
0.0707	1.0224	0.1799
0.0086	0.4073	0.1217
0.0206	0.6197	0.1076
0.0223	0.5382	0.1040
0.0223	0.5310	0.1507
0.0223	0.5310	0.1809
0.0218	0.5537	0.1833
0.0802	0.5781	0.1484
0.0279	0.6279	0.1697
0.0273	0.5576	0.1227
0.0307	0.6582	0.1403
0.0279	0.5970	0.1478
0.0290	0.7862	0.1550
0.0284	0.6031	0.0869
0.0415	0.8213	0.0901
0.0445	0.7451	0.1417
0.0312	0.5913	0.0957
0.0312	0.6552	0.0886
0.0802	0.6038	0.0890
0.0820	0.6379	0.1461
0.0318	0.5274	0.1720
0.0334	0.5538	0.1598
0.0257	0.6934	0.2046
0.0251	0.7195	0.2013
0.0234	0.4887	0.0956
0.0201	0.3986	0.0921
0.0201	0.4046	0.1614
0.0201	0.3986	0.1892
0.0246	0.5559	0.1924
0.0784	0.4399	0.1588
0.0296	0.6141	0.1725
0.0290	0.5826	0.1473
0.0324	0.6606	0.1521

0.0155	0.7318	0.1308
0.1038	0.6770	0.2038
0.0363	0.5777	0.1319
0.0041	0.3273	0.0731
0.0061	0.3493	0.0762
0.0061	0.3163	0.0716
0.0041	0.3945	0.0794
0.7912	0.8350	0.6866
0.0041	0.3163	0.0701
0.0975	0.7688	0.2110
0.0360	0.8683	0.1605
0.0363	0.5932	0.1342
0.1328	1.0145	0.2629
0.1267	1.0309	0.2552
0.0270	0.4337	0.1052
0.0313	0.3713	0.0991
0.0334	0.4073	0.1052
0.1139	0.6929	0.2106
0.0769	0.7646	0.1980
0.0747	0.7051	0.1833
0.1109	0.7509	0.2123
0.0572	0.7458	0.1741
0.0355	0.6561	0.1333
0.0334	0.7209	0.1365
0.1193	0.7209	0.2159
0.0507	0.5974	0.1467
0.0312	0.3964	0.0993
0.0291	0.4858	0.1068
0.0334	0.3964	0.0993
0.0292	0.5545	0.1168
0.8074	0.7971	0.6684
0.0312	0.3964	0.0978
0.1116	0.7284	0.2157
0.0523	0.8778	0.1725
0.0507	0.6133	0.1481
0.1598	1.0735	0.2942
0.1534	1.0330	0.2822
0.0041	0.3050	0.0645
0.0061	0.3380	0.0734
0.1021	0.6602	0.2071
0.0576	0.8925	0.1964
0.0602	0.8631	0.1834
0.0991	0.7942	0.2105
0.0435	0.7156	0.1610
0.0134	0.6331	0.1159
0.0113	0.7145	0.1205

0.0707	0.6032	0.1871
0.0740	0.6550	0.1861
0.0751	0.6922	0.1802
0.0678	0.8002	0.1111
0.0724	0.7288	0.1464
0.0767	0.7250	0.1168
0.0724	0.7148	0.1113
0.0767	0.6095	0.1082
0.0586	0.7474	0.1096
0.0638	0.7341	0.1798
0.0853	0.7007	0.2272
0.0870	0.7451	0.1784
0.0718	0.6693	0.2304
0.0726	0.6555	0.2389
0.0373	0.6044	0.1334
0.0998	0.8067	0.1345
0.0997	0.7070	0.1375
0.0835	1.2582	0.2006
0.0899	1.1076	0.2419
0.0752	0.7057	0.2637
0.0786	0.9401	0.1942
0.0842	0.9454	0.2160
0.0751	0.7672	0.1586
0.0751	0.7502	0.1730
0.0823	0.8595	0.1975
0.0870	0.8226	0.1962
0.0761	1.0124	0.1231
0.0843	0.8496	0.1445
0.0887	0.8456	0.1288
0.0843	0.8333	0.1186
0.0806	0.9840	0.1144
0.0654	0.8680	0.1216
0.0642	0.8809	0.2055
0.0887	0.7675	0.2073
0.0914	0.8401	0.1962
0.0770	0.8567	0.2239
0.0768	0.7548	0.2342
0.0974	1.0305	0.0872
0.0961	0.8778	0.0885
0.0850	1.0203	0.1720
0.0961	1.1689	0.2094
0.0863	1.1553	0.2374
0.0956	1.0742	0.1720
0.0954	1.1076	0.2155
0.0862	1.0710	0.1523
0.0862	1.0669	0.1633

0.0336	0.6920	0.1492
0.0719	0.7131	0.1842
0.0377	0.6937	0.1557
0.0316	0.6753	0.1415
0.0316	0.6976	0.1438
0.1001	0.8956	0.2315
0.0778	0.9671	0.2226
0.0741	0.8340	0.1971
0.0878	0.9915	0.2246
0.0879	0.8910	0.2171
0.0331	0.7367	0.1518
0.0317	0.6936	0.1438
0.0338	0.7105	0.1475
0.0952	0.9213	0.2328
0.1102	0.9494	0.2439
0.1082	0.9125	0.2428
0.0930	0.7775	0.2142
0.0803	0.8997	0.2094
0.0593	0.7164	0.1682
0.0600	0.6746	0.1612
0.0900	0.7641	0.2155
0.0261	0.5407	0.1199
0.0302	0.5050	0.1134
0.0650	0.5814	0.1613
0.0336	0.6142	0.1371
0.0261	0.4963	0.1146
0.0261	0.5316	0.1188
0.0967	0.8500	0.2290
0.0809	0.8979	0.2132
0.0792	0.7346	0.1945
0.0909	0.7793	0.2121
0.0909	0.7147	0.2036
0.0228	0.5960	0.1160
0.0255	0.5395	0.1120
0.1008	0.8547	0.2217
0.1189	0.9250	0.2483
0.1142	0.9002	0.2483
0.0970	0.7827	0.2155
0.0820	1.0280	0.2255
0.0564	0.7538	0.1705
0.0564	0.7155	0.1644
0.0933	0.9126	0.2243
0.0364	0.6042	0.1340
0.0413	0.6720	0.1471
0.0764	0.6763	0.1810
0.0412	0.6959	0.1528

0.0879	0.7631	0.1983
0.0637	0.6453	0.1637
0.1013	0.7290	0.2072
0.0235	0.4608	0.1042
0.0255	0.4537	0.1050
0.0560	0.4984	0.1386
0.0267	0.5506	0.1190
0.0243	0.4707	0.1064
0.0243	0.4657	0.1057
0.1106	0.7403	0.2181
0.0890	0.8056	0.2038
0.0637	0.6390	0.1629
0.0786	0.6993	0.1841
0.0756	0.7094	0.1832
0.0773	0.5678	0.1671
0.0550	0.5160	0.1596
0.0741	0.5059	0.1551
0.1314	0.6664	0.2274
0.1585	0.7195	0.2566
0.1372	0.7365	0.2390
0.1566	0.7670	0.2615
0.1597	0.7378	0.2621
0.1397	0.6748	0.2364
0.1338	0.7798	0.2437
0.1164	0.6781	0.2146
0.1409	0.7704	0.2485
0.0751	0.4367	0.1446
0.0674	0.4912	0.1476
0.1059	0.4704	0.1762
0.0735	0.4822	0.1512
0.0743	0.4560	0.1469
0.0743	0.4367	0.1439
0.1574	0.7458	0.2601
0.1367	0.7783	0.2445
0.1164	0.6980	0.2170
0.1262	0.6978	0.2258
0.1290	0.7251	0.2308
0.0383	0.3845	0.1048
0.0364	0.3675	0.1006
0.0978	0.7263	0.2038
0.1226	0.7353	0.2275
0.1051	0.6688	0.2011
0.1216	0.7122	0.2241
0.1226	0.7552	0.2296
0.1061	0.7181	0.2105
0.1038	0.7281	0.2085

0.0876	1.1228	0.2587
0.0763	0.8902	0.2227
0.0896	1.2459	0.2747
0.0115	0.6520	0.2674
0.0115	0.5323	0.0748
0.0292	0.5746	0.0747
0.0154	0.8983	0.2210
0.0115	0.5878	0.2478
0.0115	0.6193	0.2535
0.0635	0.9065	0.2566
0.0896	1.0498	0.2461
0.0763	1.0433	0.0024
0.0889	0.8983	0.2045
0.0931	0.9065	0.2484
0.0472	1.1140	0.1967
0.0510	0.8348	0.2539
0.0572	0.8532	0.2468
0.0893	0.7394	0.2076
0.0927	0.8840	0.2481
0.1274	1.0922	0.1999
0.0929	1.0290	0.2573
0.0885	0.7166	0.2501
0.0850	0.9154	0.2130
0.1179	1.2166	0.1975
0.0974	0.9065	0.1842
0.1222	1.5049	0.1904
0.0574	1.0309	0.2065
0.0573	0.7994	0.1599
0.0761	1.1263	0.1628
0.0532	1.1722	0.1903
0.0574	1.0892	0.1967
0.0574	1.0892	0.0216
0.0929	1.2216	0.1698
0.1177	0.8874	0.1707
0.0974	0.9557	0.2167
0.1170	1.1069	0.1919
0.1213	1.1191	0.1767
0.0292	0.4578	0.1635
0.0373	0.4824	0.2617
0.0922	1.1555	0.2330
0.0849	0.7194	0.2268
0.1285	0.9737	0.1110
0.0850	0.8745	0.1082
0.0806	0.8381	0.1370
0.0879	0.7637	0.1287
0.1078	1.1689	0.1112

0.0714	0.8699	0.1753
0.0839	0.7387	0.1744
0.1326	0.8379	0.1607
0.0259	0.4673	0.1725
0.0278	0.4999	0.1846
0.0552	0.4880	0.2232
0.0226	0.5410	0.1549
0.0249	0.4561	0.1601
0.0259	0.4615	0.1848
0.0582	0.6143	0.1613
0.0633	0.9537	0.1541
0.0839	0.7881	0.1541
0.0859	0.6911	0.0503
0.0864	0.6346	0.1746
0.0458	0.5904	0.1868
0.0345	0.5167	0.1718
0.0344	0.5109	0.1709
0.0551	0.6830	0.1680
0.0641	0.7175	0.1922
0.1190	0.7847	0.2144
0.0616	0.6727	0.1497
0.0627	0.7603	0.1513
0.0521	0.6972	0.1756
0.0669	0.7885	0.1556
0.0858	0.9026	0.1480
0.1357	0.8186	0.1472
0.0335	0.4424	0.1590
0.0354	0.4919	0.1714
0.0602	0.4565	0.1880
0.0331	0.4325	0.1715
0.0335	0.4371	0.1706
0.0335	0.4368	0.2020
0.0616	0.6834	0.2151
0.0592	0.9427	0.1813
0.0858	0.9428	0.1927
0.0873	0.7858	0.2043
0.0868	0.7467	0.1863
0.0312	0.3285	0.1813
0.0312	0.3239	0.1786
0.0691	0.6971	0.1744
0.0733	0.7390	0.0506
0.1285	0.8301	0.2044
0.0774	0.7213	0.1844
0.0723	0.8325	0.1835
0.0705	0.7827	0.2427
0.0798	0.8340	0.1903

0.0509	0.3805	0.0917
0.0174	0.3964	0.1222
0.0249	0.5693	0.0504
0.0049	0.2164	0.0545
0.0049	0.2376	0.1862
0.1520	0.3101	0.0669
0.0049	0.3051	0.0545
0.0049	0.2376	0.0545
0.0049	0.2376	0.1087
0.0299	0.4473	0.0708
0.0275	0.2283	0.0916
0.0174	0.3964	0.1043
0.0352	0.3818	0.1086
0.0352	0.4085	0.0666
0.0200	0.4565	0.1358
0.0049	0.3031	0.1409
0.0049	0.2801	0.1265
0.0175	0.3119	0.1449
0.0275	0.7742	0.0708
0.0300	0.7968	0.1356
0.0377	0.7814	0.1049
0.0275	0.9210	0.1409
0.0174	0.3401	0.0385
0.0563	0.5179	0.0424
0.0225	0.4761	0.1766
0.0300	0.8440	0.0546
0.0099	0.2145	0.0424
0.0099	0.2145	0.0424
0.1565	0.3397	0.1265
0.0099	0.3258	0.0922
0.0099	0.2145	0.1047
0.0099	0.2145	0.1220
0.0377	0.7814	0.1174
0.0327	0.3159	0.1133
0.0225	0.4761	0.1091
0.0405	0.5518	0.1132
0.0405	0.5518	0.1224
0.0149	0.3537	0.0268
0.0149	0.3537	0.1138
0.0276	0.4710	0.0796
0.0377	0.7814	0.1136
0.0377	0.7814	0.0345
0.0350	0.6435	0.0385
0.0377	0.7814	0.1767
0.0275	0.4207	0.0669
0.0457	0.5539	0.0385

0.0987	0.8094	0.1923
0.0889	0.7372	0.0950
0.0851	0.7295	0.1102
0.0382	0.4501	0.1102
0.0395	0.4679	0.1232
0.0718	0.4665	0.0189
0.0410	0.4891	0.0163
0.0382	0.4713	0.1465
0.0382	0.4539	0.1721
0.0871	0.5588	0.1817
0.0896	0.8364	0.1738
0.0899	0.7439	0.1785
0.0905	0.7228	0.1452
0.0908	0.7735	0.2087
0.0589	0.5490	0.1833
0.0341	0.4531	0.1876
0.0341	0.4624	0.0971
0.0551	0.6431	0.0959
0.0788	0.6805	0.1336
0.0795	0.7451	0.1046
0.0882	0.6860	0.0989
0.0820	0.7519	0.0946
0.0632	0.6540	0.1684
0.0987	0.8094	0.2001
0.0889	0.7372	0.1838
0.0851	0.7295	0.1864
0.0382	0.4501	0.1877
0.0395	0.4679	0.1266
0.0718	0.4665	0.1266
0.0410	0.4891	0.1244
0.0382	0.4713	0.0962
0.0382	0.4539	0.0956
0.0871	0.6888	0.1595
0.0896	0.8364	0.1849
0.0899	0.7439	0.1955
0.0905	0.7228	0.1809
0.0908	0.7735	0.1836
0.0398	0.5271	0.1632
0.0391	0.5093	0.2108
0.0740	0.7282	0.1950
0.0900	0.6744	0.2027
0.1014	0.7681	0.1036
0.1009	0.7242	0.1059
0.0930	0.7736	0.1391
0.0849	0.7324	0.1197
0.1187	0.9505	0.1065

0.2220	1.2467	0.3659
0.0765	0.7885	0.3687
0.2757	0.8206	0.0991
0.0333	0.4435	0.1305
0.0352	0.3842	0.0574
0.0333	0.4681	0.1897
0.0371	0.3732	0.1117
0.0333	0.4435	0.0973
0.0333	0.4435	0.0973
0.1697	0.5774	0.2597
0.2174	0.8172	0.2867
0.0724	0.7546	0.4224
0.2700	0.7074	0.2787
0.2704	0.7367	0.2959
0.0567	0.5314	0.2470
0.0468	0.5248	0.3609
0.0469	0.5215	0.2215
0.1154	0.7819	0.4145
0.1597	0.8140	0.1333
0.2804	0.9331	0.1257
0.1566	0.7662	0.1369
0.1702	1.0790	0.1424
0.1067	0.9205	0.1333
0.2274	1.2253	0.1316
0.1096	1.0976	0.2950
0.3075	1.1629	0.3460
0.0526	0.5697	0.2175
0.0487	0.4874	0.3996
0.0526	0.5418	0.4026
0.0545	0.4748	0.1071
0.0526	0.5697	0.0818
0.0526	0.5697	0.1573
0.1610	0.7301	0.0787
0.1200	1.0643	0.0150
0.1095	0.9487	0.0227
0.2864	0.8077	0.2090
0.2868	0.8819	0.2326
0.0110	0.2484	0.3610
0.0184	0.2471	0.2495
0.0885	0.6579	0.2658
0.1531	0.7543	0.2100
0.2859	0.7318	0.3391
0.1589	0.6765	0.1859
0.1620	0.9156	0.3629
0.0923	0.5754	0.1264
0.2275	0.8909	0.1094

0.1655	0.6755	0.1124
0.1203	0.6232	0.3766
0.2995	0.7161	0.2279
0.0647	0.5271	0.1768
0.0733	0.5165	0.3150
0.1548	0.4991	0.3138
0.0791	0.5559	0.1416
0.0657	0.5202	0.1722
0.0647	0.5193	0.3644
0.3210	0.7378	0.3117
0.1658	0.6851	0.3886
0.1203	0.6132	0.3708
0.2333	0.7222	0.2700
0.2326	0.7372	0.2450
0.0053	0.0385	0.1788
0.0085	0.0429	0.3467
0.0758	0.2103	0.1163
0.1011	0.4173	0.1189
0.2921	0.5345	0.1997
0.2428	0.4887	0.1255
0.3109	0.5342	0.1163
0.2958	0.5472	0.1145
0.2141	0.4130	0.3823
0.1486	0.4760	0.2361
0.1099	0.4350	0.1778
0.2774	0.5362	0.3191
0.0524	0.3354	0.3179
0.0570	0.3492	0.2154
0.1452	0.3458	0.3942
0.0582	0.3603	0.3581
0.0534	0.3365	0.4134
0.0524	0.3228	0.3967
0.3023	0.5558	0.2935
0.1468	0.4727	0.2800
0.1087	0.4346	0.2188
0.2220	0.6128	0.3939
0.2256	0.5807	0.1583
0.0053	0.0384	0.1649
0.0735	0.2047	0.2454
0.0994	0.4201	0.1673
0.2915	0.5462	0.1565
0.2421	0.4998	0.1565
0.3134	0.5206	0.4041
0.2951	0.5591	0.2816
0.2142	0.4195	0.2167
0.1444	0.5088	0.3592

0.0175	0.6714	0.1639	0.0658	0.4439	0.2069	0.0444	0.3543	0.0659	0.0619	0.5618	0.1529	0.0290	0.5876	0.1680	0.1061	0.7145	0.2105	0.0935	1.0891	0.1846	0.0364	0.5942	0.1330	0.0800	0.6522	0.1770	0.1048	1.1473	0.1139	0.0924	0.8007	0.1975	0.0174	0.4796	0.0385	0.1062	0.8003	0.1004	0.0787	0.9178	0.1299	0.1075	0.4569	0.3529
0.0437	1.0678	0.1327	0.1117	0.6558	0.0213	0.0882	0.8834	0.0513	0.0959	0.6658	0.2051	0.0307	0.8196	0.1623	0.0363	0.6430	0.1396	0.0956	1.0276	0.1884	0.0364	0.5748	0.1308	0.1107	0.8070	0.2240	0.1209	0.8633	0.1985	0.1490	0.8838	0.2226	0.0377	0.6962	0.1132	0.1047	0.7715	0.1805	0.2871	0.6989	0.1259	0.2786	0.5631	0.3770
0.0308	0.5773	0.1956	0.0090	0.0635	0.0283	0.0000	0.3008	0.0610	0.0227	0.3974	0.0936	0.0301	0.5820	0.1140	0.0041	0.3504	0.0747	0.0936	1.1778	0.1127	0.0993	0.8020	0.2193	0.0365	0.4855	0.1189	0.0435	0.8137	0.2553	0.0439	0.3961	0.2113	0.0200	0.3279	0.0548	0.0460	0.5063	0.2029	0.0334	0.4382	0.1264	0.0508	0.3579	0.3161
0.0139	0.8029	0.1939	0.0090	0.0974	0.0284	0.0000	0.2254	0.0759	0.0181	0.3585	0.0848	0.0381	0.6768	0.1144	0.0061	0.3970	0.0837	0.0928	1.0009	0.1291	0.0779	0.9303	0.2160	0.0377	0.4632	0.1153	0.0434	0.7056	0.2400	0.0458	0.4587	0.1894	0.0200	0.3525	0.0794	0.0475	0.5154	0.1949	0.0372	0.3909	0.1247	0.0553	0.3721	0.3968
0.0175	0.6068	0.2205	0.0090	0.0974	0.0141	0.0062	0.2515	0.0610	0.0371	0.4468	0.1135	0.0400	0.5749	0.1659	0.0061	0.3504	0.0777	0.0973	1.0166	0.1184	0.0752	0.7686	0.1884	0.0709	0.4955	0.1501	0.0620	0.7578	0.2231	0.0715	0.4120	0.1903	0.1704	0.3591	0.1219	0.0804	0.5171	0.1888	0.0334	0.4624	0.2628	0.1433	0.3621	0.3944
0.0175	0.6252	0.0684	0.0090	0.0311	0.0213	0.0000	0.3529	0.0610	0.0239	0.3886	0.0946	0.0323	0.6892	0.1211	0.0041	0.4451	0.0853	0.0928	1.0009	0.1068	0.0938	0.9211	0.2203	0.0373	0.5402	0.1266	0.0393	0.8668	0.2228	0.0429	0.5066	0.1793	0.0200	0.4042	0.1262	0.0440	0.5517	0.1942	0.0372	0.4135	0.3194	0.0571	0.3800	0.1660
0.1080	0.5773	0.0301	0.0090	0.0635	0.0213	0.0000	0.2748	0.1329	0.0228	0.3710	0.0897	0.0323	0.6704	0.1135	0.7830	0.8006	0.6684	0.0908	1.1430	0.1097	0.0939	0.9157	0.2228	0.0373	0.5008	0.1217	0.0435	0.8137	0.1785	0.0429	0.3963	0.2020	0.0200	0.3525	0.1675	0.0460	0.5012	0.1505	0.0334	0.4382	0.1783	0.0517	0.3590	0.2219
0.0648	0.5773	0.2018	0.0090	0.0635	0.1299	0.0000	0.2748	0.2426	0.0227	0.3909	0.0926	0.0766	0.5865	0.1164	0.0041	0.3504	0.0762	0.0763	0.9182	0.1112	0.0080	0.1410	0.0324	0.0373	0.4754	0.1181	0.0435	0.8557	0.2227	0.0439	0.4013	0.0118	0.0200	0.3525	0.0587	0.0460	0.4920	0.1753	0.0334	0.4382	0.3660	0.0508	0.3450	0.1554
0.0589	0.8637	0.2103	0.0560	0.4439	0.1126	0.0282	0.5729	0.1115	0.0662	0.6796	0.1743	0.0797	0.5655	0.1590	0.0998	0.7314	0.2104	0.0787	1.2186	0.1736	0.0882	0.7345	0.2060	0.1225	0.7250	0.2253	0.0850	0.9698	0.1845	0.0778	0.7123	0.2215	0.0350	0.6435	0.0426	0.0970	0.6936	0.1868	0.1634	0.7819	0.3687	0.3048	0.5417	0.3567
0.0898	0.8211	0.2137	0.0658	0.2946	0.1369	0.0610	1.4550	0.1943	0.0972	0.8355	0.2196	0.0329	0.5777	0.1768	0.0360	0.8477	0.1643	0.0976	0.9360	0.2172	0.1051	0.7891	0.2274	0.1049	0.7683	0.2125	0.1098	1.5814	0.1846	0.0726	0.8730	0.2186	0.0225	0.4207	0.1310	0.1098	0.9207	0.1744	0.2180	0.8040	0.2673	0.1430	0.5028	0.1464
0.0898	0.6727	0.2177	0.0658	0.4439	0.2560	0.0444	0.3543	0.1946	0.0619	0.5629	0.1529	0.0346	0.5903	0.1631	0.0363	0.6600	0.1450	0.0985	1.0273	0.1866	0.0990	0.8063	0.2252	0.0800	0.6212	0.1731	0.1048	1.2140	0.0670	0.0924	0.8638	0.2342	0.0174	0.4503	0.1974	0.1078	0.8158	0.1824	0.0746	0.7982	0.3695	0.1063	0.4564	0.1531
0.0291	0.8472	0.1952	0.1265	1.0169	0.2560	0.0378	1.0692	0.0564	0.0458	0.7118	0.1571	0.0257	0.6826	0.2048	0.1320	0.9989	0.2567	0.0839	0.8605	0.2056	0.0845	0.6617	0.1939	0.0988	0.6932	0.2011	0.1180	1.0397	0.2171	0.0945	0.6923	0.2427	0.0301	0.4632	0.1492	0.1099	0.7674	0.1490	0.2690	0.8174	0.2888	0.2227	0.6169	0.2180
0.0573	0.8297	0.2490	0.1265	1.0169	0.0582	0.0379	1.0554	0.1115	0.0472	0.7719	0.1646	0.0296	0.5833	0.2047	0.1258	1.1023	0.2588	0.0837	0.8691	0.2155	0.0681	0.9901	0.2082	0.0957	0.6965	0.1996	0.1223	0.9957	0.1659	0.0945	0.6617	0.2562	0.0301	0.4346	0.1630	0.1075	0.7965	0.2155	0.2694	0.8506	0.2636	0.2263	0.5846	0.1598
0.1064	0.4748	0.2036	0.0183	0.2070	0.0730	0.0000	0.2488	0.1275	0.0062	0.4840	0.0924	0.0048	0.2093	0.0323	0.0020	0.0217	0.0081	0.0081	0.1692	0.0062	0.0535	0.6433	0.1532	0.0033	0.0154	0.0060	0.0318	0.0564	0.2228	0.0018	0.0094	0.2455	0.0000	0.0159	0.1265	0.0028	0.0310	0.1906	0.0073	0.0000	0.0641	0.0770	0.2102	0.1456
0.0889	0.7155	0.2209	0.0464	0.1702	0.1299	0.0379	0.3846	0.2063	0.0759	0.7319	0.1829	0.0048	0.2046	0.1424	0.0998	0.6240	0.1964	0.0744	0.8266	0.1596	0.0535	0.6953	0.1621	0.0838	0.6332	0.1809	0.0818	0.8828	0.1539	0.0492	0.5812	0.2332	0.0124	0.2915	0.1220	0.0459	0.6843	0.1914	0.0801	0.4935	0.3294	0.1030	0.4133	0.1465
0.1040	0.9550	0.1936	0.0560	0.4439	0.2255	0.0282	0.5356	0.1330	0.0671	0.6193	0.1636	0.0048	0.2235	0.1678	0.0591	0.8727	0.1929	0.0816	1.0406	0.1879	0.0815	0.7169	0.1975	0.1121	0.5331	0.1924	0.0816	0.5902	0.1568	0.0500	0.5373	0.2323	0.0224	0.7410	0.2568	0.0699	0.6542	0.1017	0.1564	0.5471	0.2128	0.2938	0.5511	0.3836
0.1067	0.9459	0.1936	0.1171	0.7662	0.1294	0.0747	0.7184	0.1492	0.0983	0.6640	0.1987	0.0048	0.2467	0.1713	0.0608	0.7287	0.1714	0.0665	0.6427	0.2273	0.0186	0.5210	0.1101	0.0888	0.5910	0.1832	0.1203	0.6538	0.1814	0.1183	0.6819	0.2270	0.0274	0.7410	0.1177	0.0802	0.7144	0.1005	0.2693	0.6432	0.4550	0.2440	0.4817	0.2183
0.0573	0.8126	0.0727	0.0658	0.3908	0.1299	0.0282	0.5729	0.1603	0.0662	0.6608	0.1720	0.0619	0.2649	0.1362	0.0968	0.6260	0.1913	0.0791	0.7034	0.1521	0.0207	0.5437	0.1196	0.1130	0.5539	0.1961	0.0818	0.7995	0.1756	0.0520	0.5175	0.2183	0.0299	0.6092	0.1220	0.0773	0.6453	0.1372	0.1622	0.5397	0.2315	0.3157	0.5337	0.1525
0.0641	0.9935	0.2138	0.0560	0.4439	0.1050	0.0282	0.6973	0.2553	0.0733	0.6930	0.1778	0.0092	0.5230	0.1555	0.0392	0.7127	0.1525	0.0736	0.7659	0.2040	0.0583	0.5532	0.1509	0.1108	0.5682	0.1968	0.0774	0.7272	0.1540	0.0511	0.6303	0.2430	0.0224	0.8320	0.1220	0.0704	0.7579	0.1123	0.1638	0.7122	0.2268	0.2974	0.5553	0.2938
0.0554	0.7132	0.2240	0.0559	0.2965	0.0963	0.0379	0.7131	0.1167	0.0728	0.6845	0.1747	0.0086	0.5084	0.1295	0.0175	0.6307	0.1173	0.0664	0.6855	0.1402	0.0274	0.6127	0.1348	0.0972	0.6148	0.1907	0.0775	0.7214	0.1570	0.0491	0.6428	0.2106	0.0124	0.3190	0.0587	0.0573	0.6143	0.1023	0.0840	0.5833	0.2284	0.2160	0.4394	0.2891
0.0879	0.9495	0.2140	0.0561	0.2493	0.1369	0.0780	1.3844	0.2427	0.0983	0.8814	0.2254	0.0097	0.5641	0.1358	0.0155	0.6414	0.1156	0.0665	0.6835	0.1586	0.0186	0.5300	0.1112	0.0943	0.6400	0.1904	0.1146	1.1064	0.1845	0.0647	0.8155	0.2115	0.0509	0.5238	0.1176	0.0929	0.9310	0.0992	0.2172	1.0037	0.2326	0.1482	0.5336	0.4507
0.0192	0.6724	0.2123	0.0658	0.4439	0.2069	0.0444	0.3831	0.0709	0.0607	0.6035	0.1571	0.0070	0.4624	0.1425	0.1039	0.6757	0.2035	0.0736	0.7247	0.1704	0.0186	0.5300	0.1111	0.0663	0.5674	0.1551	0.1029	1.0862	0.2043	0.0759	0.6633	0.0245	0.0174	0.5120	0.1400	0.0819	0.7885	0.1715	0.0744	0.7340	0.2315	0.1087	0.4497	0.1229
0.0455	1.0467	0.2188	0.1117	0.6558	0.0213	0.0882	0.9429	0.0561	0.0940	0.6520	0.2016	0.0092	0.7121	0.1410	0.0377	0.5225	0.1211	0.0631	0.7013	0.1581	0.0867	0.7005	0.2047	0.0970	0.6293	0.1960	0.1144	0.7760	0.1659	0.1372	0.6575	0.0286	0.0274	0.7410	0.1444	0.0855	0.7384	0.2067	0.2705	0.6124	0.2315	0.2776	0.5521	0.1042
0.0326	0.5540	0.2222	0.0090	0.0635	0.0283	0.0000	0.3277	0.0660	0.0216	0.3974	0.0926	0.0075	0.5010	0.0906	0.0041	0.3493	0.0689	0.0742	0.9060	0.0953	0.0669	1.0776	0.2144	0.0341	0.4172	0.1063	0.0399	0.5807	0.1944	0.0224	0.3358	0.0627	0.0049	0.1757	0.1536	0.0160	0.5100	0.1911	0.0239	0.5334	0.3084	0.0525	0.3549	0.4267
0.0157	0.8211	0.1774	0.0090	0.0974	0.0284	0.0000	0.2497	0.0810	0.0170	0.3401	0.0809	0.0426	0.9015	0.1029	0.0061	0.4589	0.0822	0.0605	0.7241	0.1234	0.0687	0.6515	0.1698	0.0347	0.4232	0.1076	0.0398	0.4979	0.1940	0.0247	0.3806	0.0048	0.0049	0.1958	0.1534	0.0184	0.4899	0.1916	0.0277	0.4069	0.2971	0.0564	0.3724	0.3878
0.0192	0.5826	0.1725	0.0090	0.0974	0.0141	0.0062	0.2768	0.0660	0.0359	0.4468	0.1125	0.0439	0.8544	0.1434	0.0061	0.3380	0.0674	0.0639	0.6997	0.1009	0.0812	0.7288	0.1985	0.0662	0.4095	0.1334	0.0582	0.6300	0.1812	0.0515	0.3688	0.0028	0.1520	0.2407	0.1632	0.0517	0.5414	0.1936	0.0239	0.5612	0.2128	0.1452	0.3455	0.3618
0.0192	0.6005	0.1838	0.0090	0.0311	0.0213	0.0000	0.3817	0.0660	0.0228	0.3822	0.0925	0.0379	0.5852	0.0974	0.0041	0.4184	0.0735	0.0605	0.7103	0.1026	0.0813	0.7370	0.1997	0.0356	0.4677	0.1207	0.0439	0.7218	0.0601	0.0178	0.4543	0.1498	0.0049	0.2594	0.1177	0.0174	0.5671	0.1607	0.0277	0.3842	0.3280	0.0605	0.3804	0.4735
0.1060	0.5660	0.0308	0.0090	0.0635	0.0213	0.0000	0.3008	0.1384	0.0216	0.3710	0.0887	0.0379	0.6656	0.0917	0.7931	0.8499	0.6651	0.0648	0.6301	0.0980	0.0959	0.7944	0.2178	0.0349	0.4172	0.1071	0.0399	0.5807	0.0422	0.0214	0.3312	0.1756	0.0049	0.1958	0.1589	0.0160	0.5235	0.1674	0.0239	0.5334	0.3161	0.0534	0.3560	0.3702
0.0629	0.5660	0.2158	0.0090	0.0635	0.1299	0.0000	0.3008	0.2489	0.0216	0.3909	0.0916	0.0775	0.7090	0.0957	0.0041	0.3380	0.0659	0.0566	0.9107	0.0939	0.1056	0.8795	0.2362	0.0349	0.4080	0.1056	0.0399	0.6117	0.1507	0.0224	0.3358	0.1925	0.0049	0.1958	0.1226	0.0160	0.5141	0.1609	0.0239	0.5334	0.3677	0.0524	0.3420	0.3879
0.0607	0.8473	0.2390	0.0560	0.4439	0.1126	0.0282	0.6121	0.1167	0.0662	0.6796	0.1742	0.0800	0.7063	0.1381	0.0975	0.5907	0.1929	0.0618	0.7796	0.1611	0.1027	0.8820	0.2351	0.1139	0.5477	0.1957	0.0818	0.8868	0.2137	0.0520	0.5385	0.1937	0.0299	0.6092	0.0190	0.0751	0.6231	0.1761	0.1667	0.5678	0.0682	0.3071	0.5553	0.4467
0.0917	0.8397	0.2296	0.0658	0.2946	0.1369	0.0610	1.5887	0.1885	0.0985	0.8592	0.2230	0.0362	0.5731	0.1634	0.0318	0.8010	0.1510	0.0796	0.7441	0.1942	0.0921	0.8151	0.2154	0.0937	0.6751	0.1927	0.1166	1.1569	0.1944	0.0587	0.9038	0.1900	0.0275	0.3688	0.1231	0.0854	0.9138	0.0612	0.2126	0.7405	0.0459	0.1468	0.5275	0.3772
0.0917	0.6463	0.2158	0.0658	0.4439	0.2560	0.0444	0.3831	0.1887	0.0607	0.6047	0.1571	0.0402	0.5479	0.1410	0.0377	0.5369	0.1222	0.0813	0.7759	0.1596	0.0724	1.0973	0.2190	0.0663	0.5846	0.1573	0.1029	1.1485	0.1811	0.0759	0.7082	0.0508	0.0174	0.5120	0.1278	0.0828	0.7823	0.1854	0.0704	0.6377	0.2561	0.1075	0.4492	0.3674
0.0714	0.9232	0.2142	0.1265	1.0169	0.2560	0.0378	0.9986	0.1382	0.0446	0.7519	0.1603	0.0086	0.4569	0.1882	0.1374	0.9635	0.2584	0.0757	0.7635	0.1989	0.0585	0.7753	0.1719	0.0808	0.5667	0.1739	0.1182	0.8638	0.2152	0.0840	0.5977	0.0694	0.0352	0.5258	0.2737	0.0888	0.7296	0.1593	0.2648	0.6115	0.3597	0.2252	0.6284	0.3713
0.1198	0.9039	0.2142	0.1265	1.0169	0.1126	0.0379	0.9864	0.1435	0.0460	0.8155	0.1678	0.0152	0.4719	0.1882	0.1312	0.9531	0.2527	0.0755	0.7127	0.2054	0.0585	0.7977	0.1759	0.0795	0.5695	0.1739	0.1225	0.8715	0.2093	0.0840	0.6117	0.0259	0.0352	0.4947	0.1419	0.0879	0.7512	0.1808	0.2652	0.6377	0.2818	0.2289	0.5956	0.1120
0.1017	0.8325	0.2239	0.0465	0.3882	0.1290	0.0400	0.5370	0.2309	0.0765	0.8684	0.1985	0.0000	0.0252	0.1248	0.1022	0.6407	0.1981	0.0755	0.8200	0.1627	0.0892	0.9129	0.2216	0.0819	0.5975	0.1747	0.0554	0.8942	0.2289	0.0512	0.5749	0.0259	0.0124	0.2689	0.1278	0.0462	0.6654	0.1276	0.0843	0.4904	0.3767	0.1234	0.4810	0.3810
0.1174	0.8803	0.1807	0.0561	0.4408	0.2352	0.0282	0.6535	0.1491	0.0735	0.7921	0.1902	0.0000	0.0107	0.1608	0.0613	0.8964	0.1947	0.0828	0.8635	0.1862	0.0268	0.6370	0.1293	0.1099	0.5348	0.1908	0.0677	0.5378	0.2225	0.0520	0.5313	0.1566	0.0224	0.6993	0.1278	0.0699	0.6441	0.1294	0.1477	0.5436	0.2614	0.3086	0.6498	0.3605
0.1191	0.9729	0.1904	0.1173	0.8467	0.1287	0.0782	0.8972	0.1714	0.1018	0.8174	0.2197	0.0070	0.1943	0.1584	0.0630	0.7098	0.1698	0.0669	0.5362	0.2273	0.0288	0.6113	0.1336	0.0893	0.5697	0.1866	0.0998	0.6741	0.2155	0.1205	0.6903	0.1860	0.0274	0.6993	0.1534	0.0801	0.7036	0.1677	0.2645	0.6388	0.2501	0.2644	0.5590	0.4140
0.0714	0.7661	0.1690	0.0659	0.3882	0.1290	0.0282	0.6973	0.1770	0.0726	0.8211	0.1964	0.0573	0.0572	0.1308	0.0992	0.6426	0.1983	0.0750	0.6315	0.1521	0.0669	0.6519	0.1690	0.1112	0.5363	0.1921	0.0678	0.8099	0.2187	0.0540	0.5242	0.2007	0.0299	0.5739	0.1183	0.0772	0.6190	0.1406	0.1535	0.5362	0.2613	0.3199	0.6768	0.4086
0.0745	0.9247	0.1673	0.0561	0.4408	0.1294	0.0282	0.9037	0.2489	0.0804	0.8088	0.1979	0.0097	0.4321	0.1516	0.0414	0.7315	0.1559	0.0660	0.6615	0.2008	0.0357	0.6761	0.1481	0.1090	0.5727	0.1960	0.0635	0.6645	0.0628	0.0530	0.6164	0.1999	0.0224	0.7851	0.1224	0.0703	0.7401	0.1282	0.1551	0.7069	0.2614	0.3104	0.6653	0.4815
0.0771	0.7799	0.0783	0.0560	0.4439	0.1294	0.0400	0.8464	0.1327	0.0747	0.8345	0.1935	0.0113	0.4440	0.1220	0.0196	0.6475	0.1204	0.0668	0.5390	0.1402	0.0268	0.6266	0.1283	0.0953	0.5915	0.1859	0.0513	0.7309	0.1976	0.0511	0.6360	0.1953	0.0124	0.2953	0.1490	0.0573	0.6154	0.1248	0.0881	0.5793	0.2636	0.2159	0.4863	0.4783
0.1006	1.2224	0.2122	0.0776	0.3290	0.1369	0.0814	1.2339	0.2616	0.1038	0.9953	0.2420	0.0146	0.4883	0.1259	0.0176	0.6414	0.1172	0.0669	0.5376	0.1586	0.0268	0.6266	0.1283	0.0923	0.6279	0.1872	0.0870	1.0610	0.1782	0.0668	0.8255	0.0904	0.0509	0.4929	0.1489	0.0937	0.9091	0.1618	0.2199	1.0202	0.2613	0.1630	0.5532	0.3481
0.0335	0.7509	0.2135	0.0659	0.4408	0.2163	0.0466	0.4660	0.0861	0.0671	0.7304	0.1796	0.0097	0.3723	0.1348	0.1062	0.6935	0.2071	0.0739	0.6967	0.1704	0.0943	0.7954	0.2203	0.0655	0.5551	0.1528	0.0764	1.0905	0.1810	0.0789	0.6564	0.0315	0.0174	0.4814	0.0506	0.0818	0.7768	0.1705	0.0786	0.7285	0.0718	0.1312	0.5087	0.3372
0.0650	1.0775	0.2275	0.1119	0.7257	0.0356	0.0917	1.1161	0.0808	0.0994	0.7550	0.2191	0.0119	0.6290	0.1333	0.0399	0.5666	0.1284	0.0670	0.6114	0.1596	0.0684	1.0593	0.2146	0.0975	0.6250	0.2017	0.0911	0.8713	0.1808	0.1394	0.6655	0.0278	0.0274	0.6993	0.1130	0.0855	0.7029	0.1619	0.2657	0.6082	0.3543	0.3029	0.6039	0.3189
0.0472	0.5291	0.2004	0.0091	0.1322	0.0503	0.0000	0.4148	0.0911	0.0268	0.5438	0.1185	0.0124	0.4501	0.0849	0.0061	0.3608	0.0748	0.0740	0.6802	0.0939	0.0708	0.7841	0.1847	0.0323	0.3903	0.1007	0.0155	0.4975	0.1839	0.0224	0.3409	0.1794	0.0049	0.1562	0.1683	0.0160	0.4971	0.1650	0.0314	0.5299	0.2795	0.0720	0.4145	0.1691
0.0300	0.7455	0.2113	0.0091	0.2082	0.0429	0.0000	0.3846	0.0810	0.0222	0.4819	0.1074	0.0460	0.9352	0.0911	0.0082	0.4722	0.0884	0.0644	0.6315	0.1234	0.0878	0.8657	0.2166	0.0331	0.4041	0.1033	0.0154	0.4739	0.1905	0.0247	0.3861	0.2029	0.0049	0.1757	0.1444	0.0184	0.4728	0.1740	0.0353	0.4045	0.3339	0.0735	0.4271	0.3213
0.0335	0.5450	0.2546	0.0091	0.1693	0.0430	0.0062	0.4180	0.0811	0.0412	0.5864	0.1369	0.0479	0.8049	0.1363	0.0082	0.3493	0.0733	0.0599	0.6156	0.0981	0.0879	0.8608	0.2166	0.0643	0.3827	0.1276	0.0332	0.5412	0.1998	0.0515	0.3742	0.2245	0.1520	0.2631	0.1398	0.0517	0.5280	0.1789	0.0314	0.5575	0.3395	0.1593	0.4310	0.3288

0.0335	0.5270	0.2180
0.1194	0.5291	0.2305
0.0753	0.5291	0.2096
0.0819	0.7822	0.2096
0.1032	0.8999	0.0128
0.1032	0.7575	0.2154
0.0953	0.9428	0.2311
0.0627	0.9428	0.2056
0.0904	0.5921	0.2039
0.0943	0.7752	0.2086
0.0497	0.6133	0.2240
0.0370	0.6517	0.2137
0.0617	0.1663	0.2108
0.0452	0.7155	0.2054
0.0645	0.5522	0.2471
0.0618	0.8196	0.2138
0.0477	0.4039	0.2192
0.0452	0.8125	0.2037
0.0452	0.4128	0.2037
0.0923	0.6932	0.0798
0.0562	0.3942	0.2294
0.0430	0.3942	0.2223
0.0777	0.6133	0.2246
0.0777	0.7311	0.2228
0.1008	0.5474	0.1663
0.0034	0.7643	0.1394
0.0017	0.7485	0.1755
0.0963	0.8168	0.1377
0.0929	0.2066	0.1889
0.1031	0.1402	0.1500
0.0998	0.6497	0.1696
0.0990	0.7695	0.1362
0.1217	0.7164	0.1362
0.1076	0.8283	0.2105
0.0981	0.5791	0.1663
0.0990	0.9019	0.1424
0.0990	0.5857	0.2007
0.0052	0.8154	0.2008
0.0912	0.5667	0.1517
0.1048	0.5667	0.1583
0.1053	0.2221	0.1566
0.1043	0.8111	0.1822
0.1001	0.7970	0.1902
0.1018	0.7197	0.2398
0.0627	0.7122	0.2263
0.0497	0.6830	0.2360

0.0091	0.1693	0.0356
0.0091	0.1322	0.0356
0.0091	0.1322	0.1290
0.0561	0.4408	0.1294
0.0675	0.3798	0.1369
0.0659	0.4408	0.3193
0.1537	1.6031	0.3193
0.1537	1.6031	0.1126
0.0274	0.5047	0.2066
0.0910	0.8165	0.1042
0.0368	0.3934	0.1044
0.0274	0.4470	0.0286
0.0090	0.0985	0.0972
0.0658	0.2094	0.1287
0.0511	0.4753	0.1976
0.0858	0.7798	0.0730
0.0368	0.2107	0.0730
0.0368	0.2107	0.0806
0.0368	0.2524	0.0653
0.0368	0.1712	0.0730
0.0368	0.2107	0.0730
0.0368	0.2107	0.1044
0.0274	0.4470	0.0811
0.0559	0.1712	0.1287
0.0511	0.4753	0.2180
0.1158	0.7292	0.2180
0.1158	0.7292	0.2836
0.1169	1.6184	0.0284
0.0090	0.0980	0.0141
0.0000	0.0639	0.1126
0.0415	0.4226	0.1635
0.0707	0.5959	0.1463
0.0415	0.6721	0.2539
0.1115	1.1629	0.1046
0.0463	0.3434	0.1206
0.0463	0.4470	0.1126
0.0463	0.3934	0.1129
0.0463	0.3934	0.1046
0.0463	0.3434	0.1046
0.0463	0.3434	0.0286
0.0000	0.1337	0.1635
0.0608	0.6721	0.1463
0.0415	0.6721	0.2753
0.1263	1.3246	0.2753
0.1263	1.3246	0.2638
0.1275	1.0824	0.2636

0.0000	0.3817	0.0811
0.0000	0.3846	0.1546
0.0000	0.3846	0.2618
0.0282	0.7438	0.1327
0.0643	1.8484	0.2061
0.0466	0.4660	0.2181
0.0410	1.1895	0.1063
0.0411	1.3640	0.2876
0.0410	0.3454	0.1273
0.0978	1.3912	0.1221
0.0410	0.4681	0.0860
0.0410	0.4355	0.2743
0.0000	0.4164	0.0909
0.0882	1.5887	0.2614
0.0250	0.3277	0.0858
0.1045	0.9913	0.0910
0.0443	0.2262	0.0909
0.0443	0.2506	0.1273
0.0508	0.2278	0.0909
0.0444	0.4444	0.0909
0.0443	0.2506	0.1115
0.0443	0.2506	0.2302
0.0410	0.3742	0.0909
0.0710	1.1237	0.1943
0.0250	0.3277	0.2002
0.0475	0.9715	0.2377
0.0476	1.0265	0.0324
0.0510	1.2908	0.0229
0.0000	0.1376	0.1327
0.0000	0.0957	0.2121
0.0410	0.5022	0.1327
0.0476	1.1725	0.1886
0.0541	0.4338	0.1118
0.0575	0.7166	0.1274
0.0313	0.4197	0.1116
0.0313	0.5199	0.1274
0.0377	0.3922	0.1116
0.0314	0.5154	0.1116
0.0313	0.4197	0.0184
0.0313	0.4197	0.2064
0.0000	0.0755	0.1327
0.0314	1.3220	0.1327
0.0541	0.4338	0.1327
0.0061	0.7311	0.2066
0.0062	0.7238	0.2187
0.0510	0.9126	0.3144

0.0280	0.4725	0.1114
0.0268	0.5271	0.1164
0.0268	0.5359	0.1174
0.0726	0.8211	0.1964
0.1026	0.9162	0.2335
0.0670	0.7219	0.1784
0.0516	0.9635	0.1879
0.0530	0.9490	0.1879
0.0652	0.7038	0.1700
0.1152	0.9065	0.2401
0.0643	0.7401	0.1783
0.0689	0.7548	0.1787
0.0124	0.2818	0.0651
0.0954	0.8201	0.2166
0.0735	0.6393	0.1722
0.1154	0.9773	0.2530
0.0753	0.6118	0.1696
0.0733	0.7725	0.1861
0.0914	0.6005	0.1827
0.0761	0.6852	0.1807
0.0754	0.6373	0.1729
0.0753	0.6031	0.1686
0.0643	0.7937	0.1838
0.0936	0.8278	0.2154
0.0735	0.6227	0.1700
0.0808	0.8052	0.1966
0.0837	0.8024	0.1989
0.1043	0.5045	0.1785
0.0022	0.1630	0.0363
0.0056	0.2078	0.0464
0.0607	0.4723	0.1370
0.1125	0.5842	0.1987
0.0734	0.5709	0.1616
0.0997	0.5498	0.1852
0.0697	0.6170	0.1660
0.0673	0.5730	0.1572
0.0850	0.6364	0.1824
0.0683	0.5559	0.1577
0.0697	0.5730	0.1605
0.0697	0.5910	0.1627
0.0022	0.1678	0.0373
0.1092	0.5496	0.1908
0.0734	0.5476	0.1584
0.0581	0.5481	0.1475
0.0568	0.5646	0.1485
0.1057	0.4888	0.1836

0.0385	0.5651	0.0903
0.0385	0.6186	0.0853
0.0782	0.6764	0.0876
0.0830	0.7004	0.1255
0.0391	0.5387	0.1532
0.0407	0.4869	0.1340
0.0114	0.4214	0.1750
0.0179	0.3986	0.1775
0.0000	0.0216	0.1801
0.0070	0.1807	0.1758
0.0573	0.0847	0.0494
0.0097	0.4447	0.1662
0.0113	0.4377	0.1281
0.0146	0.4815	0.1334
0.0097	0.3840	0.0508
0.0119	0.6210	0.1280
0.0124	0.4437	0.1245
0.0460	0.8879	0.1371
0.0479	0.7947	0.1719
0.0385	0.5428	0.1278
0.0385	0.5869	0.1225
0.0782	0.6852	0.1237
0.0830	0.6914	0.0157
0.0391	0.5102	0.1593
0.0407	0.4735	0.1317
0.0114	0.4276	0.1731
0.0179	0.3926	0.1723
0.0070	0.1989	0.0505
0.0573	0.0688	0.1606
0.0097	0.4258	0.1743
0.0113	0.4377	0.1665
0.0146	0.4815	0.1760
0.0097	0.3665	0.1662
0.0119	0.6290	0.1475
0.0124	0.4437	0.1798
0.0460	0.9352	0.1739
0.0479	0.8049	0.2230
0.0385	0.5576	0.1774
0.0385	0.6186	0.1807
0.0782	0.6678	0.1859
0.0830	0.7004	0.1722
0.0391	0.5387	0.1742
0.0407	0.5006	0.1460
0.0114	0.4214	0.1937
0.0179	0.3986	0.1921
0.0649	0.2609	0.1544

0.0062	0.4309	0.0795
0.8020	0.8847	0.6819
0.0061	0.3493	0.0718
0.0999	0.6066	0.1964
0.0339	0.7410	0.1476
0.0399	0.5818	0.1286
0.1398	1.0445	0.2681
0.1336	1.0330	0.2624
0.1221	0.6465	0.2214
0.1221	0.6452	0.2205
0.0041	0.2527	0.0518
0.1145	0.7872	0.2343
0.1056	0.6193	0.1964
0.1046	0.6383	0.1964
0.0061	0.1946	0.0417
0.1153	0.7707	0.2237
0.0963	0.5861	0.1948
0.0986	0.7409	0.2108
0.0986	0.5706	0.1930
0.1034	0.6467	0.2036
0.8497	0.7988	0.7428
0.0963	0.5706	0.1913
0.0020	0.1330	0.0304
0.1074	0.7473	0.2179
0.1153	0.7909	0.2306
0.1750	0.9183	0.2990
0.1698	0.9705	0.2949
0.0081	0.2520	0.0629
0.1186	0.6012	0.2175
0.0668	0.7781	0.1808
0.0624	0.6306	0.1724
0.0581	0.7920	0.1829
0.1222	0.5608	0.2123
0.0778	0.8782	0.1935
0.0591	0.7479	0.1860
0.0635	0.8747	0.1981
0.0613	0.7679	0.1877
0.0621	1.2023	0.2141
0.8174	1.0296	0.7819
0.0591	0.7679	0.1860
0.1209	0.6367	0.2211
0.0591	0.7679	0.1725
0.0778	0.8782	0.2016
0.1496	0.8222	0.2701
0.1433	0.9534	0.2761
0.1175	0.5763	0.2060

0.0644	0.6191	0.1068
0.0590	0.5528	0.0966
0.0612	0.8857	0.0925
0.0664	0.7150	0.1642
0.0825	0.7097	0.1942
0.0843	0.7695	0.1611
0.0762	0.7188	0.2005
0.0761	0.6974	0.2070
0.0231	0.2899	0.1878
0.0722	0.8550	0.1975
0.0788	1.0181	0.2205
0.0796	1.1519	0.2073
0.0727	0.8387	0.2012
0.0727	0.8360	0.2112
0.0787	1.0747	0.2027
0.0705	0.8135	0.2141
0.0642	1.0504	0.2059
0.0678	0.8240	0.2012
0.0722	0.8359	0.2073
0.0678	0.8240	0.0786
0.0722	0.8888	0.2007
0.0589	0.8495	0.2271
0.0589	0.9021	0.2373
0.0806	0.9091	0.0709
0.0823	1.0072	0.1846
0.0668	0.7766	0.1596
0.0667	0.7991	0.1195
0.0743	0.8800	0.1469
0.0804	0.9745	0.1166
0.0812	1.1225	0.1387
0.0748	0.9152	0.1223
0.0749	0.8773	0.1180
0.0803	1.1635	0.1705
0.0688	0.9429	0.1832
0.0666	1.0784	0.1459
0.0661	0.9555	0.2170
0.0705	0.9701	0.2220
0.0661	0.9555	0.1767
0.0740	1.0338	0.1895
0.0589	0.8945	0.1436
0.0589	1.0087	0.1483
0.0931	0.9797	0.1405
0.0949	1.0880	0.1493
0.0776	0.9178	0.1465
0.0775	0.8578	0.1421
0.0188	0.3566	0.1736

0.1049	0.7501	0.2176
0.1003	0.6399	0.2071
0.0146	0.3221	0.0759
0.0907	0.8596	0.2177
0.0744	0.7845	0.1893
0.0764	0.7281	0.1927
0.0152	0.3323	0.0748
0.0828	0.7160	0.1976
0.0879	0.6650	0.1952
0.1226	0.7477	0.2401
0.0900	0.6512	0.1999
0.0828	0.7160	0.1976
0.0828	0.7160	0.1976
0.0039	0.0810	0.0222
0.0931	0.8976	0.2251
0.0830	0.8571	0.2177
0.0940	0.7421	0.1997
0.0940	0.7505	0.2032
0.0099	0.3103	0.0644
0.1042	0.6429	0.2042
0.1182	0.8386	0.2349
0.0971	0.7636	0.2044
0.0979	0.7423	0.2138
0.1053	0.7718	0.2201
0.1062	0.8715	0.2362
0.1066	0.8128	0.2261
0.1517	0.9601	0.2898
0.1135	0.8911	0.2425
0.1062	0.8861	0.2374
0.1062	0.9160	0.2400
0.1064	0.7635	0.2213
0.1140	0.7999	0.2299
0.1011	0.6785	0.2163
0.1160	0.7312	0.2164
0.1161	0.7275	0.2151
0.0995	0.6044	0.2011
0.1115	0.8073	0.2277
0.0935	0.6236	0.1891
0.0947	0.6137	0.2033
0.0992	0.7759	0.2193
0.1012	0.6337	0.2130
0.1038	0.7024	0.2178
0.1455	0.6830	0.2592
0.1082	0.7407	0.2289
0.1012	0.6659	0.2166
0.1012	0.6443	0.2142

0.0344	0.4499	0.1171
0.0331	0.3903	0.1014
0.0331	0.3814	0.1000
0.1121	0.5302	0.1917
0.0935	0.6623	0.1912
0.0655	0.5720	0.1550
0.0830	0.5628	0.1754
0.0817	0.5544	0.1739
0.1340	0.6117	0.2224
0.1114	0.6160	0.2012
0.1295	0.6459	0.2224
0.1310	0.6005	0.2190
0.0207	0.2149	0.0615
0.1057	0.6443	0.2019
0.0923	0.5273	0.1733
0.1220	0.6789	0.2191
0.0855	0.5482	0.1708
0.0855	0.5430	0.1693
0.1140	0.5814	0.1998
0.0871	0.5567	0.1741
0.0846	0.5537	0.1708
0.0846	0.5537	0.1708
0.1292	0.6409	0.2226
0.1060	0.6168	0.1980
0.0923	0.4959	0.1688
0.1128	0.6170	0.2063
0.1096	0.5963	0.2006
0.1311	0.5741	0.2145
0.0154	0.2180	0.0578
0.0156	0.1790	0.0527
0.1358	0.6130	0.2220
0.1299	0.7315	0.2337
0.1102	0.5394	0.1908
0.1436	0.6489	0.2373
0.1210	0.5805	0.2066
0.1142	0.5276	0.1932
0.1525	0.5583	0.2298
0.1170	0.6586	0.2138
0.1201	0.5921	0.2074
0.1201	0.5805	0.2058
0.0146	0.2001	0.0555
0.1329	0.7072	0.2328
0.1098	0.5258	0.1885
0.1105	0.5867	0.1987
0.1091	0.5838	0.1971
0.1285	0.6189	0.2181

0.0193	0.6890	0.1779
0.0155	0.4975	0.2193
0.0155	0.5251	0.0636
0.0678	0.8983	0.2296
0.0890	1.1080	0.2264
0.0764	1.1530	0.2499
0.0869	0.7829	0.2499
0.0911	0.7894	0.2298
0.0698	0.7152	0.2330
0.0784	1.0721	0.1934
0.0727	0.7597	0.1873
0.0656	0.8347	0.2160
0.0038	0.3320	0.1481
0.0701	0.9012	0.1481
0.0453	0.6987	0.0497
0.0784	1.0155	0.1506
0.0453	0.7321	0.2162
0.0480	0.7506	0.2041
0.0638	0.7931	0.1873
0.0521	0.9752	0.2043
0.0453	0.7321	0.1954
0.0453	0.7701	0.2176
0.0727	0.7597	0.2142
0.0658	0.8240	0.2045
0.0453	0.6640	0.2076
0.0693	0.6747	0.0072
0.0734	0.6799	0.2097
0.0932	0.4802	0.1942
0.0077	0.2918	0.1811
0.0038	0.2134	0.1872
0.0656	0.4954	0.1046
0.0955	0.6378	0.1146
0.0679	0.7631	0.2289
0.0889	0.5076	0.1840
0.0679	1.0962	0.2487
0.0678	0.9935	0.1990
0.0848	0.9887	0.1918
0.0720	1.2508	0.1914
0.0679	1.0962	0.2438
0.0679	1.1594	0.2392
0.0077	0.3130	0.2309
0.0925	0.6339	0.1574
0.0679	0.6545	0.1464
0.0847	0.5341	0.1683
0.0888	0.5378	0.1579
0.0962	0.5419	0.1576

0.0178	0.4603	0.2164
0.0214	0.3363	0.2126
0.0224	0.3409	0.0643
0.0540	0.5454	0.0627
0.0607	0.9151	0.1531
0.0784	0.7207	0.1850
0.0840	0.6052	0.2144
0.0840	0.6052	0.1902
0.0551	0.6329	0.1875
0.1237	0.6396	0.0048
0.0566	0.6001	0.1489
0.0551	0.6824	0.1756
0.0150	0.2216	0.1915
0.0647	0.5696	0.1937
0.0750	0.6631	0.1901
0.1347	0.5573	0.1498
0.0404	0.5505	0.1730
0.0453	0.5943	0.1925
0.0690	0.5559	0.1919
0.0434	0.6223	0.1883
0.0404	0.5446	0.1818
0.0404	0.5378	0.1832
0.0566	0.6381	0.1744
0.0527	0.6532	0.1718
0.0750	0.6547	0.2016
0.0806	0.6441	0.1849
0.0811	0.6119	0.1840
0.1101	0.6513	0.2210
0.0055	0.1760	0.2200
0.0028	0.1562	0.0118
0.0582	0.6017	0.1275
0.0607	0.7840	0.1183
0.0668	0.6579	0.0719
0.1243	0.6284	0.0711
0.0486	0.6030	0.1479
0.0515	0.6215	0.1550
0.0800	0.6120	0.2215
0.0521	0.6694	0.1540
0.0486	0.5965	0.1576
0.0486	0.5960	0.1548
0.0074	0.1886	0.1989
0.0507	0.9092	0.1949
0.0668	0.6643	0.2514
0.0742	0.6111	0.1093
0.0763	0.5698	0.1142
0.1102	0.7198	0.1371

0.0049	0.2376	0.1176
0.0049	0.1757	0.1221
0.0049	0.1757	0.2729
0.0299	0.5739	0.1316
0.0275	0.3435	0.1221
0.0174	0.4814	0.1221
0.0352	0.4947	0.1177
0.0352	0.4648	0.1353
0.0099	0.5969	0.1175
0.0249	0.4682	0.1263
0.0199	0.5339	0.1312
0.0099	0.6706	0.2029
0.0000	0.1182	0.1400
0.0484	0.3639	0.1632
0.0149	0.3290	0.1312
0.0249	0.4984	0.1266
0.0175	0.0908	0.2516
0.0175	0.1085	0.1362
0.1585	0.2384	0.1266
0.0175	0.2462	0.1266
0.0175	0.1085	0.0506
0.0175	0.1085	0.1268
0.0199	0.5339	0.1310
0.0250	0.1557	0.1449
0.0149	0.3290	0.1541
0.0353	0.4892	0.1134
0.0353	0.5199	0.0753
0.0298	0.9964	0.1177
0.0098	0.2491	0.0424
0.0000	0.2037	0.0464
0.0148	0.7231	0.1813
0.0656	0.9173	0.0667
0.0248	0.8160	0.0464
0.0298	0.9375	0.0464
0.0274	0.5842	0.1130
0.0274	0.5503	0.0670
0.1723	0.6373	0.0752
0.0274	0.5179	0.1130
0.0274	0.5503	0.1174
0.0274	0.5503	0.1179
0.0098	0.2491	0.1541
0.0350	0.4752	0.1183
0.0248	0.7263	0.1229
0.0454	0.6067	0.2629
0.0454	0.6822	0.1457
0.0399	0.7466	0.1229

0.0174	0.5532	0.0671
0.0160	0.5011	0.0641
0.0160	0.5011	0.1677
0.0750	0.6081	0.2091
0.0862	0.9001	0.1752
0.0828	0.7707	0.1834
0.0904	0.7310	0.1689
0.0895	0.7527	0.1702
0.0605	0.5985	0.1996
0.0700	0.5733	0.1805
0.0678	0.5903	0.1701
0.0625	0.7253	0.1663
0.0155	0.2240	0.0661
0.0735	0.7218	0.1959
0.0732	0.5283	0.1752
0.0769	0.6465	0.1844
0.0401	0.5209	0.1883
0.0422	0.5214	0.1798
0.0740	0.5327	0.1937
0.0467	0.5689	0.1612
0.0401	0.5251	0.1888
0.0401	0.5156	0.1811
0.0645	0.6303	0.0730
0.0678	0.6444	0.1777
0.0741	0.5379	0.1797
0.0800	0.5459	0.2290
0.0797	0.6160	0.1850
0.0828	0.5572	0.1797
0.0183	0.2215	0.1764
0.0119	0.2340	0.1820
0.0648	0.5937	0.1753
0.0923	0.7908	0.1797
0.0801	0.5900	0.1534
0.0775	0.6282	0.1590
0.0684	0.5996	0.0750
0.0720	0.5853	0.1739
0.1026	0.5874	0.2163
0.0719	0.6611	0.1775
0.0684	0.6149	0.1856
0.0684	0.5936	0.1782
0.0172	0.2331	0.1783
0.0848	0.7306	0.2095
0.0812	0.5836	0.1856
0.0906	0.6060	0.1796
0.0897	0.6415	0.1760
0.0858	0.5938	0.0241

0.0353	0.3820	0.1669
0.0314	0.5299	0.1611
0.0314	0.5299	0.1630
0.1579	0.5640	0.2514
0.2177	0.7349	0.2949
0.0745	0.6333	0.4383
0.2704	0.6074	0.3005
0.2708	0.6333	0.3120
0.1659	0.7002	0.2569
0.2882	0.5971	0.3880
0.1740	0.6747	0.2428
0.1749	0.6040	0.4333
0.0294	0.2758	0.1690
0.1613	0.8274	0.1596
0.0487	0.6880	0.1689
0.3041	0.9401	0.1647
0.0841	0.7783	0.1690
0.0963	0.6377	0.1671
0.0841	0.8173	0.3027
0.0963	0.5774	0.3797
0.0841	0.7783	0.2366
0.0841	0.7783	0.4050
0.1786	0.7086	0.4140
0.1637	0.6593	0.0804
0.0448	0.7246	0.0855
0.2436	0.4550	0.2235
0.2440	0.4283	0.2797
0.3003	0.5338	0.4027
0.0170	0.2999	0.2875
0.0073	0.2351	0.2983
0.1669	0.6023	0.2371
0.2774	0.7840	0.3455
0.1517	1.1355	0.2140
0.2951	0.6559	0.4065
0.1591	0.6192	0.1282
0.1705	0.5726	0.1145
0.1591	0.6192	0.1318
0.1705	0.4908	0.1316
0.1591	0.6192	0.1282
0.1591	0.6192	0.1265
0.0245	0.3203	0.2969
0.2732	0.6676	0.3425
0.1516	1.0830	0.2060
0.2679	0.5121	0.3908
0.2682	0.5355	0.3936
0.3068	0.6768	0.3895

0.0809	0.4449	0.3708
0.0708	0.4145	0.3164
0.0720	0.4008	0.3127
0.3098	0.6908	0.3173
0.1647	0.5526	0.4672
0.1300	0.5002	0.3290
0.2421	0.7176	0.3165
0.2436	0.6573	0.3492
0.3155	0.5251	0.3468
0.2519	0.5245	0.1312
0.3352	0.5152	0.4266
0.3113	0.6175	0.4439
0.1450	0.2147	0.4001
0.1398	0.5091	0.5039
0.0885	0.4144	0.3950
0.2812	0.5984	0.4026
0.0792	0.4193	0.4601
0.0844	0.4384	0.3930
0.1520	0.4552	0.3838
0.0839	0.5168	0.3851
0.0790	0.4205	0.0287
0.0791	0.4197	0.4277
0.3184	0.5180	0.3961
0.1359	0.5032	0.4409
0.0873	0.3998	0.4395
0.2111	0.5959	0.4337
0.2139	0.5520	0.3898
0.3974	0.5077	0.3752
0.0735	0.2593	0.4715
0.0562	0.2195	0.3712
0.3768	0.5128	0.3949
0.3104	0.5983	0.4440
0.3047	0.5179	0.3866
0.4136	0.5162	0.3710
0.2955	0.5941	0.3722
0.2989	0.6809	0.1221
0.3728	0.6189	0.3988
0.2961	0.6562	0.3713
0.2971	0.5759	0.4153
0.2970	0.5948	0.4181
0.0618	0.2612	0.2943
0.3119	0.5561	0.2556
0.3032	0.5174	0.3745
0.3651	0.5491	0.2591
0.3666	0.5008	0.2667
0.4055	0.5192	0.3178

0.0730	0.8254	0.2506	0.1169	1.2223	0.2066	0.0510	1.0419	0.2493	0.1020	0.5336	0.1807	0.0114	0.4652	0.1888	0.0597	0.6670	0.1530	0.0114	0.2968	0.1879	0.1018	0.6406	0.2107	0.1286	0.6377	0.2209	0.0889	0.6619	0.1613	0.1102	0.6942	0.1158	0.0298	0.9375	0.1229	0.0836	0.6865	0.1995	0.2993	0.5659	0.3785	0.4046	0.6208	0.2678
0.0781	0.7109	0.1927	0.1011	0.7382	0.2163	0.0978	2.0715	0.2687	0.1085	0.6576	0.2045	0.0135	0.4811	0.1655	0.0591	0.5420	0.1498	0.0000	0.1154	0.1831	0.1106	0.8746	0.2339	0.1184	0.6069	0.2056	0.0826	0.7861	0.2072	0.1180	0.6453	0.1077	0.0249	0.5381	0.1683	0.0713	0.5428	0.1768	0.2980	0.4442	0.3691	0.3036	0.5192	0.2570
0.1064	0.7018	0.2226	0.1119	0.7257	0.2441	0.0546	1.3931	0.1207	0.1188	0.7519	0.2267	0.0097	0.5238	0.1695	0.0548	0.6152	0.1565	0.0000	0.0967	0.2038	0.0989	0.6431	0.2130	0.1103	0.6732	0.2065	0.0871	0.5686	0.2659	0.1077	0.6645	0.1093	0.0665	0.5693	0.0879	0.0801	0.7506	0.1838	0.3546	0.6347	0.4107	0.2530	0.4505	0.2591
0.0927	0.7058	0.2196	0.1293	0.8469	0.0356	0.0979	1.1066	0.2621	0.1037	0.6121	0.1955	0.0119	0.3881	0.1630	0.1223	0.6071	0.2133	0.0073	0.2702	0.2137	0.1082	0.6446	0.2058	0.0866	0.5943	0.1763	0.0849	1.0848	0.2435	0.1267	0.7261	0.1532	0.0198	0.6092	0.1177	0.0845	0.6605	0.1884	0.2698	0.6982	0.3639	0.2527	0.5072	0.4182
0.0763	0.1321	0.2131	0.0091	0.1301	0.2067	0.0125	0.5716	0.2247	0.0209	0.2164	0.0648	0.0141	0.6176	0.1475	0.0715	0.7261	0.1722	0.0305	0.3730	0.1880	0.1083	0.6309	0.2022	0.0240	0.2299	0.0747	0.0077	0.3761	0.2435	0.0401	0.3840	0.1985	0.0000	0.0858	0.1489	0.0112	0.2520	0.1805	0.0618	0.3200	0.1307	0.0995	0.2515	0.2947
0.0781	0.9691	0.1518	0.1065	0.6929	0.1979	0.0713	1.3728	0.2558	0.0967	0.6927	0.1987	0.0124	0.4741	0.1702	0.0613	0.6199	0.1647	0.0296	0.5152	0.1705	0.0813	0.7975	0.2045	0.0940	0.7199	0.1973	0.0958	1.2428	0.2431	0.1131	0.6612	0.1999	0.0300	0.5339	0.1534	0.0749	0.7020	0.2171	0.2793	0.6798	0.3822	0.2295	0.6382	0.2522
0.0781	0.9684	0.1960	0.1013	0.6558	0.2069	0.0713	0.9023	0.2316	0.0955	0.6629	0.1940	0.0432	0.8327	0.1767	0.0613	0.7065	0.1714	0.0280	0.3524	0.2009	0.0689	0.6761	0.1714	0.0924	0.6854	0.1918	0.0986	1.0235	0.0797	0.1200	0.7581	0.1912	0.0300	0.5671	0.1231	0.0764	0.7076	0.1830	0.2848	0.7107	0.3814	0.2378	0.6610	0.3360
0.1030	0.9757	0.1705	0.1065	0.6929	0.2160	0.0779	1.1983	0.2558	0.1121	0.6893	0.2124	0.0451	0.8257	0.2186	0.0635	0.6034	0.1630	0.0322	0.3493	0.1705	0.0709	0.6923	0.1844	0.1240	0.7117	0.2235	0.1155	1.2141	0.2325	0.1405	0.6582	0.1857	0.1868	0.6632	0.0883	0.1077	0.7307	0.1977	0.2793	0.6798	0.3821	0.3015	0.5992	0.3310
0.0590	1.0059	0.1628	0.1065	0.7730	0.2067	0.0714	0.9516	0.2558	0.0962	0.7020	0.1999	0.0357	0.6533	0.1749	0.0647	0.8554	0.1834	0.0280	0.3439	0.1627	0.0059	0.2156	0.0499	0.0933	0.7414	0.2043	0.1029	1.3743	0.2020	0.1181	0.7829	0.1437	0.0300	0.6764	0.0883	0.0782	0.7221	0.1760	0.2875	0.6939	0.3975	0.2312	0.6340	0.2237
0.0740	0.9485	0.1517	0.1065	0.6929	0.2067	0.0713	1.2709	0.2125	0.0968	0.6447	0.1930	0.0357	0.6866	0.1734	0.8194	1.1798	0.7827	0.0263	0.4451	0.1705	0.0735	0.5812	0.1729	0.0932	0.7199	0.1966	0.0958	1.2428	0.2392	0.1142	0.6470	0.1229	0.0300	0.5671	0.2162	0.0749	0.7133	0.1766	0.2793	0.6477	0.3819	0.2237	0.6480	0.3796
0.0776	0.9485	0.1517	0.1065	0.6929	0.2539	0.0713	1.2709	0.2370	0.0967	0.7024	0.1999	0.0800	0.6683	0.1813	0.0613	0.6034	0.1614	0.0415	0.6444	0.1690	0.0786	0.6266	0.1822	0.0932	0.7199	0.1965	0.0958	1.3198	0.2203	0.1131	0.6465	0.1221	0.0300	0.5671	0.1100	0.0749	0.6951	0.2084	0.2793	0.6798	0.3821	0.2265	0.6487	0.2389
0.0776	0.7199	0.2413	0.1169	1.0824	0.1976	0.0510	0.9745	0.2687	0.1057	0.4961	0.1848	0.0825	0.6241	0.1703	0.1198	0.7159	0.2239	0.0432	0.7990	0.0676	0.1125	0.6522	0.2201	0.1317	0.5913	0.2165	0.0962	0.6347	0.2094	0.1106	0.7029	0.1664	0.0399	0.7466	0.0883	0.0897	0.5998	0.1877	0.3105	0.6553	0.3895	0.3954	0.5001	0.2451
0.0017	0.6808	0.2041	0.1013	0.6558	0.2441	0.0381	1.4638	0.1943	0.1187	0.6764	0.2175	0.0346	0.5579	0.1854	0.0520	0.7512	0.1565	0.0735	0.9024	0.1944	0.0811	0.6100	0.1856	0.1123	0.6972	0.2105	0.0891	0.7251	0.2129	0.1014	0.7587	0.1701	0.0402	0.4457	0.0883	0.0763	0.6729	0.1766	0.3514	0.6509	0.4107	0.2543	0.4163	0.3028
0.0914	0.7580	0.2214	0.1293	0.8469	0.2859	0.0979	1.1066	0.2061	0.1043	0.6177	0.1965	0.0385	0.5551	0.1483	0.0715	0.7261	0.1765	0.0752	0.9610	0.1816	0.0735	0.6010	0.1752	0.0866	0.6121	0.1786	0.0849	1.1466	0.2836	0.1268	0.6859	0.2318	0.0198	0.6092	0.1444	0.0844	0.6508	0.1727	0.2644	0.7007	0.3614	0.2513	0.4986	0.2339
0.0906	0.5691	0.2181	0.1707	0.8878	0.2859	0.0445	0.8554	0.0275	0.0958	0.5006	0.1720	0.0108	0.5072	0.1937	0.1557	0.9769	0.2819	0.0646	0.6901	0.1893	0.0735	0.6112	0.1764	0.0967	0.5283	0.1810	0.0827	0.4608	0.2627	0.1061	0.5712	0.1630	0.0480	0.6249	0.0546	0.0737	0.5261	0.0734	0.3905	0.4635	0.4156	0.2941	0.4990	0.2307
0.1008	0.5569	0.2164	0.1707	0.8878	0.0140	0.0446	0.9629	0.1601	0.0954	0.5334	0.1764	0.0152	0.4241	0.1929	0.1494	0.9405	0.2779	0.0645	0.6828	0.1941	0.0159	0.2560	0.0668	0.0962	0.5364	0.1825	0.0868	0.4638	0.2427	0.1072	0.5580	0.1745	0.0480	0.6249	0.0075	0.0747	0.5602	0.2050	0.3912	0.5107	0.4250	0.2957	0.4753	0.2316
0.0990	0.2706	0.0195	0.0090	0.0313	0.1042	0.0000	0.1164	0.2061	0.0079	0.1874	0.0455	0.0684	0.4623	0.1663	0.1114	0.7300	0.2213	0.0230	0.4521	0.1768	0.0815	0.8819	0.2141	0.0214	0.2123	0.0612	0.0038	0.2918	0.1625	0.0065	0.2203	0.1654	0.0098	0.2050	0.0958	0.0182	0.2922	0.1869	0.0170	0.3628	0.0718	0.0850	0.2252	0.4323
0.0979	0.6727	0.0354	0.0462	0.3456	0.1630	0.0410	0.7007	0.1601	0.0601	0.5276	0.1477	0.0701	0.4955	0.1415	0.1014	0.6634	0.1946	0.0188	0.3669	0.2041	0.0774	0.7275	0.2032	0.1303	0.5928	0.2136	0.0685	0.5294	0.1521	0.0577	0.5904	0.1901	0.0248	0.5099	0.1087	0.0696	0.6866	0.1942	0.1564	0.6146	0.2757	0.3882	0.4839	0.1106
0.1146	0.6544	0.1408	0.0806	0.5314	0.1458	0.0476	1.0924	0.1887	0.1116	0.6325	0.2119	0.0743	0.5469	0.1406	0.1004	0.7977	0.2107	0.0188	0.3748	0.1831	0.0943	0.6164	0.1926	0.1316	0.7808	0.2401	0.0986	0.7506	0.1909	0.0627	0.7154	0.2133	0.0584	0.7055	0.1278	0.0979	0.8457	0.2021	0.2672	0.9110	0.3706	0.3319	0.5866	0.2206
0.1065	0.7682	0.1751	0.0511	0.6006	0.2345	0.0541	0.6121	0.1327	0.0725	0.6365	0.1733	0.0684	0.4307	0.0448	0.0062	0.2125	0.0461	0.0263	0.4630	0.1862	0.0944	0.6135	0.1902	0.1104	0.6173	0.2014	0.0722	0.8822	0.1805	0.0699	0.6948	0.2526	0.0324	0.7118	0.1324	0.0837	0.6179	0.1773	0.1598	1.0924	0.2971	0.3244	0.5836	0.3337
0.0970	0.6638	0.2486	0.1220	0.8240	0.1044	0.0575	0.7166	0.1274	0.1036	0.5567	0.2002	0.0710	0.6866	0.1362	0.1123	0.7530	0.2215	0.0408	0.4435	0.1831	0.0555	0.6322	0.1569	0.1411	0.7246	0.2437	0.0919	0.6025	0.1626	0.1265	0.6418	0.1139	0.0399	0.6247	0.2794	0.0833	0.6645	0.1647	0.3084	0.6422	0.3794	0.4328	0.5376	0.3277
0.0979	0.6393	0.2243	0.0559	0.2965	0.1204	0.0313	0.5565	0.1273	0.0713	0.6587	0.1776	0.0713	0.5021	0.1320	0.0921	0.6284	0.1894	0.0339	0.5607	0.1752	0.0541	0.5897	0.1543	0.1182	0.5791	0.2040	0.0680	1.0848	0.1661	0.0506	0.5815	0.1211	0.0350	0.4752	0.1419	0.0766	0.6618	0.1712	0.1650	0.6112	0.2765	0.3114	0.6200	0.3507
0.0979	0.8401	0.2513	0.0559	0.3934	0.1123	0.0313	0.5199	0.1274	0.0664	0.6667	0.1732	0.1051	1.0038	0.1369	0.0945	0.8133	0.2070	0.0382	0.4210	0.1736	0.0828	0.8502	0.2092	0.1159	0.5158	0.1929	0.0679	0.9334	0.2281	0.0540	0.6313	0.1406	0.0350	0.5060	0.1324	0.0798	0.6378	0.1365	0.1764	0.5098	0.2576	0.3153	0.6654	0.1550
0.0017	0.6329	0.2524	0.0559	0.3434	0.1126	0.0377	0.4891	0.1273	0.0866	0.6696	0.1931	0.1078	0.8337	0.1868	0.0945	0.6120	0.1877	0.0382	0.4042	0.1831	0.0672	0.7932	0.1803	0.1463	0.5542	0.2239	0.0849	0.9791	0.2922	0.0826	0.5869	0.1114	0.1874	0.6624	0.1324	0.1106	0.6813	0.1413	0.1650	0.6112	0.2765	0.3898	0.6493	0.1751
0.0888	0.7929	0.1824	0.0559	0.3434	0.1044	0.0314	0.5154	0.1273	0.0695	0.6224	0.1715	0.0996	0.6094	0.1308	0.0992	0.7103	0.1982	0.0382	0.4118	0.0201	0.0713	0.7953	0.1813	0.1169	0.6697	0.2151	0.0721	1.1660	0.2627	0.0541	0.5962	0.1139	0.0350	0.5716	0.1398	0.0808	0.6929	0.1774	0.1764	0.4837	0.2696	0.3167	0.6544	0.2401
0.1024	0.6259	0.2049	0.0559	0.2965	0.1044	0.0313	0.5199	0.0137	0.0713	0.6300	0.1744	0.0995	0.6770	0.1315	0.8609	0.7236	0.7256	0.0314	0.4235	0.1989	0.1094	0.8662	0.2252	0.1173	0.5966	0.2056	0.0680	1.0848	0.2627	0.0506	0.5685	0.1139	0.0350	0.5060	0.1229	0.0766	0.6784	0.1477	0.1650	0.6112	0.2787	0.3146	0.6012	0.1606
0.1039	0.6259	0.1628	0.0559	0.2965	0.0140	0.0313	0.5199	0.2066	0.0713	0.6313	0.1743	0.1414	0.7199	0.1394	0.0921	0.6120	0.1860	0.0518	0.6275	0.2038	0.0753	0.7615	0.1874	0.1173	0.5791	0.2032	0.0680	1.1466	0.2665	0.0506	0.5747	0.1639	0.0350	0.5060	0.1229	0.0766	0.6552	0.1371	0.1650	0.6112	0.2765	0.3129	0.6206	0.1541
0.1029	0.0420	0.2305	0.0090	0.0313	0.1455	0.0000	0.0559	0.1601	0.0000	0.0643	0.0147	0.1449	0.6927	0.0487	0.0020	0.1854	0.0399	0.0536	0.7061	0.0890	0.0672	0.7678	0.1779	0.0035	0.0314	0.0107	0.0000	0.0437	0.1489	0.0018	0.0420	0.1979	0.0000	0.0000	0.1490	0.0085	0.0778	0.1339	0.0073	0.0708	0.0241	0.0182	0.0428	0.1550
0.0953	0.6763	0.1164	0.0706	0.4753	0.1458	0.0314	1.3220	0.1328	0.1082	0.5790	0.2018	0.1007	0.5710	0.1640	0.1043	0.6409	0.2000	0.0874	0.8174	0.0075	0.0672	0.8062	0.1814	0.1356	0.7774	0.2426	0.0955	0.7861	0.0865	0.0527	0.8380	0.2174	0.0350	0.4788	0.1489	0.0889	0.7678	0.1683	0.2848	0.7680	0.3829	0.3330	0.5390	0.3871
0.0929	0.7859	0.1575	0.0511	0.6006	0.2852	0.0541	0.6121	0.1327	0.0725	0.6112	0.1699	0.1009	0.5184	0.1362	0.1123	0.7726	0.2212	0.0892	0.9036	0.0691	0.0884	0.8398	0.2153	0.1099	0.6023	0.1990	0.0722	0.8381	0.0049	0.0699	0.7253	0.2064	0.0324	0.6328	0.0038	0.0841	0.6113	0.1715	0.1597	1.0425	0.2948	0.3229	0.5651	0.2428
0.1025	0.6179	0.1314	0.1424	1.2388	0.2852	0.0061	0.7311	0.1545	0.0587	0.5510	0.1485	0.0702	0.4978	0.1710	0.1709	0.9784	0.2960	0.0759	0.7495	0.0075	0.0146	0.1738	0.0524	0.1158	0.6154	0.2062	0.0955	0.5163	0.1618	0.0737	0.6651	0.2028	0.0453	0.4202	0.1396	0.0891	0.6442	0.1691	0.2913	0.5831	0.3744	0.3806	0.5573	0.0058
0.1007	0.6113	0.1241	0.1424	1.2388	0.1044	0.0062	0.7238	0.1943	0.0575	0.5359	0.1454	0.0767	0.4693	0.1749	0.1657	0.9808	0.2879	0.0755	0.7443	0.0012	0.0668	0.6370	0.1759	0.1140	0.6034	0.2030	0.0997	0.5199	0.1324	0.0758	0.6353	0.0932	0.0453	0.4788	0.0307	0.0888	0.6827	0.1555	0.2917	0.6086	0.3744	0.3813	0.5289	0.3021
0.0993	0.7141	0.1163	0.0415	0.3704	0.1635	0.0410	0.6566	0.1490	0.0637	0.5364	0.1485	0.0032	0.3272	0.1440	0.0424	0.7652	0.1624	0.0114	0.3390	0.1815	0.0779	0.9269	0.2057	0.1313	0.6168	0.2192	0.0615	0.5829	0.1423	0.0572	0.6495	0.0924	0.0148	0.7231	0.0038	0.0691	0.7192	0.1618	0.1760	0.5726	0.2675	0.3833	0.5392	0.3044
0.1211	0.9115	0.1163	0.0707	0.5959	0.1463	0.0476	0.9541	0.1886	0.1112	0.5842	0.1974	0.0070	0.3566	0.1571	0.0382	0.7772	0.1624	0.0114	0.3467	0.1897	0.0780	0.8766	0.2022	0.1255	0.7423	0.2308	0.0912	0.8674	0.0949	0.0607	0.7216	0.1792	0.0638	0.9933	0.0038	0.0943	0.9014	0.1916	0.2861	0.8378	0.3779	0.3162	0.6091	0.3562
0.1079	0.7215	0.2550	0.0415	0.6721	0.2345	0.0541	0.5356	0.1382	0.0783	0.6134	0.1715	0.0022	0.1782	0.1671	0.1150	0.7772	0.2288	0.0073	0.1835	0.1736	0.0053	0.2111	0.0465	0.1087	0.5535	0.1912	0.0658	0.9136	0.1635	0.0699	0.7577	0.1848	0.0248	0.7263	0.1176	0.0854	0.6489	0.1810	0.1561	1.0239	0.2795	0.3080	0.5673	0.3630
0.0984	0.7535	0.1865	0.1115	0.9209	0.1046	0.0575	0.7166	0.1545	0.0974	0.5565	0.1841	0.0065	0.4600	0.1395	0.0578	0.6278	0.1555	0.0373	0.4558	0.2187	0.0703	0.7449	0.1815	0.1405	0.7157	0.2423	0.0846	0.6284	0.1795	0.1253	0.6711	0.2474	0.0298	0.9375	0.0674	0.0827	0.7280	0.2080	0.2942	0.6945	0.3795	0.4134	0.5850	0.4102
0.0993	0.6556	0.1676	0.0463	0.3434	0.1206	0.0313	0.5949	0.1381	0.0746	0.8067	0.1914	0.0043	0.3161	0.1703	0.0435	0.8818	0.1692	0.0356	0.6869	0.2272	0.0513	0.4933	0.1337	0.1211	0.5793	0.2078	0.0637	1.1594	0.2198	0.0497	0.6581	0.1866	0.0274	0.6197	0.0880	0.0663	0.6978	0.2120	0.1666	0.7657	0.2727	0.2916	0.6154	0.3583
0.0993	0.8891	0.2202	0.0463	0.4470	0.1126	0.0313	0.7238	0.1545	0.0722	0.6335	0.1691	0.0429	0.8050	0.1585	0.0403	0.8914	0.1659	0.0347	0.4329	0.0945	0.0569	0.5199	0.1422	0.1153	0.5531	0.1975	0.0637	1.0486	0.1753	0.0526	0.6783	0.1949	0.0274	0.5842	0.1132	0.0691	0.6815	0.2384	0.1781	0.7091	0.2658	0.2968	0.7025	0.3526
0.0034	0.6628	0.2184	0.0463	0.3934	0.1129	0.0377	0.5614	0.1381	0.0901	0.8192	0.2073	0.0460	0.7587	0.2257	0.0457	0.9054	0.1708	0.0305	0.4392	0.1033	0.0920	0.5759	0.1803	0.1508	0.5507	0.2294	0.0805	1.0433	0.1666	0.0811	0.6526	0.1901	0.1723	0.6001	0.1175	0.1000	0.6984	0.2136	0.1666	0.7657	0.2727	0.3698	0.6436	0.3550
0.0912	0.7958	0.0314	0.0463	0.3934	0.1046	0.0314	0.7166	0.1381	0.0732	0.6507	0.1738	0.0414	0.6625	0.1617	0.0436	0.8738	0.1673	0.0347	0.4235	0.0916	0.0569	0.5120	0.1435	0.1181	0.6821	0.2182	0.0678	1.1793	0.1607	0.0532	0.6974	0.2054	0.0274	0.6570	0.1351	0.0706	0.7680	0.2113	0.1781	0.6753	0.2772	0.2987	0.6771	0.4864
0.1031	0.6419	0.1361	0.0463	0.3434	0.1046	0.0313	0.5949	0.0136	0.0747	0.7295	0.1835	0.0414	0.6961	0.1680	0.8260	1.0143	0.7539	0.0255	0.4898	0.0876	0.0513	0.4847	0.1325	0.1202	0.5909	0.2087	0.0637	1.1594	0.2402	0.0497	0.6438	0.2084	0.0274	0.5842	0.0268	0.0663	0.7030	0.2040	0.1666	0.7657	0.2749	0.2945	0.5983	0.3682
0.1053	0.6419	0.1752	0.0463	0.3434	0.0141	0.0313	0.5949	0.1888	0.0746	0.7734	0.1880	0.0744	0.6089	0.1788	0.0435	0.9054	0.1691	0.0500	0.6832	0.1847	0.0513	0.4847	0.1325	0.1202	0.5793	0.2070	0.0637	1.2285	0.1999	0.0497	0.6433	0.2717	0.0274	0.5842	0.1221	0.0663	0.6909	0.2146	0.1666	0.7657	0.2727	0.2931	0.6160	0.3495
0.1043	0.2706	0.2433	0.0000	0.0639	0.1458	0.0000	0.0559	0.1490	0.0079	0.2075	0.0492	0.0792	0.6227	0.1623	0.1121	0.7266	0.2230	0.0518	0.9727	0.2058	0.0737	0.7298	0.1841	0.0187	0.1957	0.0589	0.0038	0.3575	0.2268	0.0083	0.2168	0.1151	0.0098	0.2050	0.0632	0.0184	0.2913	0.0527	0.0245	0.3850	0.0771	0.0750	0.2231	0.3804
0.0506	0.9822	0.2260	0.0608	0.5353	0.1463	0.0314	1.0622	0.1168	0.1098	0.5144	0.1863	0.0419	0.6576	0.0678	0.0072	0.2194	0.0527	0.0819	1.1861	0.2042	0.0525	0.6688	0.1582	0.1267	0.7388	0.2299	0.0882	0.5706	0.1076	0.0507	0.8170	0.1250	0.0350	0.5381	0.0880	0.0860	0.8617	0.1936	0.2819	0.7494	0.3750	0.3202	0.5884	0.3765
0.0370	0.7942	0.2441	0.0415	0.6721	0.2649	0.0541	0.5356	0.1168	0.0783	0.5888	0.1682	0.0436	0.6462	0.1364	0.0578	0.6444	0.1607	0.0836	1.3036	0.2155	0.0461	0.4598	0.1231	0.1082	0.5452	0.1896	0.0658	0.8674	0.0882	0.0699	0.7734	0.1409	0.0248	0.6462	0.1132	0.0873	0.6648	0.1920	0.1560	1.0830	0.2818	0.3065	0.5492	0.0527
0.0617	0.8167	0.2470	0.1263	1.1618	0.2649	0.0061	0.6004	0.2809	0.0636	0.5600	0.1538	0.0070	0.4511	0.1776	0.1534	1.0943	0.2862	0.0729	0.7082	0.2273	0.0805	0.8060	0.1983	0.1119	0.5487	0.1960	0.0889	0.5636	0.1216	0.0702	0.6522	0.1301	0.0454	0.6067	0.1175	0.0943	0.7098	0.1947	0.2730	0.5121	0.3343	0.3682	0.5167	0.1231

0.0452	0.8082	0.1825	0.1263	1.1618	0.1139	0.0062	0.5949	0.1601	0.0623	0.5526	0.1517	0.0168	0.5564	0.1777	0.1471	0.9204	0.2703	0.0727	0.7007	0.0717	0.0806	0.8016	0.1970	0.1105	0.5514	0.1952	0.0931	0.5676	0.1194	0.0722	0.6229	0.1152	0.0454	0.6822	0.1625	0.0949	0.7775	0.1799	0.2734	0.5355	0.3399	0.3689	0.5215	0.0506
0.0645	0.7834	0.2015	0.0755	0.2524	0.1458	0.0882	1.7515	0.2809	0.0966	0.6928	0.2011	0.0038	0.1083	0.0854	0.0020	0.2730	0.0502	0.0000	0.0273	0.0125	0.0723	0.7381	0.1926	0.1101	0.7180	0.2144	0.0659	1.0055	0.1019	0.0722	0.6119	0.1168	0.0482	0.3688	0.1351	0.0711	0.7271	0.1607	0.1587	0.6716	0.3272	0.2404	0.4840	0.0091
0.0619	0.6000	0.1580	0.0607	0.5393	0.1976	0.0250	0.8108	0.1545	0.0717	0.5722	0.1598	0.0011	0.2799	0.1419	0.1108	0.6451	0.1981	0.0073	0.3440	0.0062	0.0520	0.5502	0.1400	0.0977	0.5598	0.1828	0.0494	0.6665	0.1047	0.0790	0.7235	0.1857	0.0149	0.3091	0.1351	0.0727	0.6315	0.1702	0.0623	0.7881	0.2179	0.1996	0.4234	0.0046
0.0477	0.7514	0.2323	0.0958	0.7045	0.0887	0.1045	1.2219	0.1657	0.1074	0.6779	0.2134	0.0059	0.4725	0.1054	0.0313	0.4971	0.1156	0.0305	0.3925	0.1798	0.0548	0.5137	0.1334	0.1304	0.6606	0.2236	0.0741	0.7096	0.1722	0.1319	0.5641	0.2023	0.0249	0.5381	0.2729	0.0758	0.6089	0.1924	0.3168	0.6280	0.4040	0.3222	0.6188	0.3860
0.0452	0.5542	0.1164	0.0462	0.2539	0.0887	0.0443	0.6296	0.1601	0.0723	0.7271	0.1791	0.0011	0.0514	0.0908	0.0175	0.4294	0.0956	0.0296	0.5505	0.1912	0.0928	0.6082	0.1861	0.0909	0.5933	0.1822	0.0494	1.1151	0.2335	0.0433	0.6596	0.2144	0.0174	0.1282	0.2016	0.0550	0.5378	0.1832	0.0960	0.6492	0.2241	0.1931	0.4807	0.2382
0.0452	0.8996	0.1608	0.0462	0.2539	0.0965	0.0443	0.7166	0.1885	0.0703	0.6638	0.1702	0.0446	0.7371	0.1144	0.0144	0.4612	0.0956	0.0280	0.3712	0.1798	0.0562	0.5238	0.1413	0.0909	0.5594	0.1766	0.0521	1.0287	0.2064	0.0462	0.6496	0.2023	0.0174	0.1470	0.2113	0.0571	0.5639	0.1639	0.1043	0.6468	0.2292	0.1976	0.5275	0.1569
0.0933	0.5408	0.1314	0.0462	0.2984	0.0968	0.0508	0.6355	0.1601	0.0883	0.7233	0.1935	0.0459	0.7262	0.1422	0.0196	0.4168	0.0940	0.0321	0.3681	0.2253	0.0520	0.5227	0.1368	0.1184	0.6127	0.2084	0.0680	1.0327	0.2161	0.0719	0.6653	0.1985	0.1579	0.2640	0.2566	0.0890	0.5740	0.1581	0.0960	0.6492	0.2221	0.2682	0.5079	0.3168
0.0572	0.8155	0.1164	0.0462	0.2984	0.0887	0.0444	0.9178	0.1601	0.0736	0.7017	0.1782	0.0407	0.5585	0.1049	0.0175	0.5650	0.1063	0.0280	0.3626	0.2338	0.0520	0.5409	0.1389	0.0921	0.5992	0.1847	0.0561	1.4785	0.2225	0.0463	0.6795	0.0034	0.0174	0.2492	0.2461	0.0583	0.5949	0.1810	0.1085	0.5853	0.2431	0.1974	0.5349	0.3097
0.0431	0.5424	0.1164	0.0462	0.2539	0.0887	0.0443	0.6718	0.1435	0.0723	0.6956	0.1759	0.0408	0.5651	0.0926	0.7773	0.8005	0.6651	0.0263	0.4281	0.0636	0.0757	0.7231	0.1950	0.0900	0.6110	0.1838	0.0494	1.1151	0.1319	0.0433	0.6527	0.1454	0.0174	0.1470	0.0268	0.0551	0.5421	0.1837	0.0960	0.6492	0.2260	0.1917	0.4807	0.1528
0.0796	0.5424	0.1164	0.0462	0.2539	0.1044	0.0443	0.6718	0.2363	0.0723	0.7170	0.1781	0.0743	0.5511	0.0899	0.0175	0.4168	0.0925	0.0414	0.6109	0.0677	0.0504	0.5994	0.1576	0.0900	0.5992	0.1822	0.0494	1.1796	0.1524	0.0433	0.6448	0.1362	0.0174	0.1470	0.0268	0.0551	0.5324	0.0132	0.0960	0.6492	0.2240	0.1931	0.4811	0.0808
0.0796	0.6586	0.2477	0.0368	0.3961	0.0972	0.0410	0.5754	0.1601	0.0601	0.5433	0.1498	0.0791	0.5346	0.1251	0.1021	0.6602	0.1945	0.0431	0.7585	0.1704	0.0462	0.5006	0.1346	0.1300	0.5998	0.2155	0.0685	0.6207	0.1618	0.0576	0.6280	0.2193	0.0248	0.5099	0.1316	0.0673	0.6582	0.1789	0.1608	0.6461	0.2802	0.3745	0.4984	0.0497
0.0433	0.7212	0.1849	0.0656	0.2119	0.1458	0.0710	1.2071	0.1943	0.0948	0.6990	0.2000	0.0413	0.5181	0.1402	0.0349	0.7553	0.1493	0.0734	0.8909	0.1990	0.0777	0.7516	0.1951	0.1088	0.6729	0.2074	0.0616	0.9178	0.1185	0.0596	0.7292	0.1354	0.0250	0.2184	0.0841	0.0654	0.7321	0.1822	0.1775	0.5130	0.3098	0.2424	0.4744	0.0456
0.0487	0.5823	0.1628	0.0607	0.5393	0.1988	0.0250	0.8108	0.2061	0.0717	0.5569	0.1577	0.0430	0.5084	0.1098	0.0313	0.4971	0.1171	0.0751	0.9488	0.1926	0.0777	0.7356	0.1939	0.0976	0.5442	0.1804	0.0494	0.7014	0.0558	0.0790	0.6908	0.1498	0.0149	0.3091	0.1097	0.0736	0.6596	0.1902	0.0563	0.8512	0.2180	0.1983	0.4086	0.3975
0.0677	0.7943	0.2149	0.1104	0.6200	0.1988	0.0475	0.9715	0.2303	0.0724	0.5975	0.1657	0.0059	0.3977	0.1649	0.1356	0.9368	0.2586	0.0645	0.5933	0.2120	0.0736	0.6416	0.1822	0.1221	0.6350	0.2163	0.0651	0.6433	0.1425	0.0779	0.5692	0.1513	0.0352	0.4377	0.1266	0.0768	0.5396	0.1894	0.2421	0.4402	0.3317	0.2880	0.5212	0.2413
0.0827	0.7943	0.2131	0.1104	0.6200	0.1380	0.0476	1.0998	0.2241	0.0741	0.5953	0.1667	0.0163	0.5270	0.1657	0.1294	0.9515	0.2548	0.0644	0.6102	0.2237	0.0786	0.6364	0.1857	0.1193	0.6228	0.2121	0.0692	0.6482	0.2322	0.0784	0.5592	0.1871	0.0352	0.4665	0.1220	0.0795	0.5675	0.2363	0.2425	0.4139	0.3259	0.2894	0.4827	0.1751
0.0791	0.7539	0.1578	0.0857	0.3412	0.2067	0.0883	0.9374	0.2363	0.0912	0.6775	0.1982	0.0048	0.3076	0.1426	0.1098	0.6998	0.2018	0.0073	0.3432	0.0062	0.1136	0.7103	0.2288	0.0810	0.5260	0.1629	0.0619	0.8451	0.1984	0.0856	0.7598	0.1783	0.0403	0.4377	0.1221	0.0912	0.7168	0.1990	0.1681	1.2581	0.3467	0.1404	0.4950	0.3240
0.0665	0.6967	0.1571	0.1065	0.6929	0.0963	0.0611	0.9952	0.2429	0.1199	0.7673	0.2375	0.0092	0.4772	0.1365	0.0335	0.7514	0.1427	0.0305	0.3739	0.1846	0.0812	0.7071	0.1986	0.1108	0.7134	0.2125	0.0785	0.6987	0.2394	0.1171	0.6511	0.2351	0.0665	0.5360	0.0632	0.0823	0.6595	0.1908	0.3726	0.7776	0.4492	0.3010	0.5179	0.3191
0.0677	0.9027	0.1947	0.0658	0.2094	0.0963	0.0848	1.0419	0.2488	0.1019	0.8356	0.2243	0.0027	0.1001	0.1181	0.0155	0.6609	0.1205	0.0296	0.5491	0.1944	0.0736	0.6523	0.1834	0.0927	0.7187	0.1991	0.0830	0.9814	0.2017	0.0637	0.7488	0.0862	0.0562	0.3537	0.0880	0.0899	0.8615	0.1882	0.2169	1.2614	0.3543	0.1395	0.5815	0.1512
0.0677	1.1155	0.2261	0.0658	0.2094	0.1042	0.0848	1.1161	0.2426	0.0975	0.7302	0.2093	0.0458	0.7242	0.1206	0.0124	0.6859	0.1190	0.0280	0.3617	0.1767	0.0736	0.6632	0.1846	0.0905	0.7021	0.1937	0.0901	1.0639	0.2093	0.0681	0.8209	0.0939	0.0562	0.3792	0.1132	0.0934	0.8721	0.1866	0.2171	0.9580	0.3414	0.1422	0.6079	0.1259
0.0926	0.9186	0.2062	0.0658	0.2508	0.1044	0.0915	1.1314	0.2364	0.1158	0.8683	0.2406	0.0471	0.7798	0.1689	0.0176	0.6439	0.1189	0.0322	0.3587	0.2204	0.0166	0.2982	0.0729	0.1166	0.6742	0.2155	0.1003	0.9861	0.2166	0.0932	0.7300	0.1152	0.2004	0.5079	0.1175	0.1255	0.8546	0.1772	0.2169	1.2614	0.3566	0.2199	0.5858	0.1222
0.0191	1.2761	0.2166	0.0658	0.2508	0.0963	0.0850	1.1012	0.2364	0.1040	0.8463	0.2278	0.0385	0.6180	0.1229	0.0155	0.7089	0.1204	0.0280	0.3532	0.2306	0.0830	0.9406	0.2190	0.0937	0.7911	0.2080	0.0879	1.1620	0.2588	0.0642	0.7837	0.0996	0.0562	0.5218	0.1221	0.0922	0.8898	0.1831	0.2123	0.8660	0.3479	0.1388	0.6111	0.4458
0.0485	0.9027	0.1946	0.0658	0.2094	0.0963	0.0848	1.0419	0.2061	0.1020	0.7774	0.2184	0.0385	0.6331	0.1198	0.7787	0.8386	0.6752	0.0263	0.4559	0.1799	0.0803	0.7628	0.2080	0.0918	0.7256	0.1992	0.0830	0.9814	0.2470	0.0648	0.7480	0.0839	0.0562	0.3792	0.0632	0.0899	0.8983	0.1609	0.2169	1.2614	0.3543	0.1408	0.5903	0.2985
0.0663	0.9027	0.1946	0.0658	0.2094	0.1546	0.0848	1.0419	0.0909	0.1019	0.8240	0.2231	0.0779	0.6100	0.1265	0.0155	0.6439	0.1174	0.0414	0.6092	0.1897	0.0943	0.6473	0.1914	0.0918	0.7118	0.1976	0.0830	1.0345	0.2350	0.0637	0.7323	0.0854	0.0562	0.3792	0.0880	0.0899	0.8528	0.1659	0.2169	1.2614	0.3520	0.1408	0.5909	0.2423
0.0663	0.6828	0.2169	0.0707	0.5314	0.0141	0.0476	1.0924	0.2303	0.1116	0.6414	0.2131	0.0810	0.5653	0.1304	0.1011	0.6978	0.1981	0.0431	0.7562	0.1720	0.0944	0.6442	0.1914	0.1323	0.7599	0.2388	0.0986	0.7897	0.1623	0.0626	0.7185	0.1379	0.0584	0.7055	0.1132	0.0971	0.8543	0.0220	0.2724	0.8662	0.3732	0.3180	0.5912	0.3915
0.0720	0.3084	0.0738	0.0090	0.0311	0.1380	0.0156	0.3644	0.1713	0.0111	0.1523	0.0404	0.0374	0.5782	0.1595	0.0307	0.7672	0.1494	0.0734	0.9055	0.2204	0.0079	0.1329	0.0361	0.0119	0.1358	0.0389	0.0038	0.2461	0.1662	0.0169	0.1786	0.1896	0.0326	0.2953	0.1175	0.0111	0.2248	0.0379	0.0145	0.3459	0.0708	0.0195	0.1009	0.3812
0.0618	0.8211	0.1690	0.0857	0.3412	0.2265	0.0883	0.9374	0.1770	0.0907	0.6540	0.1948	0.0413	0.5908	0.1396	0.0335	0.7514	0.1461	0.0752	0.9644	0.2289	0.0378	0.0800	0.0475	0.0810	0.4999	0.1592	0.0619	0.9861	0.1908	0.0856	0.7927	0.1832	0.0403	0.4377	0.1177	0.0920	0.7248	0.0501	0.1680	1.1959	0.3441	0.1392	0.4790	0.0506
0.0739	0.7366	0.1823	0.1005	0.9592	0.2265	0.0411	0.7852	0.2552	0.0996	0.6588	0.1965	0.0076	0.4051	0.1839	0.1370	0.8454	0.2549	0.0646	0.6035	0.1879	0.0159	0.3018	0.0702	0.0951	0.6388	0.1931	0.0660	0.6960	0.1658	0.0854	0.6291	0.1786	0.0535	0.5750	0.1353	0.1032	0.6899	0.0035	0.3235	0.5330	0.3826	0.2441	0.5962	0.0506
0.0750	0.6630	0.1740	0.1005	0.9592	0.2251	0.0412	0.8284	0.0961	0.1009	0.6239	0.1932	0.0174	0.5762	0.1847	0.1309	0.9528	0.2588	0.0645	0.6207	0.1720	0.0000	0.0229	0.0045	0.0942	0.6266	0.1906	0.0701	0.7380	0.1661	0.0875	0.6079	0.1812	0.0535	0.6097	0.1175	0.1032	0.7099	0.0015	0.3240	0.5573	0.3855	0.2441	0.5533	0.3893
0.0581	0.7618	0.1853	0.1185	0.7657	0.1203	0.1189	0.8109	0.1012	0.1084	0.7306	0.2201	0.0043	0.3925	0.1440	0.1207	0.7230	0.2217	0.0364	0.4521	0.1893	0.0000	0.0137	0.0027	0.0933	0.6432	0.1904	0.0871	0.9012	0.1659	0.1389	0.6871	0.1173	0.0198	0.6092	0.1263	0.0815	0.6689	0.1753	0.2855	0.9006	0.3888	0.2852	0.6069	0.2333
0.0618	0.5928	0.1564	0.0559	0.3934	0.1203	0.0444	0.2748	0.1011	0.0700	0.6783	0.1710	0.0021	0.2901	0.1390	0.1015	0.6609	0.2018	0.0347	0.6188	0.1957	0.0842	0.6603	0.1939	0.0676	0.5605	0.1559	0.0682	1.0075	0.2093	0.0753	0.7490	0.1244	0.0224	0.3435	0.0545	0.0769	0.5558	0.1967	0.0865	0.9901	0.2036	0.0929	0.4421	0.1616
0.0618	0.8125	0.1891	0.0559	0.3934	0.1285	0.0444	0.3008	0.1063	0.0653	0.6028	0.1571	0.0435	0.7619	0.1393	0.1038	0.7131	0.2072	0.0339	0.4679	0.1846	0.0689	0.8477	0.1966	0.0650	0.5442	0.1501	0.0702	1.1664	0.2791	0.0826	0.7706	0.1351	0.0224	0.3435	0.0831	0.0800	0.6119	0.1644	0.0927	0.8717	0.1925	0.1001	0.5140	0.3249
0.1027	0.6037	0.1691	0.0559	0.4470	0.1285	0.0508	0.2768	0.0960	0.0860	0.7139	0.1897	0.0460	0.7700	0.1953	0.1038	0.6439	0.2000	0.0381	0.4647	0.2089	0.0634	0.5460	0.1488	0.0990	0.5828	0.1867	0.0873	1.0974	0.2470	0.1075	0.7890	0.1486	0.1638	0.4170	0.0873	0.1095	0.5772	0.1771	0.0865	0.9901	0.2055	0.1750	0.4472	0.3177
0.0507	0.6422	0.1564	0.0559	0.4470	0.1203	0.0445	0.3253	0.0960	0.0711	0.6290	0.1660	0.0408	0.5787	0.1393	0.1075	0.6998	0.2071	0.0339	0.4580	0.2188	0.0759	0.7462	0.1916	0.0655	0.6475	0.1652	0.0765	1.9969	0.2585	0.0826	0.9010	0.1124	0.0224	0.4796	0.0915	0.0840	0.6828	0.1804	0.0906	0.8509	0.2142	0.0954	0.4862	0.0061
0.0086	0.5803	0.1548	0.0559	0.3934	0.1203	0.0444	0.2748	0.1546	0.0700	0.6488	0.1678	0.0408	0.6249	0.1369	0.8679	0.8240	0.7508	0.0321	0.4708	0.2006	0.0760	0.7302	0.1904	0.0667	0.5661	0.1559	0.0682	1.0075	0.2624	0.0763	0.7252	0.1149	0.0224	0.3435	0.1087	0.0769	0.5802	0.0596	0.0865	0.9421	0.2016	0.0929	0.4421	0.3749
0.0761	0.5803	0.1548	0.0559	0.3934	0.1376	0.0444	0.2748	0.2302	0.0700	0.6879	0.1722	0.0738	0.6060	0.1399	0.1015	0.6439	0.1983	0.0474	0.6032	0.2071	0.0468	0.2009	0.0821	0.0667	0.5661	0.1559	0.0682	1.0626	0.0922	0.0753	0.7408	0.1807	0.0224	0.3435	0.0152	0.0769	0.5502	0.0605	0.0865	0.9901	0.2017	0.0929	0.4425	0.2300
0.0761	0.7522	0.2258	0.0415	0.6006	0.1380	0.0541	0.5729	0.0000	0.0725	0.6456	0.1745	0.0792	0.5999	0.0488	0.0082	0.1944	0.0403	0.0491	0.8094	0.0150	0.0213	0.3579	0.0843	0.1113	0.5985	0.1986	0.0722	1.0327	0.1657	0.0708	0.7136	0.1695	0.0324	0.7118	0.1046	0.0837	0.6519	0.0252	0.1628	1.1116	0.3018	0.3108	0.5703	0.1560
0.0771	0.6869	0.1581	0.0755	0.3934	0.0000	0.0848	0.9745	0.2123	0.0930	0.6209	0.1926	0.0413	0.5898	0.1625	0.1079	0.7191	0.2144	0.0815	1.0105	0.2186	0.0079	0.1222	0.0342	0.0779	0.5380	0.1607	0.0590	0.9329	0.1144	0.0785	0.8111	0.2402	0.0174	0.1879	0.0669	0.0855	0.7024	0.0226	0.1705	0.7690	0.3059	0.1405	0.4877	0.3119
0.1054	0.0850	0.0248	0.0000	0.0000	0.3061	0.0000	0.0000	0.2184	0.0000	0.0275	0.0069	0.0430	0.5792	0.1454	0.1207	0.7418	0.2251	0.0833	1.0790	0.2324	0.0079	0.1329	0.0361	0.0000	0.0326	0.0072	0.0000	0.0429	0.0766	0.0000	0.0468	0.1678	0.0000	0.0324	0.0627	0.0028	0.0511	0.1767	0.0037	0.0403	0.0054	0.0011	0.0212	0.3071
0.0916	0.7942	0.1954	0.1424	1.6636	0.3061	0.0607	1.0331	0.2426	0.0529	0.6243	0.1543	0.0070	0.4328	0.1710	0.1809	0.7761	0.2888	0.0690	0.6989	0.0554	0.0894	0.6336	0.1939	0.0849	0.5690	0.1742	0.0680	0.6785	0.0223	0.1053	0.7669	0.1802	0.0249	0.3844	0.0792	0.0940	0.6402	0.2001	0.2580	0.5314	0.3663	0.2257	0.5810	0.3762
0.0759	0.7778	0.1937	0.1424	1.6636	0.0140	0.0609	1.0924	0.2302	0.0533	0.6576	0.1586	0.0168	0.4934	0.1742	0.1757	0.8400	0.2908	0.0689	0.6394	0.2090	0.0747	0.9354	0.2048	0.0811	0.5880	0.1734	0.0722	0.7570	0.1725	0.1053	0.7417	0.1684	0.0249	0.4682	0.1494	0.0943	0.6617	0.1739	0.2584	0.5021	0.3605	0.2279	0.5937	0.2310
0.0771	1.0274	0.2147	0.1011	0.5913	0.0070	0.0916	0.9178	0.2425	0.0925	0.7711	0.2107	0.0070	0.4655	0.1239	0.0399	0.4302	0.1156	0.0263	0.3683	0.1767	0.0692	0.5743	0.1610	0.0993	0.7903	0.2120	0.0958	1.7446	0.1677	0.1280	0.7014	0.2043	0.0300	0.5671	0.1547	0.0811	0.7625	0.1836	0.3009	0.6532	0.3783	0.2686	0.6714	0.1569
0.0771	0.9617	0.2347	0.0960	0.6254	0.0070	0.0916	0.8065	0.2427	0.0944	0.7329	0.2073	0.0453	0.8744	0.1400	0.0384	0.5476	0.1282	0.0024	0.0241	0.1798	0.0802	0.7340	0.1915	0.0968	0.7382	0.2039	0.0986	1.4571	0.2102	0.1328	0.7434	0.2184	0.0300	0.6018	0.1587	0.0821	0.7469	0.1882	0.3093	0.7354	0.3932	0.2779	0.6712	0.3130
0.1020	1.0345	0.2288	0.1011	0.5913	0.0000	0.0984	0.8697	0.2425	0.1084	0.7723	0.2257	0.0495	0.8894	0.1737	0.0420	0.4177	0.1138	0.0065	0.0324	0.0689	0.0803	0.7183	0.1903	0.1264	0.7843	0.2362	0.1133	1.6272	0.1827	0.1544	0.7085	0.2521	0.1868	0.7037	0.1635	0.1143	0.7595	0.0870	0.3009	0.6532	0.3806	0.3422	0.6861	0.3059
0.0580	1.1334	0.2217	0.1011	0.6612	0.0000	0.0918	0.9074	0.2425	0.0920	0.8032	0.2143	0.0406	0.7854	0.1438	0.0378	0.6111	0.1338	0.0024	0.0187	0.1735	0.0546	0.3457	0.1120	0.0955	0.7942	0.2138	0.1029	1.6988	0.1737	0.1353	0.7343	0.1182	0.0300	0.6764	0.0832	0.0843	0.8140	0.0395	0.3093	0.7354	0.4065	0.2732	0.7106	0.4161
0.0730	1.0051	0.2130	0.1011	0.5913	0.1046	0.0916	0.9178	0.1944	0.0925	0.7177	0.2050	0.0406	0.8683	0.1294	0.8019	0.8386	0.8111	0.0180	0.2790	0.1863	0.0378	0.0849	0.0485	0.0984	0.8056	0.2128	0.0958	1.7446	0.1633	0.1291	0.6864	0.1197	0.0300	0.6018	0.1449	0.0811	0.7747	0.0370	0.3009	0.6218	0.3807	0.2656	0.6816	0.3845
0.0766	1.0051	0.2130	0.1011	0.5913	0.0963	0.0916	0.9178	0.2064	0.0925	0.7605	0.2096	0.0774	0.8293	0.1331	0.0399	0.4177	0.1121	0.0450	0.4143	0.1863	0.0378	0.0701	0.0456	0.0984	0.7903	0.2112	0.0958	1.9031	0.2578	0.1280	0.6859	0.1422	0.0300	0.6018	0.1097	0.0811	0.7550	0.2072	0.3009	0.6532	0.3808	0.2655	0.6823	0.4310

0.0766	0.6998	0.2073	0.1115	0.8240	0.1203	0.0575	0.7643	0.2552	0.1036	0.5567	0.2013	0.0823	0.7456	0.1280
0.0436	0.6618	0.1622	0.0960	0.6254	0.2664	0.0445	0.9686	0.2120	0.1203	0.6589	0.2246	0.0411	0.7896	0.1387
0.0174	0.8182	0.1921	0.1185	0.7657	0.2664	0.1189	0.8109	0.2121	0.1091	0.7171	0.2200	0.0428	0.7963	0.0095
0.0035	0.5646	0.1637	0.1648	0.9492	0.0212	0.0509	0.9924	0.0322	0.0910	0.5082	0.1731	0.0114	0.6279	0.1649
0.0999	0.5770	0.1653	0.1648	0.9492	0.0211	0.0510	0.9804	0.0137	0.0906	0.5037	0.1720	0.0191	0.5989	0.1681
0.0628	0.4243	0.1131	0.0000	0.0639	0.0140	0.0000	0.1359	0.0417	0.0045	0.2408	0.0518	0.0434	0.7179	0.0438
0.0671	0.0232	0.0181	0.0000	0.0313	0.0140	0.0062	0.0366	0.0045	0.0150	0.0347	0.0191	0.0448	0.6895	0.0533
0.0812	0.3138	0.0657	0.0000	0.0313	0.1206	0.0000	0.1785	0.0045	0.0034	0.2952	0.0602	0.0419	0.5963	0.0472
0.0812	0.0057	0.0013	0.0000	0.0000	0.0963	0.0000	0.0180	0.1274	0.0000	0.0318	0.0069	0.0419	0.6111	0.0051
0.0584	0.0057	0.0013	0.0000	0.0000	0.1203	0.0000	0.0180	0.2061	0.0000	0.0118	0.0026	0.0755	0.5963	0.0029
0.0461	0.6669	0.2148	0.0463	0.3434	0.2664	0.0313	0.5199	0.0961	0.0713	0.6872	0.1811	0.0779	0.5636	0.1268
0.0436	0.8501	0.1863	0.0559	0.2524	0.2664	0.0676	0.8884	0.1827	0.1017	0.8524	0.2254	0.0402	0.5392	0.1655
0.0436	0.6600	0.1691	0.0559	0.3934	0.0140	0.0444	0.2748	0.1885	0.0700	0.6423	0.1678	0.0441	0.5661	0.1299
0.1166	0.9028	0.2126	0.1370	1.0282	0.0070	0.0410	0.9090	0.0275	0.0541	0.7427	0.1679	0.0048	0.3796	0.1783
0.0721	0.8839	0.2109	0.1370	1.0282	0.0070	0.0411	0.9598	0.0417	0.0555	0.7732	0.1722	0.0152	0.5194	0.1767
0.0774	0.4542	0.1296	0.0000	0.0980	0.1126	0.0062	0.0951	0.0275	0.0196	0.2650	0.0687	0.0072	0.3159	0.0965
0.1063	0.6000	0.1389	0.0000	0.0980	0.1042	0.0000	0.1785	0.0275	0.0056	0.3299	0.0687	0.0506	0.8776	0.0524
0.1073	0.4243	0.1131	0.0000	0.0639	0.1285	0.0000	0.1149	0.1381	0.0045	0.2090	0.0463	0.0506	0.8644	0.0423
0.0201	0.4243	0.1131	0.0000	0.0639	0.2673	0.0000	0.1149	0.2183	0.0045	0.2249	0.0490	0.0786	0.8673	0.0443
0.0174	0.8956	0.2599	0.0463	0.4470	0.2673	0.0313	0.5949	0.1012	0.0664	0.6667	0.1733	0.0781	0.6739	0.1336
0.0174	0.9371	0.2040	0.0559	0.2524	0.0070	0.0676	1.0137	0.2003	0.0973	0.7446	0.2104	0.0489	0.8066	0.1625
0.1085	0.8855	0.1991	0.0559	0.3934	0.0070	0.0444	0.3008	0.2063	0.0653	0.5784	0.1551	0.0529	0.8135	0.1439
0.0742	1.2887	0.2633	0.1370	1.0282	0.1129	0.0410	1.1148	0.0417	0.0495	0.6619	0.1550	0.0401	0.8933	0.1866
0.0805	1.2727	0.2633	0.1370	1.0282	0.1044	0.0411	1.1809	0.0091	0.0509	0.7085	0.1613	0.0463	0.8926	0.1859
0.0929	0.3349	0.0826	0.0000	0.0639	0.1285	0.0062	0.1574	0.0091	0.0184	0.3449	0.0811	0.0546	0.7481	0.0958
0.0929	0.0173	0.0168	0.0000	0.0313	0.2770	0.0062	0.0181	0.1221	0.0150	0.0593	0.0244	0.0547	0.7372	0.0568
0.0035	0.0173	0.0168	0.0000	0.0313	0.2770	0.0062	0.0181	0.2180	0.0150	0.0306	0.0182	0.0745	0.8187	0.0585
0.0035	0.6602	0.2215	0.0463	0.3934	0.1046	0.0377	0.4554	0.1011	0.0866	0.6984	0.1966	0.0727	0.6869	0.1777
0.0990	0.8650	0.1978	0.0559	0.2965	0.0963	0.0743	0.9598	0.1885	0.1156	0.8735	0.2407	0.0529	0.7333	0.2160
0.0654	0.6719	0.1821	0.0559	0.4470	0.1203	0.0508	0.2768	0.1943	0.0859	0.6764	0.1864	0.0569	0.7027	0.1811
0.0634	0.8806	0.2213	0.1370	1.0282	0.2664	0.0474	0.9196	0.0369	0.0679	0.7238	0.1788	0.0431	0.7899	0.2247
0.0791	0.8623	0.2195	0.1370	1.0282	0.2664	0.0475	0.9715	0.0369	0.0693	0.7744	0.1854	0.0483	0.8736	0.2221
0.0791	0.3138	0.0657	0.0000	0.0313	0.1046	0.0000	0.1564	0.1381	0.0034	0.2777	0.0574	0.0000	0.1882	0.0456
0.0999	0.3138	0.0657	0.0000	0.0313	0.0963	0.0000	0.1564	0.2180	0.0034	0.3126	0.0630	0.0821	0.5318	0.0443
0.0628	0.8097	0.2323	0.0463	0.3934	0.1203	0.0314	0.5896	0.1063	0.0695	0.6136	0.1705	0.0874	0.5276	0.1270
0.0671	1.0385	0.2014	0.0559	0.2965	0.2664	0.0678	1.0013	0.1828	0.1038	0.8395	0.2267	0.0011	0.1929	0.1625
0.0812	0.6570	0.1627	0.0559	0.4470	0.2664	0.0445	0.3253	0.2003	0.0711	0.6213	0.1662	0.0021	0.1978	0.1508
0.0812	0.9815	0.2180	0.1370	1.1618	0.1372	0.0411	0.8986	0.1221	0.0540	0.8354	0.1781	0.0408	0.6146	0.1879
0.0999	0.9815	0.2180	0.1370	1.1618	0.1376	0.0411	1.0852	0.2061	0.0554	0.8461	0.1803	0.0402	0.7255	0.1863
0.0628	0.0000	0.2131	0.0000	0.0000	0.2753	0.0000	0.0000	0.0960	0.0000	0.0358	0.0078	0.0822	0.5527	0.0050
0.0671	0.6530	0.1847	0.0463	0.3434	0.2753	0.0313	0.4850	0.1885	0.0713	0.6390	0.1756	0.0874	0.5557	0.1247
0.0812	0.8326	0.1675	0.0559	0.2524	0.1380	0.0676	0.8884	0.1943	0.1018	0.7818	0.2185	0.0011	0.2019	0.1648
0.0812	0.6464	0.2109	0.0559	0.3934	0.2265	0.0444	0.2748	0.1221	0.0700	0.6319	0.1668	0.0027	0.2045	0.1355
0.0908	0.8839	0.2092	0.1370	1.0282	0.2265	0.0410	0.9715	0.2061	0.0541	0.7110	0.1647	0.0408	0.6215	0.1799
0.1044	0.8654	0.2131	0.1370	1.0282	0.3061	0.0411	1.0265	0.0960	0.0555	0.7609	0.1711	0.0402	0.7058	0.1791
0.1058	0.6530	0.1847	0.0463	0.3434	0.3061	0.0313	0.4850	0.1885	0.0713	0.6587	0.1778	0.0119	0.1612	0.1278

0.1130	0.8311	0.2276	0.0484	0.5566	0.1846	0.1241	0.7017	0.2375	0.1441	0.7085	0.2445	0.0919	0.7036	0.2398
0.0502	0.7057	0.1557	0.0920	0.9807	0.1689	0.1108	0.8859	0.2389	0.1116	0.7272	0.2141	0.0805	0.9378	0.2100
0.0000	0.0071	0.0030	0.0938	1.0674	0.1894	0.1042	0.6285	0.1978	0.0933	0.6494	0.1912	0.0871	0.9492	0.1223
0.1492	1.2694	0.2925	0.0739	0.7214	0.1847	0.1157	0.8550	0.2400	0.1034	0.6207	0.1985	0.0742	0.4872	0.1084
0.1429	1.1828	0.2836	0.0738	0.7003	0.0087	0.1158	0.8228	0.2375	0.1029	0.6298	0.2009	0.0783	0.4380	0.1488
0.0061	0.2048	0.0399	0.0238	0.3477	0.1942	0.0159	0.2818	0.0672	0.0102	0.0905	0.0289	0.0000	0.2441	0.1346
0.0020	0.0072	0.0040	0.0280	0.3447	0.1752	0.0159	0.3018	0.0702	0.0382	0.0480	0.0426	0.0175	0.0501	0.1225
0.0041	0.2326	0.0457	0.0238	0.3394	0.1893	0.0885	0.6307	0.1974	0.0142	0.1752	0.0486	0.0038	0.4175	0.1253
0.7830	0.7201	0.6265	0.0280	0.4162	0.1957	0.0771	0.7629	0.1992	0.0008	0.0216	0.0054	0.0000	0.0282	0.1860
0.0000	0.0072	0.0027	0.0432	0.6435	0.1373	0.0720	0.5952	0.1645	0.0008	0.0161	0.0042	0.0000	0.0140	0.2514
0.0928	0.6764	0.1999	0.0449	0.6444	0.2022	0.0790	0.7971	0.2011	0.1195	0.5537	0.2020	0.0680	1.0848	0.2398
0.0360	0.8045	0.1544	0.0831	0.9686	0.2341	0.0791	0.8060	0.2023	0.0941	0.7344	0.2007	0.0829	1.0503	0.2298
0.0399	0.4430	0.1125	0.0848	1.0990	0.1898	0.0000	0.0183	0.0036	0.0676	0.5775	0.1581	0.0682	1.0626	0.2294
0.1332	1.0124	0.2605	0.0711	0.9656	0.1897	0.0842	0.6603	0.1939	0.0918	0.6874	0.1923	0.0915	1.3115	0.1618
0.1270	1.0289	0.2567	0.0710	0.9762	0.2136	0.0689	0.8340	0.1954	0.0895	0.6646	0.1884	0.0871	1.3282	0.1400
0.0082	0.1954	0.0385	0.0041	0.0188	0.1992	0.0634	0.5553	0.1499	0.0458	0.1039	0.0607	0.0174	0.2352	0.1494
0.0061	0.2826	0.0527	0.0000	0.0053	0.2062	0.0759	0.7841	0.1951	0.0119	0.2165	0.0544	0.0038	0.3727	0.0978
0.7912	0.8575	0.6488	0.0155	0.2758	0.2126	0.0760	0.7672	0.1940	0.0095	0.0905	0.0282	0.0000	0.2066	0.1635
0.0061	0.1954	0.0371	0.0424	0.4483	0.2060	0.0842	0.6713	0.1951	0.0095	0.0785	0.0257	0.0000	0.2251	0.1795
0.0952	0.8758	0.2178	0.0458	0.5983	0.2142	0.0689	0.8477	0.1967	0.1172	0.5028	0.1909	0.0679	0.8862	0.2198
0.0328	0.9276	0.1594	0.0892	0.9366	0.2092	0.0634	0.5553	0.1499	0.0901	0.7174	0.1936	0.0899	1.2094	0.1785
0.0384	0.5625	0.1235	0.0910	1.0182	0.2045	0.0759	0.7841	0.1951	0.0650	0.5609	0.1522	0.0702	1.2355	0.1666
0.1402	0.9876	0.2643	0.0712	0.6895	0.2087	0.0760	0.7672	0.1939	0.0892	0.6672	0.1893	0.0936	1.0276	0.1607
0.1340	1.0595	0.2645	0.0711	0.6691	0.2377	0.0909	0.9063	0.2250	0.0888	0.6325	0.1853	0.0891	1.0380	0.2402
0.0061	0.2229	0.0442	0.0041	0.0134	0.1879	0.0823	0.8240	0.2177	0.0488	0.1994	0.0825	0.0213	0.4563	0.1999
0.7888	0.7394	0.6299	0.0114	0.2652	0.2006	0.0917	0.6898	0.1950	0.0374	0.0423	0.0407	0.0175	0.0501	0.2268
0.0020	0.0000	0.0013	0.0466	0.4451	0.2104	0.0918	0.6978	0.1985	0.0374	0.0310	0.0382	0.0175	0.0355	0.1047
0.0952	0.6589	0.1981	0.0501	0.5948	0.2187	0.0698	0.5802	0.1769	0.1454	0.5314	0.2202	0.0849	0.9791	0.0938
0.0381	0.8258	0.1559	0.0928	0.9232	0.2465	0.0781	0.9604	0.2130	0.1217	0.7098	0.2227	0.1001	0.9492	0.1186
0.0420	0.4302	0.1109	0.0946	1.0239	0.2059	0.0782	0.9387	0.2119	0.0990	0.6005	0.1890	0.0873	1.1600	0.1253
0.1356	0.9856	0.2605	0.0756	0.7129	0.2311	0.0788	0.7738	0.1989	0.1210	0.6647	0.2164	0.1112	1.2803	0.0991
0.1295	1.0014	0.2567	0.0754	0.6655	0.2341	0.0788	0.7451	0.1965	0.1187	0.6486	0.2132	0.1066	1.2963	0.1018
0.7878	0.8042	0.6425	0.0155	0.2682	0.2075	0.0000	0.0721	0.0156	0.0134	0.1819	0.0492	0.0038	0.4422	0.1753
0.0041	0.2229	0.0428	0.0424	0.4387	0.2292				0.0134	0.1619	0.0453	0.0038	0.4422	0.2335
0.0999	0.7445	0.2070	0.0458	0.5866	0.2428				0.1182	0.6412	0.2122	0.0721	1.3128	0.2064
0.0361	0.8853	0.1593	0.0892	0.9553	0.2343				0.0933	0.7648	0.2031	0.0877	1.1167	0.2161
0.0378	0.6273	0.1268	0.0910	1.0391	0.2375				0.0655	0.6412	0.1644	0.0765	2.5685	0.2225
0.1370	1.2371	0.2761	0.0712	0.6764	0.2343				0.0913	0.6859	0.1869	0.0935	1.3797	0.1824
0.1309	1.3828	0.2781	0.0711	0.6562	0.2292				0.0891	0.6696	0.1954	0.0891	1.3128	0.1626
0.7830	0.7394	0.6267	0.0469	0.5591	0.2290				0.0000	0.0161	0.0036	0.0000	0.0140	0.1694
0.8566	0.7362	0.7408	0.0495	0.6246	0.2244				0.1186	0.5707	0.2036	0.0680	1.0848	0.1876
0.8311	0.8653	0.7209	0.0846	0.9173	0.2073				0.0933	0.7415	0.2008	0.0829	1.1091	0.2097
0.8019	0.8611	0.6853	0.0863	1.0384	0.2274				0.0667	0.5833	0.1581	0.0682	1.0626	0.2528
0.8647	0.9284	0.7936	0.0702	0.7876	0.2361				0.0909	0.6874	0.1915	0.0915	1.3115	0.2213
0.8723	0.9496	0.7975	0.0701	0.8596	0.2054				0.0887	0.6646	0.1876	0.0871	1.3282	0.1896
0.0928	0.6589	0.1964	0.0049	0.3394	0.1862				0.1186	0.5537	0.2012	0.0680	1.1466	0.1997

0.1264	0.6977	0.1193	0.0399	0.6247	0.1595	0.0859	0.6639	0.2280	0.3084	0.6747	0.3874	0.4186	0.5099	0.4297
0.1126	0.7138	0.1133	0.0402	0.4752	0.0546	0.0780	0.6756	0.1964	0.3694	0.6539	0.4409	0.3008	0.5039	0.2398
0.1389	0.6936	0.1165	0.0198	0.6092	0.0546	0.0830	0.6881	0.2069	0.2800	0.9502	0.3887	0.2832	0.6094	0.3272
0.1262	0.5735	0.1669	0.0480	0.6249	0.1961	0.0794	0.5473	0.2103	0.3996	0.6789	0.4471	0.3206	0.4941	0.3202
0.1273	0.5735	0.2056	0.0480	0.6249	0.0752	0.0790	0.5868	0.0501	0.4002	0.7423	0.4566	0.3199	0.4669	0.3032
0.0065	0.0889	0.2179	0.0000	0.0158	0.0546	0.0053	0.0891	0.0502	0.0109	0.2163	0.0558	0.0182	0.1167	0.3032
0.0283	0.0317	0.1937	0.1462	0.1182	0.0546	0.0361	0.0454	0.1827	0.0000	0.0136	0.0059	0.0953	0.1693	0.0227
0.0103	0.2675	0.1891	0.0000	0.1367	0.1587	0.0095	0.2100	0.2034	0.0109	0.2163	0.0684	0.0264	0.1431	0.0119
0.0009	0.0156	0.0741	0.0000	0.0158	0.0922	0.0000	0.0143	0.1838	0.0000	0.0136	0.0045	0.0042	0.0253	0.1582
0.0000	0.0093	0.1213	0.0000	0.0158	0.1095	0.0000	0.0071	0.1919	0.0000	0.0000	0.0015	0.0021	0.0168	0.1964
0.0506	0.6044	0.0916	0.0350	0.4752	0.1356	0.0731	0.6655	0.1945	0.1680	0.6529	0.2787	0.3017	0.6218	0.1399
0.0558	0.7649	0.0069	0.0327	0.1862	0.1358	0.0824	0.8240	0.0035	0.2123	0.8660	0.3340	0.1405	0.5765	0.1364
0.0753	0.7730	0.0076	0.0224	0.3435	0.0834	0.0788	0.5746	0.1773	0.0844	0.9232	0.1997	0.0941	0.4492	0.1396
0.0866	0.6699	0.1479	0.0404	0.4071	0.0834	0.0823	0.6714	0.1993	0.2722	0.5716	0.3854	0.2156	0.7352	0.1694
0.0866	0.6402	0.1379	0.0404	0.4346	0.1133	0.0829	0.6919	0.1670	0.2726	0.6267	0.3910	0.2157	0.6823	0.1937
0.0330	0.1171	0.2222	0.1462	0.0997	0.1580	0.0416	0.1315	0.1778	0.0109	0.2344	0.0622	0.1110	0.2419	0.3831
0.0126	0.3029	0.1404	0.0000	0.1182	0.1592	0.0120	0.2600	0.1817	0.0146	0.1981	0.0671	0.0396	0.2225	0.3371
0.0065	0.0924	0.1506	0.0000	0.0000	0.1396	0.0053	0.1025	0.1747	0.0109	0.2163	0.0590	0.0201	0.1175	0.4020
0.0065	0.0924	0.1539	0.0000	0.0000	0.1579	0.0053	0.0917	0.1928	0.0109	0.2163	0.0574	0.0160	0.1071	0.3759
0.0540	0.6487	0.1898	0.0350	0.5060	0.1047	0.0762	0.6536	0.1618	0.1810	0.5369	0.2621	0.3066	0.6812	0.2802
0.0582	0.8483	0.1809	0.0327	0.1664	0.1398	0.0858	0.8281	0.1749	0.2124	0.7076	0.3218	0.1406	0.6029	0.2747
0.0826	0.8043	0.2361	0.0224	0.3435	0.1047	0.0819	0.6323	0.1781	0.0885	0.8340	0.1886	0.1013	0.5052	0.2039
0.0897	0.7131	0.0878	0.0404	0.4071	0.1502	0.0856	0.7058	0.1985	0.2698	0.5282	0.3851	0.2205	0.7990	0.3809
0.0897	0.6737	0.0955	0.0404	0.4346	0.0833	0.0861	0.7410	0.1777	0.2702	0.5248	0.3851	0.2206	0.7636	0.1570
0.0390	0.2931	0.1153	0.1492	0.2076	0.0876	0.0462	0.2585	0.1809	0.0109	0.2163	0.0684	0.1178	0.2493	0.1627
0.0292	0.0414	0.0980	0.1462	0.0997	0.2105	0.0361	0.0554	0.1868	0.0000	0.0274	0.0074	0.0981	0.1728	0.2412
0.0283	0.0285	0.0855	0.1462	0.0997	0.0960	0.0361	0.0428	0.1850	0.0000	0.0136	0.0045	0.0953	0.1641	0.1621
0.0820	0.6065	0.0870	0.1874	0.6624	0.0876	0.1069	0.6851	0.1861	0.1680	0.6529	0.2787	0.3773	0.6414	0.1610
0.0878	0.7651	0.1430	0.1762	0.2872	0.0876	0.1171	0.8299	0.1855	0.2123	0.8660	0.3339	0.2129	0.5868	0.1581
0.1076	0.8141	0.1931	0.1638	0.3894	0.1396	0.1113	0.5974	0.1802	0.0844	0.9232	0.2016	0.1764	0.4545	0.3930
0.1152	0.6656	0.1858	0.1918	0.5205	0.1004	0.1168	0.6720	0.1848	0.2722	0.6007	0.3883	0.2870	0.7129	0.2703
0.1152	0.6361	0.1830	0.1897	0.4687	0.1002	0.1166	0.6984	0.0180	0.2726	0.6579	0.3939	0.2856	0.6506	0.2018
0.0093	0.2765	0.1857	0.0000	0.1182	0.1130	0.0095	0.2129	0.0944	0.0109	0.2163	0.0684	0.0251	0.1540	0.3251
0.0103	0.2631	0.1227	0.0000	0.1182	0.1086	0.0095	0.2129	0.0174	0.0109	0.2163	0.0668	0.0269	0.1407	0.3262
0.0541	0.6125	0.0749	0.0350	0.5716	0.0038	0.0776	0.6878	0.1260	0.1810	0.4837	0.2764	0.3065	0.7000	0.1536
0.0563	0.8465	0.0062	0.0327	0.2714	0.0708	0.0847	0.8659	0.1260	0.2076	0.6408	0.3249	0.1389	0.6213	0.1931
0.0826	0.9408	0.1301	0.0224	0.4796	0.1404	0.0859	0.6758	0.1214	0.0885	0.7947	0.2102	0.0966	0.4857	0.1336
0.0825	0.6747	0.1158	0.0404	0.4929	0.1457	0.0909	0.7370	0.0950	0.2855	0.5556	0.3857	0.2227	0.7770	0.1310
0.0825	0.6522	0.0727	0.0404	0.4632	0.1310	0.0902	0.7639	0.0944	0.2859	0.6094	0.3910	0.2257	0.7005	0.1359
0.0009	0.0188	0.0719	0.0000	0.0000	0.1496	0.0000	0.0167	0.1582	0.0000	0.0136	0.0030	0.0042	0.0084	0.1629
0.0506	0.5910	0.1496	0.0350	0.5060	0.0749	0.0731	0.6822	0.1855	0.1680	0.6529	0.2810	0.3049	0.6030	0.1658
0.0560	0.7727	0.1576	0.0327	0.1664	0.1402	0.0825	0.8517	0.1967	0.2123	0.8660	0.3340	0.1418	0.5853	0.3818
0.0763	0.7484	0.2205	0.0224	0.3435	0.1093	0.0788	0.5945	0.1821	0.0844	0.8790	0.1977	0.0941	0.4492	0.3325
0.0860	0.6743	0.1540	0.0404	0.4071	0.1457	0.0823	0.6764	0.1855	0.2722	0.5716	0.3882	0.2128	0.7352	0.4021
0.0860	0.6443	0.1602	0.0404	0.4346	0.0424	0.0830	0.7031	0.1626	0.2726	0.6267	0.3939	0.2157	0.6823	0.3772
0.0506	0.5975	0.1565	0.0350	0.5060	0.0465	0.0731	0.6589	0.2168	0.1680	0.6529	0.2787	0.3032	0.6225	0.2756

0.1049	0.8326	0.1675	0.0559	0.2524	0.1885	0.0676	0.8884	0.1943	0.1017	0.8288	0.2231	0.0827	0.5644	0.1707	0.0360	0.8258	0.1543	0.0737	1.1051	0.1735
0.0541	0.6464	0.2109	0.0559	0.3934	0.1885	0.0444	0.2748	0.2006	0.0700	0.6332	0.1668	0.0839	0.6041	0.1400	0.0399	0.4302	0.1094	0.0755	1.1580	0.0421
0.0692	0.8839	0.2092	0.1370	1.0282	0.1887	0.0410	0.9715	0.1546	0.0541	0.7125	0.1646	0.0744	0.6557	0.1824	0.1332	0.9856	0.2586	0.0605	0.6319	0.1830
0.0692	0.8654	0.2171	0.1370	1.0282	0.1976	0.0411	1.0265	0.1221	0.0555	0.7626	0.1711	0.0744	0.7686	0.1787	0.1270	1.0014	0.2548	0.0604	0.5320	0.1627
0.0809	0.6907	0.2294	0.0608	0.4753	0.1885	0.0314	1.2250	0.1220	0.1082	0.5958	0.2041	0.0856	0.5452	0.1569	0.1051	0.7080	0.2123	0.0773	0.9041	0.1768
0.0809	0.7696	0.2160	0.0415	0.6006	0.1885	0.0541	0.5729	0.2302	0.0725	0.6200	0.1711	0.0892	0.6152	0.1302	0.1130	0.8529	0.2302	0.0790	1.0026	0.1627
0.0712	0.6873	0.2142	0.1316	1.2388	0.2251	0.0061	0.6415	0.1601	0.0587	0.5834	0.1528	0.0769	0.6877	0.1684	0.1704	0.8658	0.2879	0.0640	0.8808	0.1475
0.0755	0.6801	0.1677	0.1316	1.2388	0.1885	0.0062	0.6355	0.1713	0.0575	0.5595	0.1485	0.0774	0.6954	0.1707	0.1652	0.8907	0.2859	0.0639	0.8226	0.1566
0.0577	0.7478	0.1805	0.0755	0.3934	0.2251	0.0848	0.9745	0.2123	0.0926	0.5990	0.1892	0.0032	0.0438	0.1372	0.0502	0.7241	0.1625	0.0016	0.0609	0.1612
0.0846	0.6923	0.1722	0.1106	0.8639	0.2841	0.025	0.8151	0.2184	0.0940	0.6114	0.1853	0.0391	0.6181	0.1703	0.1484	0.9812	0.2704	0.0891	0.7481	0.0514
0.0689	0.6231	0.2074	0.1106	0.8639	0.2841	0.0251	0.9178	0.0182	0.0953	0.5868	0.1831	0.0380	0.6885	0.1711	0.1421	0.9451	0.2586	0.0899	0.7624	0.1941
0.0576	0.8716	0.2057	0.1424	1.6636	0.2411	0.0607	1.0331	0.1545	0.0529	0.6255	0.1542	0.0431	0.6232	0.1633	0.1492	1.2321	0.2960	0.0890	0.7952	0.1798
0.0876	0.8534	0.0132	0.1424	1.6636	0.2455	0.0609	1.0924	0.1458	0.0533	0.6590	0.1585	0.0425	0.6988	0.1689	0.1429	1.2177	0.2900	0.0898	0.8266	0.1878
0.0568	0.0604	0.2134	0.0000	0.0000	0.2548	0	0.0751	0.1568	0.0034	0.0789	0.0201	0.0119	0.5807	0.0177	0.0041	0.1299	0.0293	0.0000	0.0910	0.1957

0.0933	0.7274	0.1991	0.0829	1.1091	0.2528	0.0558	0.7480	0.1998	0.0327	0.1664	0.1718	0.0825	0.8157	0.1963	0.2123	0.8660	0.3317	0.1417	0.5859	0.2767
0.0667	0.5833	0.1581	0.0682	1.1220	0.2105	0.0753	0.7645	0.1968	0.0224	0.3435	0.0587	0.0788	0.5689	0.2020	0.0844	0.9232	0.1978	0.0941	0.4496	0.1989
0.0909	0.6743	0.1899	0.0915	1.3961	0.2689	0.0866	0.6549	0.2504	0.0404	0.4071	0.0465	0.0823	0.6647	0.1054	0.2722	0.5716	0.3854	0.2135	0.7306	0.3784
0.0887	0.6518	0.1860	0.0871	1.4153	0.1854	0.0866	0.6257	0.1117	0.0404	0.4346	0.0465	0.0830	0.6850	0.1083	0.2726	0.6267	0.3910	0.2135	0.6780	0.1505
0.1363	0.7565	0.2404	0.0955	0.8275	0.1660	0.0526	0.8712	0.1167	0.0350	0.4788	0.1310	0.0881	0.7822	0.1410	0.2901	0.7309	0.3830	0.3194	0.5418	0.1570
0.1109	0.5956	0.1978	0.0722	0.8822	0.2090	0.0708	0.7449	0.1397	0.0324	0.6328	0.0966	0.0849	0.6175	0.1167	0.1627	1.0605	0.2994	0.3093	0.5608	0.2359
0.1166	0.6036	0.2058	0.0955	0.5449	0.1889	0.0716	0.6756	0.1183	0.0453	0.4202	0.1091	0.0889	0.6291	0.1072	0.2967	0.6128	0.3859	0.3712	0.5440	0.1584
0.1148	0.5918	0.2026	0.0997	0.5487	0.1892	0.0737	0.6306	0.1102	0.0453	0.4788	0.1265	0.0895	0.6717	0.1022	0.2971	0.6392	0.3859	0.3719	0.5160	0.1535
0.0779	0.5326	0.1600	0.0590	0.9831	0.1895	0.0785	0.8278	0.1117	0.0174	0.1879	0.1219	0.0866	0.7012	0.1798	0.1704	0.7352	0.3035	0.1393	0.4719	0.1515
0.0928	0.6472	0.1907	0.0617	0.7409	0.2340	0.0756	0.7168	0.1550	0.0199	0.3323	0.1345	0.0989	0.6710	0.2048	0.3228	0.5358	0.3648	0.2360	0.5928	0.3943
0.0919	0.6286	0.1875	0.0657	0.7096	0.2285	0.0777	0.6930	0.1976	0.0199	0.3573	0.1348	0.0956	0.6845	0.1950	0.3233	0.5054	0.3648	0.2375	0.5417	0.2723
0.0849	0.5577	0.1726	0.0680	0.7138	0.2146	0.1044	0.7814	0.2017	0.0249	0.3585	0.1134	0.0947	0.6418	0.1875	0.2502	0.5466	0.3636	0.2243	0.5982	0.1969
0.0811	0.5765	0.1718	0.0722	0.7964	0.2450	0.1044	0.7727	0.1912	0.0249	0.4682	0.1167	0.0950	0.6750	0.1922	0.2505	0.5166	0.3578	0.2265	0.5931	0.3239
0.0039	0.0576	0.0179	0.0038	0.0740	0.2487	0.0018	0.0451	0.1857	0.0000	0.0660	0.1265	0.0056	0.0652	0.1798	0.0000	0.0268	0.0102	0.0054	0.0926	0.3250

Table S9 Species, and GenBank accession number used for phylogenetic analysis in this study

Species	GenBank accession number	Order
Annulohypoxylon stygium	NC023117	**Xylariales**
Dematophora necatrix	NC087850	
Diaporthe nobilis	NC054216	**Diaporthales**
Neopestalotiopsis cubana	OQ707026	**Amphisphaeriales**
Pestalotiopsidaceae	NC031828	
Neurospora crassa	KY498478	**Sordariales**
Ceratocystis colombiana	NC056160	**Halosphaeriales**
Ceratocystis papillata	NC056159	
Ceratocystis lukuohia	NC056158	
Ceratocystis huliohia	NC056157	
Ceratocystis uchidae	NC056156	
Ceratocystis changhui	NC056155	
Ceratocystis polychroma	NC056154	
Ceratocystis albifundus	NC045185	
Ceratocystis fimbriata	MT331849	
Leptographium terebrantis	NC072635	**Ophiostomatales**
Raffaelea arxii	NC083271	
Leptographium aureum	OQ851464	
Leptographium wingfieldii	NC072634	
Leptographium procerum	NC072559	
Sporothrix schenckii	NC015923	
Grosmannia fruticeta	OQ851465	
Samsoniella hepiali	NC068092	
Beauveria malawiensis	KT201147	
Cordyceps brongniartii	EU100743	
Beauveria bassiana	KT201149	
Cordyceps bassiana	EU100742	
Beauveria pscudobassiana	OR545380	
Beauveria caledonica	kT201150	
Cordyceps tenuipes	MK234910	
Cordyceps cicadae	MH922223	
Cordyceps militaris	NC022834	
Lecanicillium muscarium	AF487277	
Paecilomyces hepiali	KJ764671	
Metarhizium anisopliae	AY884128	
Metarhizium robertsii	JELW01000367	
Metarhizium album	MW448543	
Epichloe typhina	KX066185	
Epichloe festucae	KX066186	
Epichloe uncinata	NC072721	
Epichloe stromatolonga	NC072719	
Epichloe siegelii	NC072718	
Moelleriella zhongdongii	PQ367230	

Hypocrella discoidea	PQ367247	**Hypocreales**
Moelleriella libera	PQ367225	
Moelleriella raciborskii	PQ367229	
Moelleriella gracilispora	PQ367224	
Moelleriella oxystoma	PQ367228	
Moelleriella sp.C9	PQ367226	
Moelleriella sp.C3	PQ367227	
Orbiocrella petchil	NC049079	
Metacordyceps chlamydosporia	NC022835	
Ophiocordyceps sinensis	KY622006	
Ophiocordyceps lanpingensis	MW342621	
Hirsutella rhossiliensis	KU203675	
Hirsutella thompsonii	MH367296	
Tolypocladium cylindrosporum	NC046839	
Tolypocladium inflatum	KY924883	
Tolypocladium ophioglossoides	KX455872	
Purpureocillium takamizusanense	NC061540	
Pleurocordyceps sinensis	NC067712	
Hirsutella vermicola	NC036610	
Hirsutella minnesotensis	NC027660	
Trichoderma hamatum	MF287973	
Trichoderma asperellum	KR952346	
Trichoderma gamsii	KU687109	
Trichoderma reesei	AF447590	
Trichoderma afroharzianum	NC065768	
Hypomyees aurantius	KU666552	
Paecilomyces penicillatus	MK069583	
Acremonium chrysogenum	KF757229	
Acremonium fuci	KR864757	
Clonostachys rosea	KU668563	
Clonostachys byssicola	NC072327	
Fusarium fujikuroi	JX910420	
Fusarium proliferatum	LT841261	
Fusarium mangiferae	KP742838	
Fusarium ussurianum	MW182675	
Gibberella moniliformis	JN041210	
Fusarium circinatum	JX910419	
Fusarium commune	LT906348	
Fusarium oxysporum	MF155191	
Fusarium gerlachii	KM486533	
Fusarium graminearum	DQ364632	
Fusarium culmorum	KP827647	
Fusarium solani	JN041209	
llyonectria destructans	KU881725	
Nectria cinnabarina	KT731105	
Stachybotrys chartarum	NC060508	

Stachybotrys chlorohalonata	NC060509	
Zelopaecilomyces penicillatus	NC043850	
Akanthomyces lecanii	OR506462	
Parengyodontium album	NC032302	
Lecanicillium saksenae	NC028330	
Pleurotus ostreatus	NC009905	**Out group**

Table S10 Sequence collinearity of mtDNA of seven species of *Moelleriella*

query	database	identity (%)	alignment	mismatch	gap	q. start	q. end	d. start	d. end	e-value	score
M.raciborskii	*M.oxystoma*	90.256	2309	166	15	2345	4647	28352	30607	0	2963
M.raciborskii	*M.oxystoma*	87.975	2445	228	34	40795	43218	5410	7809	0	2826
M.raciborskii	*M.oxystoma*	93.437	1417	85	4	17565	18981	56140	57548	0	2095
M.raciborskii	*M.oxystoma*	90.532	1542	96	26	38158	39684	2008	3514	0	1993
M.raciborskii	*M.oxystoma*	94.505	1274	65	4	7347	8619	36790	38059	0	1960
M.raciborskii	*M.oxystoma*	93.041	1164	76	3	32890	34049	85070	86232	0	1696
M.raciborskii	*M.oxystoma*	91.715	1219	71	21	28510	29720	80882	82078	0	1664
M.raciborskii	*M.oxystoma*	86.252	1542	195	12	20409	21947	60065	61592	0	1657
M.raciborskii	*M.oxystoma*	89.357	1306	137	2	12870	14173	45244	46549	0	1640
M.raciborskii	*M.oxystoma*	86.387	1168	117	28	5216	6381	32357	33484	0	1238
M.raciborskii	*M.oxystoma*	88.574	954	107	2	25901	26853	74402	75354	0	1157
M.raciborskii	*M.oxystoma*	91.165	747	54	6	43595	44337	9567	10305	0	1003
M.raciborskii	*M.oxystoma*	82.2	1191	177	23	19043	20214	57559	58733	0	992
M.raciborskii	*M.oxystoma*	93.525	556	33	3	34089	34643	86234	86787	0	824
M.raciborskii	*M.oxystoma*	90.878	581	50	2	25325	25905	72456	73033	0	776
M.raciborskii	*M.oxystoma*	88.272	648	68	7	23770	24412	70925	71569	0	769
M.raciborskii	*M.oxystoma*	82.359	924	121	27	10242	11132	42922	43836	0	765
M.raciborskii	*M.oxystoma*	81.244	965	133	27	9282	10231	41937	42868	0	736
M.raciborskii	*M.oxystoma*	89.547	574	60	0	23186	23759	69826	70399	0	728
M.raciborskii	*M.oxystoma*	92.873	463	33	0	21946	22408	62655	63117	0	673
M.raciborskii	*M.oxystoma*	86.312	621	68	9	34733	35351	89681	90286	0	660
M.raciborskii	*M.oxystoma*	86.86	586	54	16	35351	35917	92159	92740	0	634
M.raciborskii	*M.oxystoma*	85.155	613	79	11	26981	27585	75448	76056	4.3E-177	617
M.raciborskii	*M.oxystoma*	82.045	763	68	25	2	732	21008	21733	1.2E-167	586
M.raciborskii	*M.oxystoma*	84.006	644	63	24	15620	16241	54177	54802	1.6E-166	582
M.raciborskii	*M.oxystoma*	93.351	376	22	3	31009	31383	82079	82452	1.2E-157	553
M.raciborskii	*M.oxystoma*	91.117	394	34	1	1952	2344	26606	26999	1.6E-151	532
M.raciborskii	*M.oxystoma*	93.017	358	19	3	7078	7429	35402	35759	4.5E-147	518
M.raciborskii	*M.oxystoma*	92.308	364	28	0	15224	15587	53693	54056	4.5E-147	518
M.raciborskii	*M.oxystoma*	86.57	484	40	13	6605	7073	33618	34091	7.5E-145	510
M.raciborskii	*M.oxystoma*	85.941	441	42	15	1416	1837	23393	23832	1.3E-127	453
M.raciborskii	*M.oxystoma*	87.282	401	26	15	22514	22907	63191	63573	4.6E-122	435
M.raciborskii	*M.oxystoma*	90.06	332	33	0	14893	15224	52445	52776	6E-121	431
M.raciborskii	*M.oxystoma*	90.123	324	32	0	4652	4975	31920	32243	3.6E-118	422
M.raciborskii	*M.oxystoma*	95.019	261	8	4	43340	43598	8169	8426	3.6E-113	405
M.raciborskii	*M.oxystoma*	92.395	263	18	2	39681	39942	5148	5409	1E-103	374
M.raciborskii	*M.oxystoma*	82.71	428	48	16	8881	9284	40129	40554	1.03E-98	357
M.raciborskii	*M.oxystoma*	85.942	313	39	2	14249	14556	46585	46897	2.25E-90	329
M.raciborskii	*M.oxystoma*	92.105	228	18	0	27587	27814	77427	77654	3.76E-88	322
M.raciborskii	*M.oxystoma*	84.906	318	25	9	35915	36209	95334	95651	1.76E-81	300
M.raciborskii	*M.oxystoma*	93.299	194	13	0	14602	14795	49820	50013	1.37E-77	287
M.raciborskii	*M.oxystoma*	89.573	211	18	4	31361	31570	83371	83578	6.42E-71	265
M.raciborskii	*M.oxystoma*	92.073	164	13	0	20249	20412	58737	58900	6.51E-61	231
M.raciborskii	*M.oxystoma*	81.495	281	42	8	29747	30020	23892	24169	3.92E-58	222

M.raciborskii	*M.oxystoma*	84.135	208	33	0	27814	28021	79232	79439	5.11E-52	202
M.raciborskii	*M.oxystoma*	80.292	274	45	8	36573	36843	94842	95109	6.61E-51	198
M.raciborskii	*M.oxystoma*	91.667	132	11	0	23056	23187	67903	68034	1.85E-46	183
M.raciborskii	*M.oxystoma*	88.667	150	15	2	22909	23057	66496	66644	6.65E-46	182
M.raciborskii	*M.oxystoma*	94.783	115	4	2	31804	31917	85019	85132	8.61E-45	178
M.raciborskii	*M.oxystoma*	77.852	298	49	16	29748	30033	27029	27321	5.18E-42	169
M.raciborskii	*M.oxystoma*	94.393	107	3	2	44512	44615	12742	12848	8.67E-40	161
M.raciborskii	*M.oxystoma*	95.876	97	4	0	14794	14890	51189	51285	1.12E-38	158
M.raciborskii	*M.oxystoma*	83.06	183	20	6	945	1125	22378	22551	4.03E-38	156
M.raciborskii	*M.oxystoma*	90	120	12	0	1838	1957	25161	25280	4.03E-38	156
M.raciborskii	*M.oxystoma*	94.949	99	5	0	8621	8719	39986	40084	4.03E-38	156
M.raciborskii	*M.oxystoma*	86.822	129	17	0	29818	29946	25381	25509	8.73E-35	145
M.raciborskii	*M.oxystoma*	79.208	202	36	6	29748	29946	30642	30840	5.25E-32	135
M.raciborskii	*M.oxystoma*	97.436	78	2	0	36132	36209	35	112	1.89E-31	134
M.raciborskii	*M.oxystoma*	94.118	85	4	1	31577	31661	84839	84922	8.79E-30	128
M.raciborskii	*M.oxystoma*	93.902	82	5	0	34652	34733	88375	88456	1.14E-28	124
M.raciborskii	*M.oxystoma*	93.421	76	5	0	29867	29942	77804	77879	2.46E-25	113
M.raciborskii	*M.oxystoma*	89.655	87	7	2	44279	44365	12487	12571	3.18E-24	110
M.raciborskii	*M.oxystoma*	77.5	200	32	9	44808	44999	13379	13573	1.15E-23	108
M.raciborskii	*M.oxystoma*	75.099	253	43	14	45104	45355	20674	20907	1.92E-21	100
M.raciborskii	*M.oxystoma*	83.486	109	15	1	12134	12239	44688	44796	6.89E-21	99
M.raciborskii	*M.oxystoma*	84.314	102	11	3	44978	45074	20521	20622	8.92E-20	95.3
M.raciborskii	*M.oxystoma*	78.882	161	17	11	41642	41796	8462	8611	3.21E-19	93.5
M.raciborskii	*M.oxystoma*	90	70	7	0	16242	16311	56071	56140	1.15E-18	91.6
M.raciborskii	*M.oxystoma*	78.289	152	26	5	28224	28370	80537	80686	1.15E-18	91.6
M.raciborskii	*M.oxystoma*	84.211	95	11	4	10615	10707	51025	51117	4.15E-18	89.8
M.raciborskii	*M.oxystoma*	76.374	182	32	11	30254	30430	73592	73767	1.49E-17	87.9
M.raciborskii	*M.oxystoma*	82.857	105	9	5	20708	20806	33935	34036	5.37E-17	86.1
M.raciborskii	*M.oxystoma*	73.267	303	53	22	29749	30036	34120	34409	5.37E-17	86.1
M.raciborskii	*M.oxystoma*	85.057	87	9	4	10622	10706	45049	45133	5.37E-17	86.1
M.raciborskii	*M.oxystoma*	76.761	142	30	3	12697	12836	44981	45121	3.23E-14	76.8
M.raciborskii	*M.oxystoma*	76.129	155	31	5	6828	6982	77196	77344	3.23E-14	76.8
M.raciborskii	*M.oxystoma*	74.468	188	41	6	29759	29942	3545	3729	1.16E-13	75
M.raciborskii	*M.oxystoma*	82.927	82	7	1	45397	45471	20925	21006	1.94E-11	67.6
M.raciborskii	*M.oxystoma*	95.122	41	2	0	43394	43434	9498	9538	6.99E-11	65.8
M.raciborskii	*M.oxystoma*	82.667	75	11	2	29964	30037	30885	30958	6.99E-11	65.8
M.raciborskii	*M.oxystoma*	89.583	48	5	0	14556	14603	48226	48273	9.04E-10	62.1
M.raciborskii	*M.oxystoma*	85.484	62	6	2	17441	17499	77275	77336	9.04E-10	62.1
M.raciborskii	*M.oxystoma*	84.746	59	9	0	17021	17079	57297	57355	3.25E-09	60.2
M.raciborskii	*M.oxystoma*	84.746	59	9	0	25158	25216	71485	71543	3.25E-09	60.2
M.raciborskii	*M.oxystoma*	82.812	64	11	0	10209	10272	53381	53444	1.17E-08	58.4
M.raciborskii	*M.oxystoma*	83.607	61	9	1	6953	7012	60397	60457	4.21E-08	56.5
M.raciborskii	*M.oxystoma*	77.451	102	18	5	6128	6226	60762	60861	4.21E-08	56.5
M.raciborskii	*M.oxystoma*	84.483	58	6	3	17450	17505	55936	55992	1.51E-07	54.7
M.raciborskii	*M.oxystoma*	80	75	11	3	37077	37149	20916	20988	5.44E-07	52.8
M.oxystoma	*M.zhongdongii*	88.424	5788	575	60	28352	34091	11618	17358	0	6889

M.oxystoma	*M.zhongdongii*	93.282	2322	143	7	37767	40085	17455	19766	0	3411
M.oxystoma	*M.zhongdongii*	85.552	3267	413	45	74402	77654	38776	41997	0	3363
M.oxystoma	*M.zhongdongii*	86.322	2398	232	45	5441	7807	1	2333	0	2523
M.oxystoma	*M.zhongdongii*	84.267	2320	297	45	54181	56473	27679	29957	0	2200
M.oxystoma	*M.zhongdongii*	91.307	1576	114	16	80893	82452	42573	44141	0	2130
M.oxystoma	*M.zhongdongii*	87.96	1794	201	10	85013	86798	45565	47351	0	2102
M.oxystoma	*M.zhongdongii*	82.691	2334	344	46	59980	62271	31257	33572	0	2017
M.oxystoma	*M.zhongdongii*	90.561	1176	109	2	58766	59940	30075	31249	0	1555
M.oxystoma	*M.zhongdongii*	84.204	1608	167	46	66496	68034	35902	37491	0	1482
M.oxystoma	*M.zhongdongii*	87.314	1277	161	1	45268	46543	22004	23280	0	1459
M.oxystoma	*M.zhongdongii*	81.464	1489	239	23	49820	51285	24369	25843	0	1186
M.oxystoma	*M.zhongdongii*	81.101	963	134	26	41937	42871	20227	21169	0	726
M.oxystoma	*M.zhongdongii*	82.147	857	113	20	42922	43773	21177	21998	0	699
M.oxystoma	*M.zhongdongii*	87.097	589	73	2	72448	73033	38192	38780	0	664
M.oxystoma	*M.zhongdongii*	84.027	745	59	19	2008	2726	51467	52177	0	662
M.oxystoma	*M.zhongdongii*	95.63	389	17	0	5148	5536	56635	57023	3.2E-179	625
M.oxystoma	*M.zhongdongii*	88.544	515	44	7	9567	10078	3059	3561	9E-175	610
M.oxystoma	*M.zhongdongii*	88.655	476	51	3	62644	63118	35036	35509	9.1E-165	577
M.oxystoma	*M.zhongdongii*	88.675	468	25	12	3047	3514	56199	56638	2.6E-155	545
M.oxystoma	*M.zhongdongii*	81.269	646	77	21	89681	90286	47430	48071	2E-136	483
M.oxystoma	*M.zhongdongii*	85.809	451	54	6	92159	92605	48071	48515	1.6E-132	470
M.oxystoma	*M.zhongdongii*	84.342	479	72	3	71092	71570	37705	38180	2.1E-131	466
M.oxystoma	*M.zhongdongii*	87.056	394	50	1	26606	26999	11225	11617	9.6E-125	444
M.oxystoma	*M.zhongdongii*	88.315	368	41	2	53695	54061	27241	27607	1.3E-123	440
M.oxystoma	*M.zhongdongii*	90.909	319	29	0	52459	52777	26922	27240	2.7E-120	429
M.oxystoma	*M.zhongdongii*	89.881	336	21	8	21406	21733	7164	7494	1.6E-117	420
M.oxystoma	*M.zhongdongii*	95.669	254	6	2	8178	8426	2809	3062	1.6E-112	403
M.oxystoma	*M.zhongdongii*	90.083	242	24	0	40313	40554	19988	20229	7.89E-86	315
M.oxystoma	*M.zhongdongii*	86.434	258	35	0	23575	23832	10775	11032	2.22E-76	283
M.oxystoma	*M.zhongdongii*	90.233	215	21	0	69826	70040	37490	37704	8E-76	281
M.oxystoma	*M.zhongdongii*	80.612	392	45	19	63191	63574	35533	35901	1.34E-73	274
M.oxystoma	*M.zhongdongii*	90.625	192	9	2	2727	2918	53377	53559	2.92E-65	246
M.oxystoma	*M.zhongdongii*	87.255	204	21	5	46697	46897	24122	24323	1.06E-59	228
M.oxystoma	*M.zhongdongii*	96.923	130	4	0	2917	3046	54776	54905	6.36E-57	219
M.oxystoma	*M.zhongdongii*	87.568	185	10	2	95334	95518	50501	50672	6.41E-52	202
M.oxystoma	*M.zhongdongii*	81.102	254	42	6	79232	79481	41997	42248	8.29E-51	198
M.oxystoma	*M.zhongdongii*	79.771	262	43	6	23892	24151	54932	55185	8.34E-46	182
M.oxystoma	*M.zhongdongii*	80.392	204	39	1	30639	30841	54929	55132	1.82E-37	154
M.oxystoma	*M.zhongdongii*	80.488	205	32	5	38086	38289	53580	53777	2.35E-36	150
M.oxystoma	*M.zhongdongii*	89.655	116	12	0	57618	57733	29958	30073	8.46E-36	148
M.oxystoma	*M.zhongdongii*	80.203	197	35	4	66078	66272	52240	52434	1.09E-34	145
M.oxystoma	*M.zhongdongii*	92.784	97	7	0	35398	35494	17359	17455	1.42E-33	141
M.oxystoma	*M.zhongdongii*	79.5	200	37	3	23894	24090	13903	14101	5.09E-33	139
M.oxystoma	*M.zhongdongii*	97.468	79	2	0	34	112	50685	50763	6.59E-32	135
M.oxystoma	*M.zhongdongii*	97.468	79	2	0	95573	95651	50685	50763	6.59E-32	135
M.oxystoma	*M.zhongdongii*	88.889	108	9	3	12721	12826	4282	4388	3.07E-30	130

M.oxystoma	*M.zhongdongii*	84.615	130	14	5	93559	93683	49618	49746	1.43E-28	124
M.oxystoma	*M.zhongdongii*	82.192	146	18	4	38103	38241	52242	52386	6.64E-27	119
M.oxystoma	*M.zhongdongii*	92.5	80	6	0	88377	88456	47351	47430	8.58E-26	115
M.oxystoma	*M.zhongdongii*	80.667	150	24	3	80536	80683	42331	42477	1.11E-24	111
M.oxystoma	*M.zhongdongii*	85.185	108	13	3	13467	13574	4428	4532	1.44E-23	108
M.oxystoma	*M.zhongdongii*	76.442	208	40	7	25308	25510	54929	55132	1.86E-22	104
M.oxystoma	*M.zhongdongii*	76.119	201	43	4	34119	34315	13902	14101	2.4E-21	100
M.oxystoma	*M.zhongdongii*	85.714	98	9	4	60366	60458	17202	17299	8.64E-21	99
M.oxystoma	*M.zhongdongii*	85	100	11	4	40129	40227	19780	19876	8.64E-21	99
M.oxystoma	*M.zhongdongii*	89.333	75	8	0	23393	23467	10329	10403	1.12E-19	95.3
M.oxystoma	*M.zhongdongii*	75.962	208	37	10	34117	34316	54931	55133	1.12E-19	95.3
M.oxystoma	*M.zhongdongii*	75.758	198	40	7	62360	62553	33685	33878	4.02E-19	93.5
M.oxystoma	*M.zhongdongii*	83.81	105	8	4	33935	34036	31642	31740	1.45E-18	91.6
M.oxystoma	*M.zhongdongii*	77.439	164	28	6	10089	10246	4009	4169	5.2E-18	89.8
M.oxystoma	*M.zhongdongii*	86.667	75	10	0	83503	83577	44640	44714	2.42E-16	84.2
M.oxystoma	*M.zhongdongii*	78.947	133	20	4	27158	27290	55061	55185	2.42E-16	84.2
M.oxystoma	*M.zhongdongii*	75.127	197	37	7	66080	66273	17791	17978	8.71E-16	82.4
M.oxystoma	*M.zhongdongii*	82.796	93	12	4	51025	51116	21542	21631	3.13E-15	80.5
M.oxystoma	*M.zhongdongii*	82.292	96	13	4	43311	43404	25586	25679	3.13E-15	80.5
M.oxystoma	*M.zhongdongii*	74.272	206	43	7	36076	36276	40837	41037	1.13E-14	78.7
M.oxystoma	*M.zhongdongii*	79.646	113	19	4	73676	73786	55520	55630	1.13E-14	78.7
M.oxystoma	*M.zhongdongii*	80.909	110	10	6	8462	8562	801	908	4.05E-14	76.8
M.oxystoma	*M.zhongdongii*	84.932	73	11	0	23924	23996	36090	36162	1.46E-13	75
M.oxystoma	*M.zhongdongii*	74.737	190	37	10	3545	3729	54944	55127	1.46E-13	75
M.oxystoma	*M.zhongdongii*	74.742	194	36	12	66664	66856	54945	55126	1.46E-13	75
M.oxystoma	*M.zhongdongii*	93.75	48	3	0	48226	48273	24323	24370	5.24E-13	73.1
M.oxystoma	*M.zhongdongii*	86.364	66	9	0	87272	87337	40837	40902	5.24E-13	73.1
M.oxystoma	*M.zhongdongii*	92	50	4	0	25161	25210	11033	11082	1.88E-12	71.3
M.oxystoma	*M.zhongdongii*	84	75	11	1	24105	24179	14138	14211	1.88E-12	71.3
M.oxystoma	*M.zhongdongii*	72.926	229	50	11	73631	73853	12810	13032	6.78E-12	69.4
M.oxystoma	*M.zhongdongii*	85.714	63	9	0	66795	66857	14034	14096	2.44E-11	67.6
M.oxystoma	*M.zhongdongii*	86.154	65	6	3	50467	50530	52881	52943	2.44E-11	67.6
M.oxystoma	*M.zhongdongii*	84	75	5	5	77278	77350	29416	29485	8.77E-11	65.8
M.oxystoma	*M.zhongdongii*	78.302	106	17	4	33846	33951	41540	41639	3.15E-10	63.9
M.oxystoma	*M.zhongdongii*	84.375	64	9	1	12501	12563	4202	4265	1.13E-09	62.1
M.oxystoma	*M.zhongdongii*	81.333	75	14	0	22400	22474	9789	9863	1.13E-09	62.1
M.oxystoma	*M.zhongdongii*	75.862	145	21	11	66078	66218	53595	53729	1.13E-09	62.1
M.oxystoma	*M.zhongdongii*	79.57	93	13	6	41649	41738	53604	53693	1.13E-09	62.1
M.oxystoma	*M.zhongdongii*	84.375	64	6	3	9498	9561	2857	2916	4.08E-09	60.2
M.oxystoma	*M.zhongdongii*	78.218	101	16	5	41641	41738	17790	17887	4.08E-09	60.2
M.oxystoma	*M.zhongdongii*	83.077	65	11	0	53381	53445	21144	21208	4.08E-09	60.2
M.oxystoma	*M.zhongdongii*	79.762	84	15	2	45049	45131	21549	21631	4.08E-09	60.2
M.oxystoma	*M.zhongdongii*	72.249	209	52	6	73644	73849	40807	41012	4.08E-09	60.2
M.oxystoma	*M.zhongdongii*	76.154	130	19	8	94842	94967	50913	51034	1.47E-08	58.4
M.oxystoma	*M.zhongdongii*	72.772	202	43	12	77683	77879	54933	55127	1.47E-08	58.4
M.oxystoma	*M.zhongdongii*	78.022	91	19	1	22449	22538	9887	9977	5.28E-08	56.5

M.oxystoma	*M.zhongdongii*	84.483	58	8	1	27251	27308	14145	14201	5.28E-08	56.5
M.oxystoma	*M.zhongdongii*	78.824	85	16	2	50438	50520	32131	32215	5.28E-08	56.5
M.oxystoma	*M.zhongdongii*	78.723	94	12	7	24060	24148	36233	36323	5.28E-08	56.5
M.oxystoma	*M.zhongdongii*	81.081	74	8	4	27159	27229	14031	14101	1.9E-07	54.7
M.oxystoma	*M.zhongdongii*	83.607	61	6	4	45066	45124	25611	25669	1.9E-07	54.7
M.oxystoma	*M.zhongdongii*	77.551	98	16	6	3546	3640	36071	36165	1.9E-07	54.7
M.oxystoma	*M.zhongdongii*	74.265	136	31	4	4381	4514	55480	55613	1.9E-07	54.7
M.oxystoma	*M.zhongdongii*	83.929	56	7	2	24237	24290	12606	12661	6.83E-07	52.8
M.oxystoma	*M.zhongdongii*	72.778	180	40	7	40936	41109	40836	41012	6.83E-07	52.8
M.oxystoma	*M.zhongdongii*	75.676	111	24	3	4889	4999	41291	41398	6.83E-07	52.8
*M.*sp.C9	*M.*sp.C3	98.101	6057	81	13	43755	49789	34040	40084	0	10517
*M.*sp.C9	*M.*sp.C3	98.409	4210	46	4	23890	28092	14033	18228	0	7384
*M.*sp.C9	*M.*sp.C3	94.503	4530	155	34	11402	15875	4183	8674	0	6900
*M.*sp.C9	*M.*sp.C3	98.477	3086	35	7	50598	53675	44126	47207	0	5428
*M.*sp.C9	*M.*sp.C3	97.293	2808	52	5	40643	43433	28850	31650	0	4743
*M.*sp.C9	*M.*sp.C3	97.614	2724	56	3	34952	37675	24515	27229	0	4662
*M.*sp.C9	*M.*sp.C3	96.951	2394	57	4	29587	31979	19476	21854	0	4002
*M.*sp.C9	*M.*sp.C3	97.535	2272	40	4	5070	7325	52625	54896	0	3871
*M.*sp.C9	*M.*sp.C3	94.24	2396	46	27	7326	9651	1	2374	0	3576
*M.*sp.C9	*M.*sp.C3	96.623	2132	29	8	1082	3201	47208	49308	0	3498
*M.*sp.C9	*M.*sp.C3	98.69	1832	22	1	3198	5029	50794	52623	0	3249
*M.*sp.C9	*M.*sp.C3	98.493	1659	16	3	17946	19604	10737	12386	0	2916
*M.*sp.C9	*M.*sp.C3	94.787	1055	36	14	10376	11411	3109	4163	0	1626
*M.*sp.C9	*M.*sp.C3	99.32	882	6	0	20762	21643	12386	13267	0	1596
*M.*sp.C9	*M.*sp.C3	98.822	849	6	2	49750	50598	42183	43027	0	1509
*M.*sp.C9	*M.*sp.C3	98.684	760	9	1	17188	17947	8676	9434	0	1347
*M.*sp.C9	*M.*sp.C3	98.52	743	8	1	23091	23833	13265	14004	0	1308
*M.*sp.C9	*M.*sp.C3	93.671	474	30	0	43434	43907	32706	33179	0	710
*M.*sp.C9	*M.*sp.C3	99.401	334	2	0	29254	29587	18229	18562	6.3E-174	606
*M.*sp.C9	*M.*sp.C3	97.904	334	7	0	31979	32312	24087	24420	1.4E-165	579
*M.*sp.C9	*M.*sp.C3	96.333	300	11	0	39296	39595	28465	28764	5.1E-140	494
*M.*sp.C9	*M.*sp.C3	99.355	155	1	0	37674	37828	28309	28463	4.33E-76	281
*M.*sp.C9	*M.*sp.C3	84.946	186	22	4	15920	16101	49343	49526	1.26E-46	183
*M.*sp.C9	*M.*sp.C3	86.957	161	16	4	5	165	47537	47692	2.1E-44	176
*M.*sp.C9	*M.*sp.C3	95.745	94	4	0	33457	33550	24421	24514	3.54E-37	152
*M.*sp.C9	*M.*sp.C3	88.983	118	12	1	53711	53827	47413	47530	5.93E-35	145
*M.*sp.C9	*M.*sp.C3	80.303	198	34	4	42508	42703	47434	47628	5.93E-35	145
*M.*sp.C9	*M.*sp.C3	79.397	199	34	6	1308	1502	30729	30924	1.28E-31	134
*M.*sp.C9	*M.*sp.C3	92.405	79	4	2	10015	10093	3043	3119	6.01E-25	111
*M.*sp.C9	*M.*sp.C3	85.149	101	5	7	36439	36533	12133	12229	6.06E-20	95.3
*M.*sp.C9	*M.*sp.C3	85.149	101	5	7	19351	19447	26002	26096	6.06E-20	95.3
*M.*sp.C9	*M.*sp.C3	84.536	97	14	1	53732	53827	30729	30825	6.06E-20	95.3
*M.*sp.C9	*M.*sp.C3	81.373	102	17	1	3817	3916	8993	9094	4.72E-16	82.4
*M.*sp.C9	*M.*sp.C3	77.551	147	26	7	22748	22890	30731	30874	4.72E-16	82.4
*M.*sp.C9	*M.*sp.C3	84.81	79	12	0	34772	34850	14636	14714	1.7E-15	80.5
*M.*sp.C9	*M.*sp.C3	86.842	76	5	3	22943	23018	30893	30963	1.7E-15	80.5

*M.*sp.C9	*M.*sp.C3	82.955	88	15	0	549	636	48109	48196	1.7E-15	80.5
*M.*sp.C9	*M.*sp.C3	77.027	148	26	8	22748	22890	47436	47580	6.1E-15	78.7
*M.*sp.C9	*M.*sp.C3	83.544	79	11	2	34773	34850	17972	18049	2.84E-13	73.1
*M.*sp.C9	*M.*sp.C3	85.714	70	5	4	27856	27923	14657	14723	3.67E-12	69.4
*M.*sp.C9	*M.*sp.C3	85.294	68	9	1	24526	24592	17992	18059	3.67E-12	69.4
*M.*sp.C9	*M.*sp.C3	93.478	46	3	0	23858	23903	36161	36206	3.67E-12	69.4
*M.*sp.C9	*M.*sp.C3	89.091	55	2	3	46871	46924	36205	36256	4.75E-11	65.8
*M.*sp.C9	*M.*sp.C3	85.714	63	7	2	50294	50355	41113	41174	4.75E-11	65.8
*M.*sp.C9	*M.*sp.C3	78.431	102	20	1	17505	17606	51413	51512	4.75E-11	65.8
*M.*sp.C9	*M.*sp.C3	73.797	187	34	11	6	189	30833	31007	2.21E-09	60.2
*M.*sp.C9	*M.*sp.C3	94.737	38	1	1	23720	23757	18865	18901	7.95E-09	58.4
*M.*sp.C9	*M.*sp.C3	78.022	91	18	2	1942	2031	40475	40564	2.86E-08	56.5
*M.*sp.C9	*M.*sp.C3	88.889	45	5	0	17052	17096	54302	54346	2.86E-08	56.5
*M.*sp.C9	*M.*sp.C3	84.906	53	8	0	584	636	17410	17462	1.03E-07	54.7
*M.*sp.C9	*M.*sp.C3	80.822	73	11	3	36435	36506	23951	24021	1.03E-07	54.7
*M.*sp.C9	*M.*sp.C3	76.033	121	20	8	19356	19469	23960	24078	1.03E-07	54.7
*M.*sp.C9	*M.*sp.C3	84.746	59	4	4	31775	31831	23960	24015	1.03E-07	54.7
*M.*sp.C9	*M.*sp.C3	85.455	55	4	3	45921	45972	37164	37217	1.03E-07	54.7
*M.*sp.C9	*M.*sp.C3	81.538	65	12	0	366	430	40352	40416	1.03E-07	54.7
*M.*sp.C9	*M.*sp.C3	82.812	64	8	3	29070	29131	51944	52006	1.03E-07	54.7
*M.*sp.C9	*M.*sp.C3	79.518	83	10	5	7738	7813	6918	7000	3.7E-07	52.8
*M.*sp.C9	*M.*sp.C3	78.889	90	10	7	1584	1666	9139	9226	3.7E-07	52.8
*M.*sp.C9	*M.*sp.C3	74.809	131	25	7	1933	2059	11432	11558	3.7E-07	52.8
*M.*sp.C9	*M.*sp.C3	96.774	31	1	0	17651	17681	47710	47740	3.7E-07	52.8
M.libera	*M.raciborskii*	96.844	4594	57	27	13808	18347	40795	45354	0	7600
M.libera	*M.raciborskii*	94.872	4017	132	24	25545	29498	10791	14796	0	6209
M.libera	*M.raciborskii*	97.694	2992	40	11	37864	40828	25158	28147	0	5116
M.libera	*M.raciborskii*	97.888	2651	28	9	21539	24170	4529	7170	0	4560
M.libera	*M.raciborskii*	98.201	2001	23	5	19540	21539	1227	3215	0	3483
M.libera	*M.raciborskii*	99.052	1794	17	0	12014	13807	38149	39942	0	3219
M.libera	*M.raciborskii*	99.491	1573	8	0	1	1573	28148	29720	0	2861
M.libera	*M.raciborskii*	97.099	1310	14	8	33916	35201	21878	23187	0	2187
M.libera	*M.raciborskii*	96.332	1254	45	1	10684	11937	34957	36209	0	2060
M.libera	*M.raciborskii*	99.555	899	4	0	24646	25544	8839	9737	0	1639
M.libera	*M.raciborskii*	93.51	1094	61	8	32048	33140	15227	16311	0	1618
M.libera	*M.raciborskii*	97.231	903	8	6	1574	2459	31009	31911	0	1513
M.libera	*M.raciborskii*	91.674	1141	19	14	18420	19498	1	1127	0	1511
M.libera	*M.raciborskii*	88.089	806	72	8	2403	3190	32890	33689	0	935
M.libera	*M.raciborskii*	96.038	530	20	1	37419	37947	23882	24411	0	861
M.libera	*M.raciborskii*	98.077	468	7	2	24170	24637	8327	8792	0	813
M.libera	*M.raciborskii*	94.896	431	22	0	30700	31130	14794	15224	0	675
M.libera	*M.raciborskii*	95.215	418	18	2	4958	5374	34317	34733	0	660
M.libera	*M.raciborskii*	88.778	499	42	8	36286	36773	23186	23681	6.6E-172	599
M.libera	*M.raciborskii*	95.808	334	14	0	33140	33473	17565	17898	4.1E-154	540
M.libera	*M.raciborskii*	92.988	328	23	0	33591	33918	20278	20605	9E-136	479
M.libera	*M.raciborskii*	83.471	363	57	2	3222	3584	33689	34048	2.06E-92	335

M.libera	*M.raciborskii*	93.45	229	8	1	6194	6415	34733	34961	7.41E-92	333
M.libera	*M.raciborskii*	87.008	254	30	3	3586	3837	34088	34340	7.56E-77	283
M.libera	*M.raciborskii*	95.69	116	5	0	33474	33589	19102	19217	6.1E-48	187
M.libera	*M.raciborskii*	97.333	75	2	0	18345	18419	45397	45471	3.75E-30	128
M.libera	*M.raciborskii*	96.923	65	2	0	6	70	27170	27234	1.36E-24	110
M.libera	*M.raciborskii*	96.923	65	2	0	39850	39914	28153	28217	1.36E-24	110
M.libera	*M.raciborskii*	97.872	47	1	0	26737	26783	11997	12043	2.96E-16	82.4
M.libera	*M.raciborskii*	79.661	118	15	7	23909	24022	20703	20815	1.38E-14	76.8
M.libera	*M.raciborskii*	76.048	167	26	10	9063	9217	37207	37371	4.96E-14	75
M.libera	*M.raciborskii*	77.419	124	24	3	31678	31799	10151	10272	6.41E-13	71.3
M.libera	*M.raciborskii*	84.058	69	6	4	30563	30628	10641	10707	3.86E-10	62.1
M.libera	*M.raciborskii*	89.13	46	5	0	26066	26111	11398	11443	4.99E-09	58.4
M.libera	*M.raciborskii*	75.188	133	26	7	9063	9190	6135	6265	1.8E-08	56.5
M.libera	*M.raciborskii*	92.5	40	1	2	23939	23976	17462	17501	1.8E-08	56.5
M.libera	*M.raciborskii*	90.476	42	3	1	31163	31203	34159	34200	6.46E-08	54.7
M.libera	*M.raciborskii*	84.314	51	8	0	4745	4795	18867	18917	8.35E-07	51
M.gracilispora	*M.libera*	88.773	2396	197	39	43995	46346	13852	16219	0	2868
M.gracilispora	*M.libera*	88.258	2095	178	40	18278	20350	37800	39848	0	2444
M.gracilispora	*M.libera*	90.206	1654	150	5	54147	55800	19898	21539	0	2146
M.gracilispora	*M.libera*	90.764	1505	105	27	26381	27865	83	1573	0	1978
M.gracilispora	*M.libera*	90.625	1280	118	2	69202	70480	27588	28866	0	1698
M.gracilispora	*M.libera*	89.915	1180	115	3	72757	73934	29619	30796	0	1517
M.gracilispora	*M.libera*	88.327	1285	110	12	34695	35966	10684	11941	0	1506
M.gracilispora	*M.libera*	84.227	1585	174	42	77128	78687	31375	32908	0	1472
M.gracilispora	*M.libera*	83.43	1545	207	25	29973	31503	2322	3831	0	1389
M.gracilispora	*M.libera*	91.054	939	61	13	12503	13425	33984	34915	0	1247
M.gracilispora	*M.libera*	96.113	746	27	1	47320	48063	16348	17093	0	1216
M.gracilispora	*M.libera*	91.436	759	47	15	36403	37149	12012	12764	0	1026
M.gracilispora	*M.libera*	86.885	915	92	17	59026	59914	22404	23316	0	1000
M.gracilispora	*M.libera*	92.217	681	32	12	29154	29830	1574	2237	0	944
M.gracilispora	*M.libera*	94.799	596	24	6	39955	40545	12956	13549	0	922
M.gracilispora	*M.libera*	84.109	774	102	16	32963	33720	5076	5844	0	728
M.gracilispora	*M.libera*	93.9	459	28	0	67421	67879	25086	25544	0	693
M.gracilispora	*M.libera*	89.669	513	41	7	76520	77025	30814	31321	0	643
M.gracilispora	*M.libera*	78.794	1028	161	33	15637	16638	36286	37282	0	638
M.gracilispora	*M.libera*	91.796	451	37	0	57071	57521	21539	21989	1.5E-180	628
M.gracilispora	*M.libera*	94.293	403	23	0	3308	3710	33071	33473	3.2E-177	617
M.gracilispora	*M.libera*	86.965	537	52	9	34177	34699	5883	6415	2.5E-168	588
M.gracilispora	*M.libera*	86.578	529	66	2	16887	17411	37419	37946	1.5E-165	579
M.gracilispora	*M.libera*	87.11	481	38	12	60140	60608	23605	24073	7.2E-149	523
M.gracilispora	*M.libera*	95.806	310	12	1	42022	42330	13546	13855	1.2E-141	499
M.gracilispora	*M.libera*	88.756	418	28	11	65549	65965	24689	25088	5.6E-140	494
M.gracilispora	*M.libera*	93.051	331	23	0	20526	20856	39936	40266	3.4E-137	484
M.gracilispora	*M.libera*	94.881	293	15	0	63173	63465	24170	24462	2.1E-129	459
M.gracilispora	*M.libera*	87.328	363	36	5	70518	70877	28950	29305	7.5E-114	407
M.gracilispora	*M.libera*	92.884	267	16	1	72475	72738	29304	29570	3.5E-107	385

M.gracilispora	*M.libera*	87.209	344	26	11	57622	57948	22063	22405	2.1E-104	375
M.gracilispora	*M.libera*	91.259	286	8	3	50937	51205	17067	17352	7.6E-104	374
M.gracilispora	*M.libera*	90.175	285	28	0	68918	69202	25545	25829	2.8E-103	372
M.gracilispora	*M.libera*	89.643	280	27	2	15360	15638	34923	35201	2.77E-98	355
M.gracilispora	*M.libera*	95.337	193	8	1	38389	38580	12765	12957	2.83E-83	305
M.gracilispora	*M.libera*	94.819	193	10	0	9889	10081	33726	33918	3.65E-82	302
M.gracilispora	*M.libera*	88.261	230	27	0	22193	22422	40266	40495	2.22E-74	276
M.gracilispora	*M.libera*	82.779	331	29	12	51840	52165	18711	19018	1.03E-72	270
M.gracilispora	*M.libera*	93.827	162	10	0	1837	1998	32909	33070	6.25E-65	244
M.gracilispora	*M.libera*	90.798	163	15	0	52695	52857	19333	19495	3.79E-57	219
M.gracilispora	*M.libera*	94.815	135	7	0	8638	8772	33591	33725	6.34E-55	211
M.gracilispora	*M.libera*	72.46	748	167	26	6750	7469	35356	36092	1.06E-52	204
M.gracilispora	*M.libera*	82.547	212	37	0	23983	24194	40494	40705	1.07E-47	187
M.gracilispora	*M.libera*	96.296	108	4	0	65378	65485	24464	24571	6.43E-45	178
M.gracilispora	*M.libera*	92.241	116	9	0	31489	31604	4961	5076	5E-41	165
M.gracilispora	*M.libera*	92.553	94	7	0	4855	4948	33474	33567	3.92E-32	135
M.gracilispora	*M.libera*	89.691	97	10	0	61916	62012	24074	24170	8.49E-29	124
M.gracilispora	*M.libera*	78.462	195	32	6	25041	25228	40507	40698	3.95E-27	119
M.gracilispora	*M.libera*	89.011	91	10	0	53814	53904	19733	19823	1.84E-25	113
M.gracilispora	*M.libera*	91.892	74	5	1	51639	51712	17920	17992	3.98E-22	102
M.gracilispora	*M.libera*	85.87	92	13	0	16678	16769	37285	37376	5.15E-21	99
M.gracilispora	*M.libera*	91.429	70	6	0	11354	11423	33916	33985	1.85E-20	97.1
M.gracilispora	*M.libera*	81.905	105	13	6	10182	10281	23909	24012	1.44E-16	84.2
M.gracilispora	*M.libera*	78.947	133	19	9	39244	39373	9060	9186	5.18E-16	82.4
M.gracilispora	*M.libera*	82.796	93	11	4	6416	6505	30527	30617	6.7E-15	78.7
M.gracilispora	*M.libera*	82.222	90	14	2	37854	37942	9127	9215	2.41E-14	76.8
M.gracilispora	*M.libera*	79.57	93	15	1	57520	57608	14169	14261	1.88E-10	63.9
M.gracilispora	*M.libera*	80.233	86	13	3	21268	21349	5745	5830	6.75E-10	62.1
M.gracilispora	*M.libera*	84.127	63	10	0	68345	68407	31738	31800	6.75E-10	62.1
M.gracilispora	*M.libera*	92.683	41	3	0	31329	31369	31163	31203	2.43E-09	60.2
M.gracilispora	*M.libera*	75.758	132	23	9	59727	59855	9060	9185	8.73E-09	58.4
M.gracilispora	*M.libera*	82.812	64	11	0	37891	37954	29978	30041	8.73E-09	58.4
M.gracilispora	*M.libera*	83.582	67	6	4	68767	68830	30563	30627	8.73E-09	58.4
M.gracilispora	*M.libera*	84.483	58	7	2	37854	37910	18519	18575	3.14E-08	56.5
M.gracilispora	*M.libera*	80.822	73	11	3	39314	39384	18518	18589	1.13E-07	54.7
M.gracilispora	*M.libera*	96.875	32	1	0	51779	51810	18628	18659	1.13E-07	54.7
M.gracilispora	*M.libera*	84.906	53	8	0	59951	60003	23323	23375	1.13E-07	54.7
M.gracilispora	*M.libera*	77.778	90	16	4	54010	54096	19715	19803	4.06E-07	52.8
M.gracilispora	*M.libera*	94.286	35	1	1	38884	38917	22089	22123	4.06E-07	52.8
M.gracilispora	*M.libera*	78.313	83	17	1	60006	60088	23450	23531	4.06E-07	52.8
H.discoidea	*M.zhongdongii*	85.441	2720	335	34	35696	38380	37705	40398	0	2772
H.discoidea	*M.zhongdongii*	93.876	1731	85	10	39445	41163	42541	44262	0	2590
H.discoidea	*M.zhongdongii*	92.035	1833	137	7	13157	14984	13749	15577	0	2567
H.discoidea	*M.zhongdongii*	90.013	1552	130	12	796	2345	53377	54905	0	1984
H.discoidea	*M.zhongdongii*	88.385	1567	182	0	19057	20623	21718	23284	0	1886
H.discoidea	*M.zhongdongii*	87.5	1464	169	10	42546	43999	45673	47132	0	1677

H.discoidea	*M.zhongdongii*	88.399	1293	95	26	3090	4368	1	1252	0	1506
H.discoidea	*M.zhongdongii*	84.975	1411	144	41	24156	25536	26922	28294	0	1369
H.discoidea	*M.zhongdongii*	82.681	1507	213	36	22681	24155	24369	25859	0	1293
H.discoidea	*M.zhongdongii*	93.128	844	35	10	2346	3185	56199	57023	0	1216
H.discoidea	*M.zhongdongii*	83.32	1289	192	12	11870	13157	11222	12488	0	1168
H.discoidea	*M.zhongdongii*	85.286	1101	136	16	4406	5496	1251	2335	0	1112
H.discoidea	*M.zhongdongii*	91.994	712	34	11	5856	6544	2850	3561	0	977
H.discoidea	*M.zhongdongii*	90.234	727	70	1	18331	19056	19956	20682	0	948
H.discoidea	*M.zhongdongii*	84.409	744	97	12	47178	47914	47789	48520	0	713
H.discoidea	*M.zhongdongii*	86.405	662	66	14	15065	15722	15577	16218	0	702
H.discoidea	*M.zhongdongii*	92.089	493	28	5	306	795	51693	52177	0	684
H.discoidea	*M.zhongdongii*	89.277	429	44	2	27690	28117	29624	30051	8.5E-153	536
H.discoidea	*M.zhongdongii*	86.788	439	58	0	38383	38821	41769	42207	6.7E-139	490
H.discoidea	*M.zhongdongii*	81.835	556	63	20	32221	32772	35529	36050	1.2E-121	433
H.discoidea	*M.zhongdongii*	89.308	318	33	1	31789	32106	35201	35517	4.2E-111	398
H.discoidea	*M.zhongdongii*	89.298	299	32	0	45327	45625	47132	47430	2E-104	375
H.discoidea	*M.zhongdongii*	89.865	296	15	5	50721	51004	50475	50767	1.2E-101	366
H.discoidea	*M.zhongdongii*	89.3	243	23	3	20848	21089	24130	24370	3.38E-82	302
H.discoidea	*M.zhongdongii*	84.983	293	44	0	17682	17974	17455	17747	4.37E-81	298
H.discoidea	*M.zhongdongii*	91.163	215	19	0	34442	34656	37490	37704	2.03E-79	292
H.discoidea	*M.zhongdongii*	83.692	325	38	6	46866	47177	47430	47752	2.03E-79	292
H.discoidea	*M.zhongdongii*	93.264	193	13	0	29892	30084	31344	31536	3.4E-77	285
H.discoidea	*M.zhongdongii*	79.692	389	53	11	48929	49313	49618	49984	7.41E-69	257
H.discoidea	*M.zhongdongii*	81.388	317	51	6	11500	11812	10774	11086	3.45E-67	252
H.discoidea	*M.zhongdongii*	91.772	158	13	0	29734	29891	30052	30209	9.73E-58	220
H.discoidea	*M.zhongdongii*	84.804	204	30	1	13307	13509	54929	55132	9.8E-53	204
H.discoidea	*M.zhongdongii*	89.474	152	16	0	30150	30301	35046	35197	2.12E-49	193
H.discoidea	*M.zhongdongii*	83.019	212	16	14	103	307	51466	51664	7.68E-44	174
H.discoidea	*M.zhongdongii*	79.921	254	37	5	1002	1242	52219	52471	7.68E-44	174
H.discoidea	*M.zhongdongii*	90.152	132	13	0	32771	32902	37360	37491	2.76E-43	172
H.discoidea	*M.zhongdongii*	83.42	193	24	8	39065	39254	42330	42517	2.76E-43	172
M.samoensis	*M.zhongdongii*	95.238	105	5	0	41559	41663	44621	44725	1.29E-41	167
H.discoidea	*M.zhongdongii*	78.846	260	42	11	6555	6804	4009	4265	1.66E-40	163
H.discoidea	*M.zhongdongii*	76.899	316	49	13	50243	50558	50059	50350	7.74E-39	158
H.discoidea	*M.zhongdongii*	93.137	102	6	1	7269	7369	4287	4388	4.66E-36	148
H.discoidea	*M.zhongdongii*	81.283	187	29	5	1025	1205	17791	17977	1.67E-35	147
H.discoidea	*M.zhongdongii*	87.402	127	15	1	15725	15850	17328	17454	6.02E-35	145
H.discoidea	*M.zhongdongii*	98.649	74	1	0	8846	8919	7331	7404	4.69E-31	132
H.discoidea	*M.zhongdongii*	88.462	104	8	4	8697	8797	7213	7315	2.82E-28	122
H.discoidea	*M.zhongdongii*	94.595	74	4	0	18228	18301	19803	19876	4.72E-26	115
H.discoidea	*M.zhongdongii*	88.172	93	10	1	42279	42370	45563	45655	2.2E-24	110
H.discoidea	*M.zhongdongii*	76.446	242	32	24	44019	44242	36069	36303	7.9E-24	108
H.discoidea	*M.zhongdongii*	85.859	99	14	0	17976	18074	19667	19765	2.84E-23	106
H.discoidea	*M.zhongdongii*	92.857	70	5	0	25537	25606	29555	29624	3.68E-22	102
H.discoidea	*M.zhongdongii*	89.474	76	6	2	1267	1341	15550	15624	6.15E-20	95.3
H.discoidea	*M.zhongdongii*	84.946	93	10	4	23881	23972	21542	21631	7.96E-19	91.6

H.discoidea	*M.zhongdongii*	81.915	94	10	6	29565	29654	25582	25672	2.88E-13	73.1
H.discoidea	*M.zhongdongii*	81.928	83	13	2	29568	29649	21542	21623	3.73E-12	69.4
H.discoidea	*M.zhongdongii*	82.857	70	12	0	30082	30151	32812	32881	1.73E-10	63.9
H.discoidea	*M.zhongdongii*	89.583	48	5	0	50062	50109	50827	50874	6.24E-10	62.1
H.discoidea	*M.zhongdongii*	85	60	7	2	23322	23380	52881	52939	2.24E-09	60.2
H.discoidea	*M.zhongdongii*	86.538	52	7	0	9879	9930	9800	9851	8.07E-09	58.4
H.discoidea	*M.zhongdongii*	77.551	98	20	2	1629	1725	52846	52942	8.07E-09	58.4
H.discoidea	*M.zhongdongii*	77.66	94	19	2	13322	13414	36071	36163	2.9E-08	56.5
H.discoidea	*M.zhongdongii*	73.846	195	27	16	44022	44205	54946	55127	2.9E-08	56.5
H.discoidea	*M.zhongdongii*	79.348	92	8	9	11360	11448	10311	10394	1.04E-07	54.7
H.discoidea	*M.zhongdongii*	86	50	7	0	44410	44459	40591	40640	1.04E-07	54.7
H.discoidea	*M.zhongdongii*	87.5	48	5	1	42405	42452	45620	45666	1.04E-07	54.7
H.discoidea	*M.zhongdongii*	77.66	94	16	5	27589	27681	17231	17320	3.75E-07	52.8
H.discoidea	*M.zhongdongii*	84.615	52	8	0	13650	13701	36463	36514	3.75E-07	52.8
M.sp.C3	*M.gracilispora*	98.09	6754	112	8	25704	32441	9889	16641	0	11742
M.sp.C3	*M.gracilispora*	98.159	4183	69	5	32441	36622	16678	20853	0	7291
M.sp.C3	*M.gracilispora*	98.988	2867	14	5	48238	51102	39477	42330	0	5120
M.sp.C3	*M.gracilispora*	97.485	2743	42	4	14042	16771	68150	70878	0	4658
M.sp.C3	*M.gracilispora*	96.423	2684	65	15	44538	47207	34483	37149	0	4396
M.sp.C3	*M.gracilispora*	98.601	2430	31	2	51099	53526	43995	46423	0	4296
M.sp.C3	*M.gracilispora*	94.908	2612	85	20	5949	8553	53230	55800	0	4043
M.sp.C3	*M.gracilispora*	92.103	2862	170	35	36866	39708	25041	27865	0	3982
M.sp.C3	*M.gracilispora*	95.475	1967	68	12	8553	10503	57071	59032	0	3120
M.sp.C3	*M.gracilispora*	97.89	1564	17	3	18798	20345	77124	78687	0	2691
M.sp.C3	*M.gracilispora*	96.373	1544	18	18	20346	21854	1837	3377	0	2507
M.sp.C3	*M.gracilispora*	98.034	1373	11	4	53528	54896	46469	47829	0	2372
M.sp.C3	*M.gracilispora*	96.413	1366	28	8	42183	43531	29490	30851	0	2231
M.sp.C3	*M.gracilispora*	94.11	1460	86	0	16769	18228	72475	73934	0	2220
M.sp.C3	*M.gracilispora*	92.253	1536	60	15	619	2134	49633	51129	0	2122
M.sp.C3	*M.gracilispora*	97.111	1177	31	2	24526	25702	7598	8771	0	1982
M.sp.C3	*M.gracilispora*	98.333	900	15	0	10729	11628	59018	59917	0	1580
M.sp.C3	*M.gracilispora*	93.879	1062	27	13	47208	48237	38389	39444	0	1567
M.sp.C3	*M.gracilispora*	98.217	729	10	1	13265	13990	67421	68149	0	1271
M.sp.C3	*M.gracilispora*	99.24	658	5	0	11632	12289	59951	60608	0	1188
M.sp.C3	*M.gracilispora*	98.639	588	8	0	12680	13267	65378	65965	0	1042
M.sp.C3	*M.gracilispora*	98.553	553	8	0	18242	18794	76516	77068	0	977
M.sp.C3	*M.gracilispora*	91.523	696	33	9	3825	4516	52566	53239	0	935
M.sp.C3	*M.gracilispora*	92.397	605	30	6	1	595	47830	48428	0	848
M.sp.C3	*M.gracilispora*	99.198	374	3	0	39711	40084	29156	29529	0	675
M.sp.C3	*M.gracilispora*	98.555	346	5	0	43895	44240	31259	31604	2E-175	612
M.sp.C3	*M.gracilispora*	99.401	334	2	0	24087	24420	3377	3710	9.3E-174	606
M.sp.C3	*M.gracilispora*	97.179	319	8	1	43531	43848	30883	31201	3.4E-153	538
M.sp.C3	*M.gracilispora*	97.993	299	6	0	44240	44538	32963	33261	1.3E-147	520
M.sp.C3	*M.gracilispora*	97.952	293	6	0	12386	12678	63173	63465	2.7E-144	508
M.sp.C3	*M.gracilispora*	89.222	334	24	6	3109	3436	51999	52326	1E-113	407
M.sp.C3	*M.gracilispora*	97.297	222	6	0	36625	36846	22193	22414	7.9E-105	377

M.sp.C3	*M.gracilispora*	94.712	208	11	0	36854	37061	23984	24191	1.05E-88	324
M.sp.C3	*M.gracilispora*	98.37	184	2	1	3439	3622	52393	52575	3.77E-88	322
M.sp.C3	*M.gracilispora*	86.667	285	32	5	47413	47692	37188	37471	8.17E-85	311
M.sp.C3	*M.gracilispora*	74.119	653	133	22	4659	5307	75844	76464	1.41E-62	237
M.sp.C3	*M.gracilispora*	100	97	0	0	12290	12386	61916	62012	2.4E-45	180
M.sp.C3	*M.gracilispora*	81.07	243	29	12	47436	47678	63509	63734	8.64E-45	178
M.sp.C3	*M.gracilispora*	79.6	250	37	8	30731	30978	63509	63746	1.87E-41	167
M.sp.C3	*M.gracilispora*	82.632	190	23	10	49341	49526	27904	28087	3.13E-39	159
M.sp.C3	*M.gracilispora*	96.809	94	3	0	24421	24514	4855	4948	1.13E-38	158
M.sp.C3	*M.gracilispora*	76.632	291	52	11	30729	31007	37209	37495	2.44E-35	147
M.sp.C3	*M.gracilispora*	80.203	197	34	4	47435	47628	14929	15123	3.15E-34	143
M.sp.C3	*M.gracilispora*	79.798	198	33	7	49341	49536	60647	60839	1.47E-32	137
M.sp.C3	*M.gracilispora*	82.166	157	28	0	33023	33179	18269	18425	5.27E-32	135
M.sp.C3	*M.gracilispora*	77.455	275	31	22	49327	49590	31611	31865	5.27E-32	135
M.sp.C3	*M.gracilispora*	79.695	197	33	5	30729	30924	38615	38805	5.27E-32	135
M.sp.C3	*M.gracilispora*	89.32	103	10	1	35837	35939	5000	5101	8.83E-30	128
M.sp.C3	*M.gracilispora*	80.892	157	30	0	34036	34192	17256	17412	1.14E-28	124
M.sp.C3	*M.gracilispora*	91.304	92	6	2	3043	3132	51634	51725	1.14E-28	124
M.sp.C3	*M.gracilispora*	100	62	0	0	26001	26062	3165	3226	6.87E-26	115
M.sp.C3	*M.gracilispora*	85.149	101	5	7	12133	12229	10187	10281	8.95E-20	95.3
M.sp.C3	*M.gracilispora*	75.229	218	40	12	49330	49540	22451	22661	1.16E-18	91.6
M.sp.C3	*M.gracilispora*	84.158	101	6	7	26002	26096	60452	60548	4.16E-18	89.8
M.sp.C3	*M.gracilispora*	78.358	134	27	2	53765	53897	21285	21417	5.39E-17	86.1
M.sp.C3	*M.gracilispora*	74.771	218	41	14	49328	49537	43651	43862	5.39E-17	86.1
M.sp.C3	*M.gracilispora*	86.842	76	9	1	42587	42662	46421	46495	1.94E-16	84.2
M.sp.C3	*M.gracilispora*	89.706	68	1	4	21644	21707	22046	22111	6.97E-16	82.4
M.sp.C3	*M.gracilispora*	84.146	82	13	0	14634	14715	6425	6506	2.51E-15	80.5
M.sp.C3	*M.gracilispora*	86.842	76	5	3	30893	30963	67273	67348	2.51E-15	80.5
M.sp.C3	*M.gracilispora*	89.231	65	3	3	12133	12194	3166	3229	9.02E-15	78.7
M.sp.C3	*M.gracilispora*	80.392	102	18	1	8993	9094	44309	44408	3.24E-14	76.8
M.sp.C3	*M.gracilispora*	76.871	147	27	7	30731	30874	67080	67222	3.24E-14	76.8
M.sp.C3	*M.gracilispora*	81.818	88	16	0	48109	48196	37856	37943	1.17E-13	75
M.sp.C3	*M.gracilispora*	85.333	75	8	3	2169	2241	51124	51197	1.17E-13	75
M.sp.C3	*M.gracilispora*	87.143	70	4	4	17992	18059	68764	68830	1.17E-13	75
M.sp.C3	*M.gracilispora*	81.72	93	13	4	14634	14723	73674	73765	1.17E-13	75
M.sp.C3	*M.gracilispora*	81.72	93	12	4	17959	18049	6416	6505	4.19E-13	73.1
M.sp.C3	*M.gracilispora*	80.392	102	14	2	51413	51512	57511	57608	4.19E-13	73.1
M.sp.C3	*M.gracilispora*	76.351	148	27	8	47436	47580	67080	67222	4.19E-13	73.1
M.sp.C3	*M.gracilispora*	75.796	157	25	7	12044	12193	21962	22112	1.95E-11	67.6
M.sp.C3	*M.gracilispora*	85.714	63	7	2	41113	41174	30043	30104	7.02E-11	65.8
M.sp.C3	*M.gracilispora*	81.013	79	14	1	6765	6843	53816	53893	9.08E-10	62.1
M.sp.C3	*M.gracilispora*	75.54	139	26	7	50098	50232	56187	56321	9.08E-10	62.1
M.sp.C3	*M.gracilispora*	94.872	39	1	1	42590	42628	30850	30887	3.27E-09	60.2
M.sp.C3	*M.gracilispora*	75.556	135	26	6	40470	40601	39242	39372	3.27E-09	60.2
M.sp.C3	*M.gracilispora*	94.872	39	1	1	42589	42627	71655	71692	3.27E-09	60.2
M.sp.C3	*M.gracilispora*	100	31	0	0	9139	9169	38887	38917	1.17E-08	58.4

*M.*sp.C3	*M.gracilispora*	79.747	79	15	1	6554	6631	54027	54105	4.22E-08	56.5
*M.*sp.C3	*M.gracilispora*	84.483	58	6	3	23960	24015	3171	3227	1.52E-07	54.7
*M.*sp.C3	*M.gracilispora*	80.822	73	11	3	23951	24021	10183	10254	1.52E-07	54.7
*M.*sp.C3	*M.gracilispora*	85.455	55	4	3	37164	37217	20436	20487	1.52E-07	54.7
*M.*sp.C3	*M.gracilispora*	96.875	32	1	0	36219	36250	25357	25388	1.52E-07	54.7
*M.*sp.C3	*M.gracilispora*	81.538	65	12	0	40352	40416	37673	37737	1.52E-07	54.7
*M.*sp.C3	*M.gracilispora*	84.906	53	8	0	17410	17462	37891	37943	1.52E-07	54.7
*M.*sp.C3	*M.gracilispora*	75	128	26	6	11432	11556	39238	39362	1.52E-07	54.7
*M.*sp.C3	*M.gracilispora*	82.812	64	8	3	51944	52006	74912	74973	1.52E-07	54.7
*M.*sp.C3	*M.gracilispora*	91.892	37	3	0	43857	43893	31188	31224	5.46E-07	52.8
*M.*sp.C3	*M.gracilispora*	82.258	62	10	1	40301	40362	39085	39145	5.46E-07	52.8
*M.*sp.C3	*M.gracilispora*	96.774	31	1	0	47710	47740	57653	57683	5.46E-07	52.8
*M.*sp.C3	*M.gracilispora*	85.714	49	7	0	53755	53803	61233	61281	5.46E-07	52.8
*M.*sp.C3	*M.gracilispora*	92.105	38	2	1	18865	18901	68050	68087	5.46E-07	52.8
